# Expanding the diversity of bacterial DNA partitioning: A CTP-independent ParAB*S* system for plasmid partitioning in *Streptomyces*

**DOI:** 10.1073/pnas.2406398122

**Published:** 2025-07-02

**Authors:** Kirill V. Sukhoverkov, Francisco Balaguer-Perez, Clara Aicart-Ramos, Ngat T. Tran, Abbas Maqbool, Martin Rejzek, Govind Chandra, Fernando Moreno-Herrero, Tung B. K. Le

**Affiliations:** ^a^Department of Molecular Microbiology, John Innes Centre, Norwich NR4 7UH, United Kingdom; ^b^Department of Macromolecular Structures, Centro Nacional de Biotecnología, Consejo Superior de Investigaciones Científicas, Madrid 28049, Spain; ^c^Department of Biochemistry and Metabolism, John Innes Centre, Norwich NR4 7UH, United Kingdom

**Keywords:** DNA segregation, plasmid partition, ParABS, CTP, microbiology

## Abstract

The ATP- and CTP-dependent ParA-ParB-*parS* segrosome is a macromolecular complex that segregates chromosomes/plasmids in most bacterial species. CTP binding and hydrolysis enable ParB to slide along DNA and to bridge and condense DNA, thereby dictating the size and dynamics of the tripartite ParAB*S* complex. Several other evolutionarily distinct systems can also segregate DNA, although the full diversity of bacterial DNA partition systems remains unknown. Here, we identify a CTP-independent ParAB*S* system that maintains the conjugative plasmid SCP2 in the filamentous bacterium *Streptomyces coelicolor*. We demonstrate that an SCP2 ParB-like protein, ParT, loads onto DNA at an 18-bp *parS* site and diffuses away to the adjacent DNA despite lacking an apparent CTPase domain and detectable NTPase activity. We further show that *parS* DNA facilitates ParT transition from loading to a diffusing state, allowing ParT to accumulate on DNA, and that ParT activates the ATPase activity of its cognate partner protein, ParA. Additionally, we identify numerous structural homologs of ParT, suggesting that CTP-independent diffusion on DNA might be more common than previously recognized. Overall, our findings reveal a CTP-independent DNA translocation as an alternative and unexpected mechanism for assembling a bacterial DNA segregation complex and suggest that CTP binding and hydrolysis are not universal features of ParAB*S*-like systems.

## Significance

The ATP and CTP-dependent ParA-ParB-*parS* complex that actively partitions chromosomes and plasmids is widespread in bacteria. However, here, we provide evidence that CTP binding and hydrolysis might not be a universal feature of ParAB*S*-like systems by identifying a CTP-independent ParAT*S* system in a SCP2 plasmid from *Streptomyces coelicolor*. We show that the SCP2 ParB-like protein, ParT, can load at *parS* to diffuse to neighboring DNA to accumulate independently of nucleotide triphosphates. The local accumulation of ParT enables its binding and activation of a cognate SCP2 ParA to segregate the ParT-DNA complex, and subsequently the entire plasmid. Last, we identify many structural homologs of ParT that might have been diversified by evolution to perform new biological functions beyond DNA segregation.

DNA segregation is a fundamental process in biology that ensures faithful inheritance of chromosomes and plasmids. In bacteria, chromosomes and low-copy plasmids are actively transported to daughter cells by several evolutionarily distinct DNA partition systems (1–4). However, the full diversity of bacterial DNA partition systems remains unknown.

Among known bacterial DNA partition systems, the ATP and CTP-dependent type-I ParA-ParB-*parS* segrosome is the most widespread (1, 5). Type-I ParAB*S* systems consist of a centromere-like *parS* DNA sequence on the cargo DNA, a *parS*-binding CTPase protein ParB, and an ATPase protein ParA (1, 2). Type-I ParAB*S* segregates DNA by transporting a ParB-coated *parS* DNA cargo along a gradient of ParA-ATP via a DNA elasticity-assisted Brownian ratchet mechanism (2, 6–12). Briefly, ParB binds ParA and stimulates the ATPase activity of ParA, thereby dissociating the ParA-ATP dimer into individual ParA-ADP/apo monomers that no longer bind the nucleoid. This creates a local gradient of ParA-ATP, with the least amount of nucleoid-bound ParA-ATP near the ParB-*parS* DNA complex. The ParB-*parS* DNA complex then diffuses up the gradient via a Brownian-ratchet mechanism to rebind ParA-ATP, resulting in the net transportation of the ParB-*parS* DNA. The local depletion of nucleoid-bound ParA-ATP establishes a “no-return” zone, enforcing the unidirectional movement of the ParB-*parS* DNA complex. DNA-bound ParA-ATP complexes may exploit the elastic dynamics of the nucleoid to relay the partition complex from one DNA loop to another (12), resulting in long-range transportation of the ParB-*parS* complex.

Canonical type-I ParB functions as a self-loading DNA clamp that binds a 16-bp *parS* sequence on the bacterial chromosome or plasmid (13–15). CTP binding induces ParB self-dimerization at its N-terminal domain to close the clamp, while the C-terminal domain is a constitutive dimerization domain (15–17). Clamp closure allows *parS* DNA to transition from the *parS* DNA-binding domain to a DNA-storing lumen, effectively enabling ParB to escape high-affinity binding to the *parS* site and slide along the DNA to neighboring regions (14–18). Repeated ParB loading onto *parS*, followed by escape and sliding, results in multiple ParB-CTP clamps decorating the vicinity of the *parS* locus. Slow CTP hydrolysis reverses the switch, releasing the clamp from DNA and preventing ParB from diffusing too far from *parS* (16–18). Additionally, ParB-CTP has been suggested to phase-separate (19, 20) and recruit other ParB molecules to bridge and condense DNA surrounding *parS* sites (21–23). Altogether, these mechanisms create a high local concentration of ParB at the *parS* locus, activating the ATPase activity of ParA, thereby releasing ParA from the nucleoid to establish the no return zone and facilitate the segregation of replicated DNA to daughter cells (6, 12).

The prevailing model suggests that CTP binding and hydrolysis allow ParB to switch between multiple modes (loading at *parS* vs. sliding on DNA vs. DNA bridging/condensing/phase separation vs. releasing from DNA), which influence the size and activity of the ParA-ParB-*parS* segrosome. The identification of a CTP cofactor and the distinct CTP-dependent conformations adopted by ParB raised further questions, for instance, could there be alternative mechanisms for DNA clamping and ParB-like spreading that operate independently of CTP?

Here, we uncover a CTP-independent ParB-like protein, ParT (previously known as SCP2.04c), encoded on a low-copy circular plasmid SCP2 in *Streptomyces coelicolor* (24). The *parT* gene and an upstream *parA* gene were previously shown to contribute to the maintenance of SCP2 in *S. coelicolor* (24), however, the mechanistic details of ParT and ParA-mediated segregation of SCP2 remain unknown. By integrating biochemical assays, single-molecule in vitro reconstitution, chromatin immunoprecipitation with deep sequencing, and AlphaFold2-based structure prediction, we reveal that the N-terminal peptide of ParT binds to and activates the ATPase activity of its ParA partner. Furthermore, we demonstrate that ParT is a clamp-like protein capable of diffusing and accumulating on DNA independently of nucleotide triphosphates. We map the 18-bp ParT-loading *parS* site on SCP2 and show that ParT diffusion and accumulation on DNA are dependent on and are facilitated by its cognate *parS* site. Additionally, we propose a model for *parS*-dependent ParT clamp closure, potentially facilitated by resolving a steric clash between opposing ParT subunits binding to each *parS* half-site. Moreover, we identify ~100 structural homologs of ParT in bacterial species; however, those associated with a ParA-like ATPase partner mostly belong to the *Actinomycetota* phylum. Altogether, our findings provide insights into a previously unrecognized mode of bacterial DNA segregation that employs a CTP-independent DNA translocase.

## Results

### ParT Is a Type-I ParB-Like Protein That Lacks a CTPase Domain.

**Fig. 1.**
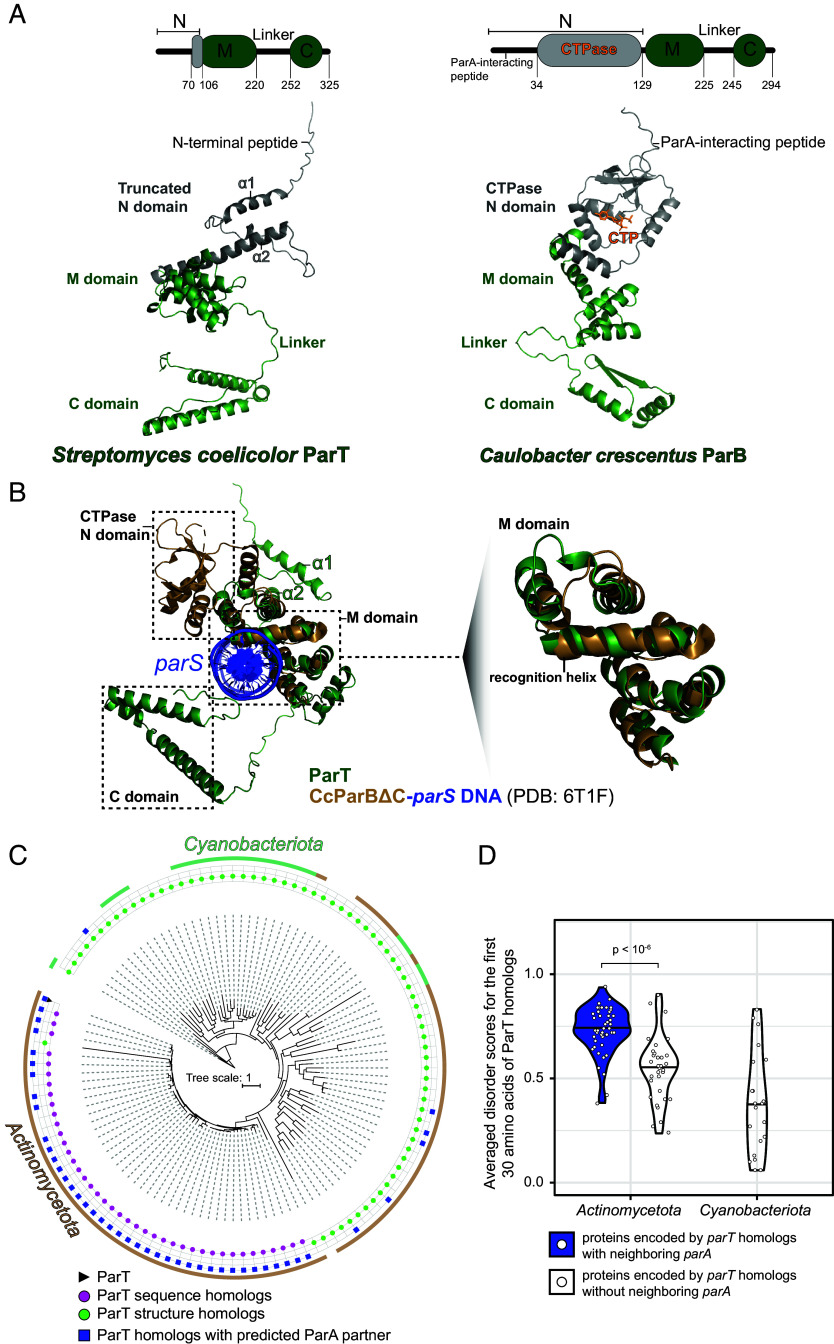
*S. coelicolor* ParT is a type-I ParB-like protein that lacks a CTPase domain. (*A*) The domain architecture of *S. coelicolor* ParT (*Left* Panel, UniProt ID: Q8VWE5) and *Caulobacter crescentus* ParB (*Right* Panel, UniProt ID: B8GW30) shows the N-terminal domain (N, gray), the middle DNA-binding domain (M, green), a C-terminal domain (C, green), and a linker connecting the M and C domains. A ParA-interacting motif resides in the N domain of *C. crescentus* ParB. Below the schematic diagrams are AlphaFold2-predicted structures of ParT and *C. crescentus* ParB. The positions of helices α1 and α2 are also indicated on the predicted structure of ParT. (*B*) (*Left*) Superimposition of the AlphaFold2-predicted structure of ParT with the *C. crescentus* ParB∆C-*parS* DNA complex (PDB: 6T1F) suggests that the ParT M domain is likely a *parS* DNA-binding domain. (*Right*) The recognition helix of *C. crescentus* ParB that binds to the *parS* site is indicated on the superimposition of the M domain only. (*C*) A phylogenetic tree showing the distribution of ParT sequence homologs (magenta) and structural homologs (green). Cases where a ParA-encoding gene was found in the genomic vicinity of *parT* homologs are indicated by blue squares. (*D*) Violin plots showing the predicted disorder probability of the first 30 N-terminal amino acids of proteins encoded by *parT* homologs with neighboring *parA* and those without neighboring *parA*.

To search for ParB-like proteins with novel features, we compiled a list of genetically validated ParAB*S* systems that have been previously described in the literature and inspected their predicted structures using AlphaFold2. Among the candidates, *S. coelicolor* SCP2.04c stands out as having the common features of a type-I ParB, such as a predicted flexible N-terminal peptide, a middle (M) domain with a helix–turn–helix motif, a C-terminal dimerization (C) domain, and a flexible linker connecting the M and C domains. However, SCP2.04c lacks a CTPase fold in the N-terminal region (Fig. 1*A* and *SI Appendix*, Fig. S1*A*), leading us to rename this truncated ParB-like protein as ParT to distinguish it from the canonical type-I ParB protein.

ParT and a *parS*-bound *Caulobacter crescentus* ParB∆CTD (lacking a C domain, PDB: 6T1F) (16) superimpose closely at the M domain, suggesting that the ParT M domain is also likely responsible for DNA binding (Fig. 1*B*). The N-terminal (N) domain of ParT is short, consisting of a predicted flexible N-terminal peptide, a helix α1, and a long helix α2 (Fig. 1 *A* and *B*). Notably, the predicted α1 has a low model confidence score (pLDDT < 50) (*SI Appendix*, Fig. S1*A*), while the predicted α2 is of very high confidence (pLDDT > 90) and is a common feature in structural homologs of ParT (Fig. 1*C* and Dataset S1).

To assess the prevalence of ParT, we searched the NCBI protein database using ParT amino acid sequence and found 41 sequence homologs (Fig. 1*C*). To retrieve more evolutionary distant ParT-like proteins, we employed FoldSeek to search for structural homologs of ParT in the AlphaFold Protein Structure Database (25) and retrieved 71 additional unique proteins (Fig. 1*C* and Dataset S2). ParT homologs predominantly belong to *Actinomycetota* (77 species) (Fig. 1*C*) and *Cyanobacteriota* phyla (21 species) (Fig. 1*C*). Next, to determine whether ParT-like proteins might be part of a DNA partition system, we searched for ParA-encoding gene in the vicinity of *parT* homologs, finding 45 such cases (Fig. 1*C* and Dataset S3). The majority of them (44, including *parT-parA*) belong to the *Actinomycetota* phylum (Fig. 1*C*). To test whether these ParT-like proteins can potentially interact with their ParA-like partners, we investigated whether these ParT-like proteins possess an unstructured N-terminal peptide, which is often required for ParA-interaction in the canonical type-I ParAB*S* system. To do so, we predicted the disorder probability of the first 30 N-terminus residues and compared the average disorder score between ParT-like proteins neighboring ParA to those that do not (Fig. 1*D*). Since most ParT-like proteins paired with ParA belong to *Actinomycetota* phylum, we limited our comparison to ParT homologs within this phylum. On average, proteins encoded by *parT* homologs located near *parA* genes exhibited significantly higher disorder probabilities in their N-terminal regions compared to those without a neighboring *parA* (Wilcoxon rank-sum test, *P* value = 1.9 × 10^−7^), suggesting that *parT* homologs with neighboring *parA* are likely components of functional DNA partition systems.

Overall, data suggest that *S. coelicolor* ParT is an atypical type-I ParB protein that lacks an apparent CTPase domain, and that ParT homologs, which may constitute DNA partition systems, are mostly confined to the *Actinomycetota* phylum.

### ParT Interacts with ParA in the Presence of ATP and Nonspecific DNA to Activate the ATPase Activity of ParA.

**Fig. 2.**
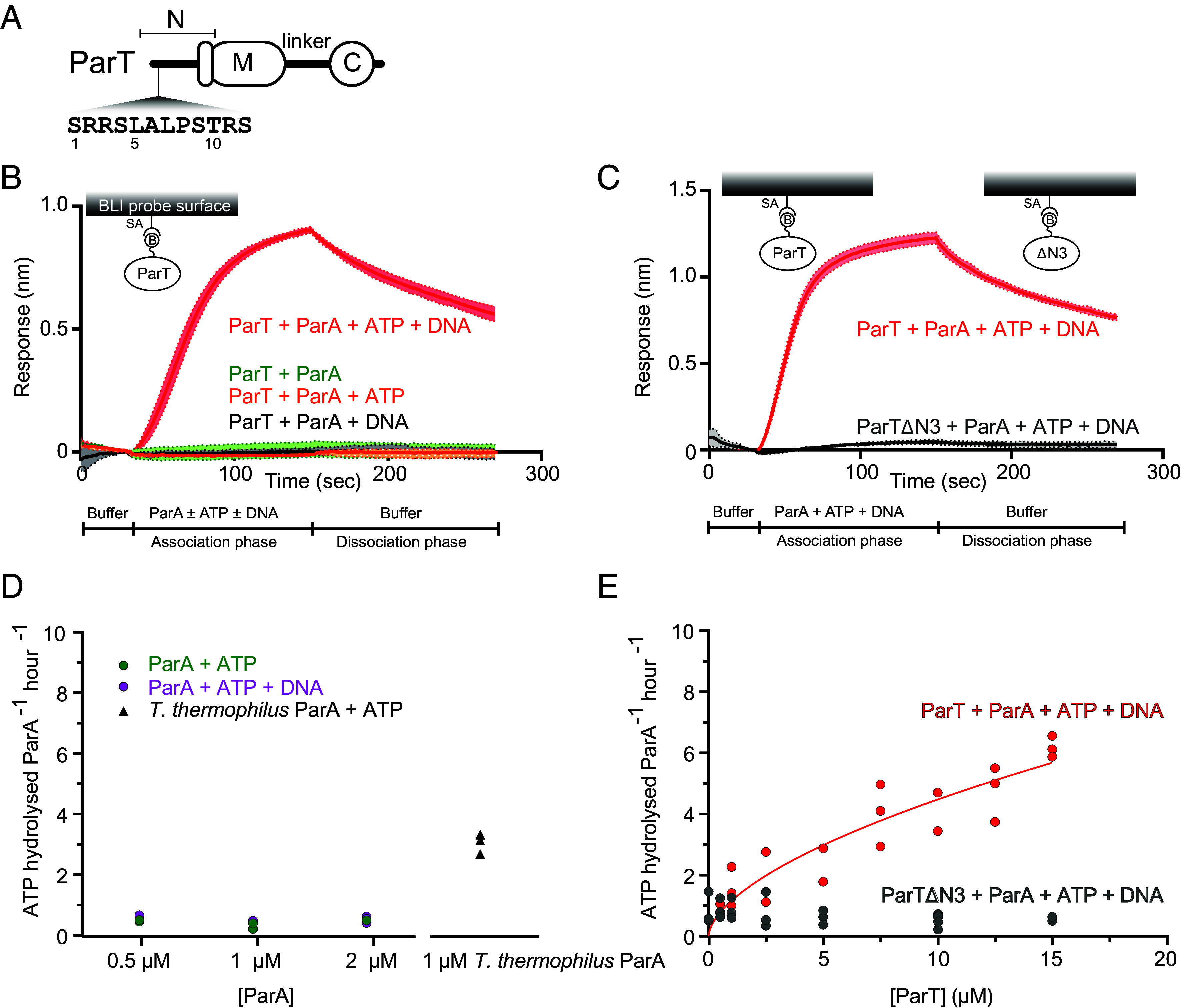
ParT interacts with ParA in the presence of ATP and nonspecific DNA to activate the ATPase activity of ParA. (*A*) The domain architecture of ParT (same as Fig. 1*A*), and the sequence of the first 12 amino acids of ParT is also shown. See also *SI Appendix*, Fig. S2 for the WebLogo representation of amino acid conservation at the N-terminus of ParT homologs. (*B*) ParT binds ParA in the presence of ATP and nonspecific DNA. BLI analysis of the interaction between 1 µM ParA with/without 1 mM ATP with/without 0.05 mg/mL salmon sperm DNA and biotinylated ParT that was immobilized on streptavidin (SA) BLI probes. Mean and SD (shading) from three replicates are shown. (*C*) The first three amino acids are required for ParA-ParT interaction. BLI analysis of the interaction between 1 µM ParA + 1 mM ATP + 0.05 mg/mL salmon sperm DNA and biotinylated ParT (WT) or a ParT∆N3 lacking the first three amino acids. Biotinylated ParT and ParT∆N3 were immobilized on streptavidin (SA) BLI probes. Mean and SD (shading) from three replicates are shown. (*D* and *E*) ParT stimulates the ATPase activity of ParA. Compared to a robust ATPase activity from *Thermus thermophilus* chromosomal ParA, 1 or 2 µM of SCP2 ParA by itself did not hydrolyze ATP regardless of the presence of DNA. (*E*) A molar excess of ParT, but not ParT∆N3, activated the ATPase activity of SCP2 ParA. ParA concentration was constant at 0.5 µM. All experiments were performed in triplicates.

To investigate whether *S. coelicolor* ParT, despite lacking the CTPase domain, binds to ParA (Fig. 2*A*), we examined the interaction of ParT with ParA in the presence or absence of ATP and nonspecific DNA substrate in real-time using biolayer interferometry (BLI). Biotinylated ParT was immobilized on a streptavidin-coated biosensor, and BLI monitored the shift in wavelength resulting from the change in probe optical thickness during the association and dissociation of ParA from immobilized ParT (Fig. 2*B*). In the presence of 1 µM of purified ParA alone or with 1 mM ATP or 0.05 mg/mL nonspecific DNA, no BLI signal was observed (Fig. 2*B*), suggesting that ParT did not bind to apo-ParA or ParA-ATP complex. However, premixing 1 µM ParA with ATP and nonspecific DNA increased the BLI response significantly (Fig. 2*B*), indicating that ParT binds to a ParA-ATP-DNA complex, consistent with the proposed role of ParT in DNA segregation (24). ParB proteins bind their partner ParA via an unstructured positively charged N-terminal peptide (6, 26–28). We found that the ParTΔN3 variant lacking the highly conserved first three amino acids (SRR) did not show a detectable response to ParA in the presence of ATP and nonspecific DNA (Fig. 2*C* and *SI Appendix*, Fig. S2). This indicates that, similar to canonical ParB, the N-terminal peptide of ParT is also essential for interaction with ParA.

Next, to investigate whether ParA-ParT interaction promotes ATP hydrolysis by ParA, we measured the ATPase activities of ParA in the presence of ATP, nonspecific DNA, and an increasing concentration of ParT. In the absence of ParT, 1 or 2 µM ParA did not detectably hydrolyze ATP, regardless of the presence of DNA. However, a positive control, *Thermus thermophilus* chromosomal ParA, at the same concentration, exhibited robust ATPase activity (Fig. 2*D*), consistent with a previous report (6). When ParT was added in increasing molar excess over 0.5 µM ParA, ATPase activity was robustly detected, reaching a rate of ~6 ATP/ParA/hour (Fig. 2*E*). However, the addition of ParTΔN3 did not result in the activation of ParA ATPase activity (Fig. 2*E*), likely owing to its inability to interact with ParA (Fig. 2*C*). Altogether, these data demonstrate that ParT, similar to a type-I ParB, interacts with ParA-ATP-DNA complex to activate the ATPase activity of its cognate partner, ParA.

### ParT Binds a Specific *parS* Site on SCP2.

**Fig. 3.**
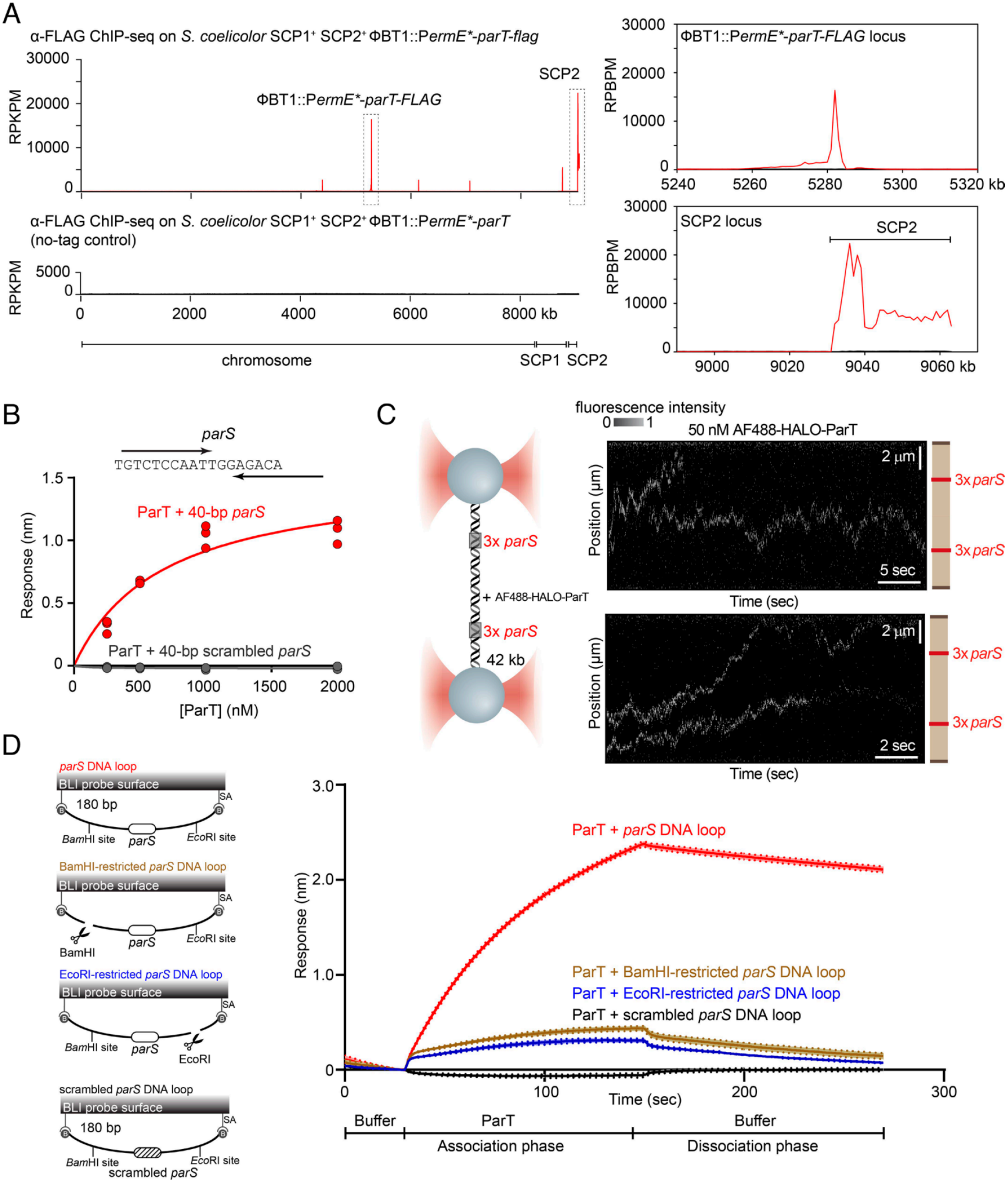
ParT diffuses on a *parS*-containing DNA. (*A*) α-FLAG ChIP-seq profiles show the enrichment of FLAG-tagged ParT on the SCP2 plasmid (*Lower Inset*) and at the ϕBT1 phage integration site on the chromosome (*Upper Inset*). Profiles were plotted with the x-axis representing genomic positions and the *y*-axis representing the number of reads per kilobase pair per million mapped reads (RPKPM). ChIP-seq experiments were performed twice using biological replicates, and a representative profile is shown. (*B*) BLI analysis of the interaction between increasing concentrations of ParT and 40-bp DNA duplexes containing either an 18-bp *parS* site or a scrambled *parS* site. BLI responses (nm) were defined as the maximal BLI signal during the association phase. See also *SI Appendix*, Table S1 *f*or K_D_ values of ParT-*parS/*scrambled *parS* interactions when surface plasmon resonance (SPR) was used instead of BLI. A nucleotide sequence of *parS* is also shown, and convergent arrows indicate that *parS* is an inverted repeat. (*C*) ParT diffuses on *parS* DNA without an additional NTP cofactor. (*Left* Panel) Schematic of the C-trap optical tweezers experiments where a ~42-kb DNA containing two clusters of 3x *parS* was tethered between two beads and scanned with a confocal microscope using 488 nm illumination. (*Right* Panel) Two representative kymographs showing AlexaFluor 488 (AF488)-HALO-ParT binding and diffusing on *parS*-containing DNA. See also *SI Appendix*, Fig. S3*B* for additional representative images. (*D*) ParT can accumulate on a closed *parS* DNA loop but not on a *parS* DNA with an open end. BLI analysis of the interaction between 1 µM ParT with a 180-bp dual biotin-labeled DNA that contains either a *parS* or a scrambled *parS* site. Interactions between a dual biotinylated DNA and a streptavidin (SA)-coated probe created a closed DNA loop where both ends were blocked (see the schematic diagram of the BLI probes on the left panel). 180-bp *parS* DNA loops were subsequently restricted by EcoRI or BamHI to generate an open free end. Mean and SD (shading) from three replicates are shown.

Next, to identify the *parS* site on SCP2, we performed chromatin immunoprecipitation with deep sequencing (ChIP-seq) to map DNA bound by a FLAG-tagged ParT which was expressed ectopically from a ϕBT1 phage integration site on the *S. coelicolor* A3(2) chromosome (Fig. 3 *A*, *Top Left* Panel). *S. coelicolor* with a nontagged ParT expressed from the same integration site was also employed as a negative control to eliminate false signals that might arise from cross-reaction with the α-FLAG antibody (Fig. 3 *A*, *Bottom Left* Panel). ChIP-seq revealed enrichment across the length of SCP2 with a peak centered on the *parT* gene on this plasmid, and another prominent asymmetrical ~5-kb-wide peak at the ectopic *parT-flag* allele engineered at the ϕBT1 phage integration site on the chromosome (Fig. 3 *A*, *Right* Panel). Given that the ChIP-seq data suggested that the *parS* site might reside within the coding sequence of ParT, we closely inspected the nucleotide sequence directly underneath the ChIP-seq summits, revealing an 18-bp inverted repeat, likely representing the core *parS* sequence (Fig. 3*B*). To confirm the *parS* sequence, we evaluated the binding of purified ParT to a 40-bp linear DNA duplex containing the 18-bp inverted repeat using BLI. Our data demonstrated that ParT specifically binds to *parS*-containing DNA duplexes (K_D_ = 680 ± 200 nM), but not to scrambled *parS* DNA duplexes (Fig. 3*B*), consistent with the ChIP-seq data.

### ParT Diffuses and Accumulates on a *parS* DNA Loop Independently of CTP.

Next, we further investigated the interaction between ParT and DNA using a longer 42-kb *parS*-containing DNA and dual optical tweezers combined with confocal fluorescence microscopy (29). Here, individual *parS*- or non-*parS*-containing DNA molecules were immobilized between two polystyrene beads and extended almost to their contour length under force (Fig. 3*C*, and *SI Appendix*, Fig. S3). To amplify the ParT DNA-binding signal, three *parS* sites were engineered into two clusters on the DNA (Fig. 3*C*). We incubated DNA with 50 nM of AlexaFluor (AF) 488-labeled HALO-tagged ParT and captured confocal images over time to generate kymographs. When a non-*parS* DNA was used, no fluorescence signal was observed on DNA (*SI Appendix*, Fig. S3*A*), consistent with the requirement of *parS* for ParT loading onto DNA. In contrast, when a *parS* DNA was used, kymographs displayed fluorescence signal from AF488-HALO-ParT outside of the *parS* clusters and along the length of the DNA (Fig. 3*C*, and *SI Appendix*, Fig. S3*B* for additional representative kymographs), indicating that ParT now distributed nonspecifically along the DNA. Next, we measured the position of individual AF488-HALO-ParT along the DNA over time to determine a diffusion constant (*D*) for ParT of 1.11 ± 0.05 µm^2^/sec (*SI Appendix*, Fig. S3 *C*–*E*). This rate is comparable to the diffusion constants previously determined for *B. subtilis* ParB-CTP and a ParB-like KorB-CTP diffusion on DNA (29, 30). These data indicate that ParT can diffuse along a *parS* DNA substrate in vitro without additional nucleotide triphosphates (NTPs). Further nucleotide content analysis by liquid chromatography with tandem mass spectrometry confirmed that ParT did not copurify with prebound NTPs (*SI Appendix*, Fig. S4), supporting our finding that ParT diffusion along a *parS* DNA substrate is independent of NTPs.

Next, to determine whether ParT diffusion leads to its accumulation on *parS* DNA, we monitored the interaction of ParT with *parS* DNA in real-time using BLI, which allowed us to use a higher concentration of purified ParT than in our optical tweezers setup. We employed a 180-bp dual biotin-labeled *parS* or non-*parS* DNA tethered at both ends to a streptavidin-coated probe to form a closed DNA loop (13, 16–18, 31) (Fig. 3*D*). With a non-*parS* DNA substrate, no BLI signal was detectable when the probe was incubated with 1 µM ParT, consistent with a *parS*-specific loading of ParT (Fig. 3*D*). However, with a cognate *parS* DNA substrate, incubation with 1 µM ParT resulted in a significant BLI response (Fig. 3*D*). Previous reports showed that CTP strongly promotes the association of type-I ParB proteins with closed *parS* DNA substrates (13, 15, 17, 29). Here, premixing ParT with ATP, CTP, GTP, or UTP did not change the BLI profile markedly (*SI Appendix*, Fig. S5*A*), consistent with ParT lacking an apparent CTPase domain as well as any detectable NTPase activity (*SI Appendix*, Fig. S5*B*). We further observed that accumulated ParT on DNA loops were released very slowly with a dissociation rate (k_off_) of ~3.5x10^-3^ s^-1^ when the probe was returned to a buffer-only solution (dissociation phase, Fig. 3*D*).

Next, we investigated whether a DNA substrate with a free end could support high ParT association. The 180-bp dual biotin-labeled DNA loop was designed with a BamHI and an EcoRI recognition sites flanking the *parS* site. The DNA-loop probe was immersed in either a BamHI- or an EcoRI-containing buffer to enable DNA restriction to generate a free end (Fig. 3*D*). We found that incubation of a restricted DNA probe with purified ParT reduced the BLI signal by ~fivefold, suggesting that ParT diffuses on the DNA and, similar to canonical ParB (13), slides off the free DNA end.

Fluorescently labeled ParB forms bright foci in vivo due to the clustering of multiple ParB molecules around the *parS* site. To determine whether ParT also accumulates in vivo, we visualized an mCherry-tagged ParT expressed either in *S. coelicolor* A3(2) carrying the SCP2 plasmid or a plasmid-free *S. coelicolor* M600 strain (*SI Appendix*, Fig. S6). To ensure that the only *parS* site present was on the SCP2 plasmid, the internal *parS* site within the coding sequence of *mCherry*-tagged *parT* was mutated while maintaining the coded amino acid sequence. We observed mCherry foci along the hyphae exclusively in the SCP2-harboring strain, but not in the plasmid-free *S. coelicolor* strain (*SI Appendix*, Fig. S6), indicating that, like other type-I ParB proteins, ParT also accumulates in vivo in a plasmid-dependent manner.

### ParT Does Not Condense *parS* DNA In Vitro.

The canonical type-I ParB was previously reported to bridge and condense *parS* DNA in vitro in the presence of CTP (22, 23, 29). We wondered whether ParT could also condense DNA, despite lacking a CTPase domain. To investigate this, we employed magnetic tweezers which allow simultaneous measurement of multiple DNA molecules and application of a lower force than optical tweezers. Single DNA molecules containing a cluster of 5x *parS* sites were immobilized between a glass surface and magnetic beads (*SI Appendix*, Fig. S7 *A* and *B*). A force of 5 pN was applied to the beads to stretch *parS* DNA between a pair of magnets before the addition of buffer only or 500 nM of purified ParT. The force was then gradually lowered to 0.006 pN, a level permissive to DNA condensation, and the extension of the tethered DNA was monitored in real-time. We observed that while *B. subtilis* ParB robustly condenses DNA in the presence of 2 mM CTP, there was no difference in the DNA extension over a range of forces in the presence or absence of ParT (*SI Appendix*, Fig. S7*C*), indicating that ParT did not condense DNA under the tested condition. The absence of condensation activity in ParT might help reduce entanglement among SCP2 copies after replication.

### ParT Self-Dimerization Is Facilitated by *parS* DNA In Vitro.

**Fig. 4.**
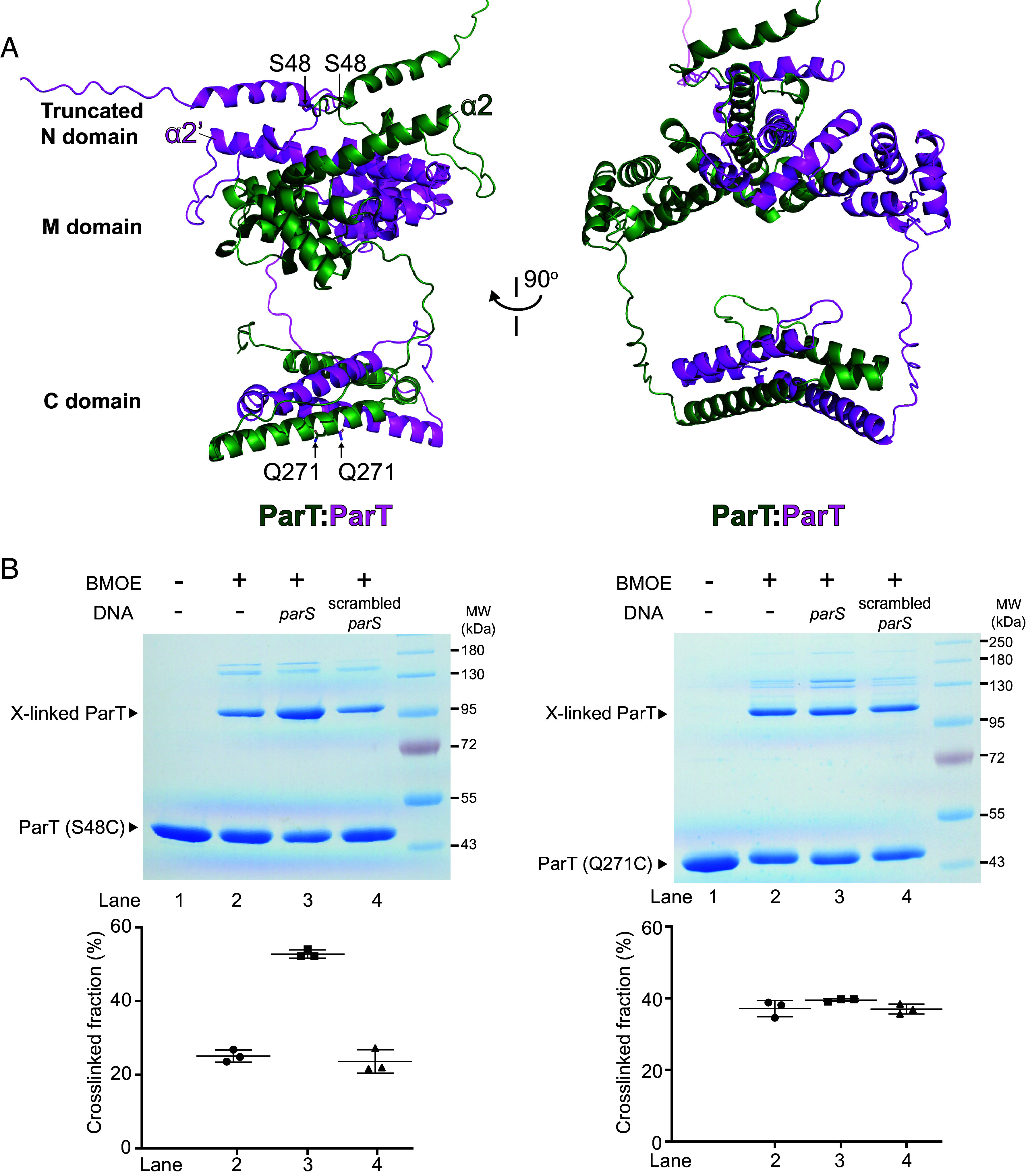
ParT self-dimerization at the N domain, but not the C domain, is promoted by *parS* DNA in vitro. (*A*) AlphaFold2-predicted structure of ParT dimer. The positions of opposing helices α2 and α2′ are also shown on the predicted structure of ParT dimer. Residues S48 at the N domain and Q271 at the C domain of ParT were individually substituted by cysteine for use in BMOE crosslinking assays. (*B*) SDS-PAGE analysis of BMOE crosslinking products of 4 µM ParT (S48C) or ParT (Q271C) with/without 4 μM of 40-bp *parS* DNA duplex or scrambled *parS* DNA duplex. The quantification of the major crosslinked fraction is shown below each representative image. Experiments were performed in triplicates and mean ± SD were quantified. See also *SI Appendix*, Fig. S9*C* where substoichiometric concentrations of *parS* were sufficient to promote crosslinking of ParT (S48C).

In the presence of CTP, ParB self-dimerizes at the N-terminal CTPase domain, forming a clamp-like molecule that diffuses on DNA (15–17, 32). We wondered whether ParT could form a functionally similar clamp, despite lacking a CTPase domain. To investigate this possibility, we attempted to crystallize apo-ParT or a ParT-*parS* cocomplex but were unsuccessful. Given that apo-ParT exists as a dimer in solution (*SI Appendix*, Fig. S8*A*), we instead employed AlphaFold2 Multimer to predict the structure of a ParT dimer to gain insight into its possible conformations (Fig. 4*A*). The top-ranking prediction from AlphaFold2 Multimer revealed a high-confidence dimerization interface at the N domain of ParT, mediated by helices α2 (pTM = 0.71, ipTM = 0.71), and a second dimerization interface at the C domain (pTM = 0.9, ipTM = 0.89) (Fig. 4*A* and *SI Appendix*, Fig. S8*B*). Consistent with the AlphaFold-Multimer-based prediction, bacterial two-hybrid assays demonstrated a strong self-interaction of the C domain of ParT, comparable to the well-established self-dimerization of a leucine zipper (ZIP) region of the yeast transcriptional activator GCN4 protein (*SI Appendix*, Fig. S8*C*). Similarly, helix α2 at the N domain of ParT also exhibited self-interaction, albeit notably weaker than the C domain (*SI Appendix*, Fig. S8*C*).

To further investigate whether the opposing ParT N domains dimerize in solution, we performed site-specific crosslinking of a purified ParT variant using the sulfhydryl-to-sulfhydryl crosslinker bis-maleimidoethane (BMOE) which covalently crosslinks cysteine residues within 8 Å of each other. Based on the AlphaFold2-Multimer-predicted structure, residue S48 at the N domain was selected and substituted by cysteine (Fig. 4*A*). The S48C substitution did not impact the loading and accumulation of ParT (S48C) on a closed DNA loop (*SI Appendix*, Fig. S9*A*). In a further control, ParT (WT), despite containing a native cysteine 81 residue, crosslinked minimally under tested conditions (*SI Appendix*, Fig. S9*B*). We observed that, in the absence of *parS* DNA, ~25% ParT (S48C) was crosslinked (lane 2, Fig. 4*B*), and the crosslinking efficiency did not change in the presence of a 40-bp scrambled *parS* DNA (lane 4, Fig. 4*B*). However, the crosslinking efficiency increased to ~52% (lane 3, Fig. 4*B* and *SI Appendix*, Fig. S9 *B* and *C*) when a cognate *parS* DNA was included. Furthermore, consistent with the lack of a CTPase domain, the addition of NTP had no effect on the crosslinking of ParT (S48C) (*SI Appendix*, Fig. S9*D*). To further confirm the specificity of cysteine crosslinking at position 48, we constructed and purified a ParT(A68C) variant, in which the opposing cysteines at position 68 were predicted to be 64 Å apart—too distant for the 8 Å BMOE crosslinker. As expected, this negative-control variant crosslinked minimally, regardless of the presence of additional *parS* DNA (*SI Appendix*, Fig. S9*B*). Taken together, data so far indicate that a cognate *parS* DNA promotes the self-dimerization of opposing ParT N domains.

Next, to examine the dimerization of opposing C domains of ParT, we constructed a ParT (Q271C) variant. Again, the symmetry-related Q271C residues from opposing ParT subunits were predicted to be crosslinkable based on the AlphaFold2-Multimer predicted structure of a ParT dimer (Fig. 4*A*). ParT (Q271C) showed ~37% crosslinking efficiency, however, the presence of *parS* DNA did not change the crosslinked fraction (Fig. 4*B*). Further supporting the primary role of C domain in dimerization, we created a chimeric ParT (ZIP CTD) variant in which the C domain was replaced by a leucine zipper from GCN4. This well-characterized 33-amino-acid coiled-coil is known to strongly self-dimerize. Similar to ParT (WT), the ParT (ZIP CTD) variant also could accumulate on a 180-bp *parS* DNA loop but not a BamHI/EcoRI-restricted 180-bp DNA with a free end (*SI Appendix*, Fig. S10). These findings suggest that the C domain likely serves as a constitutive dimerization domain, whereas the N domain could also self-dimerize but require *parS* DNA to facilitate this process.

### The Distance Between *parS* Half-Sites Is Important for ParT Accumulation but Not ParT Loading on DNA.

**Fig. 5.**
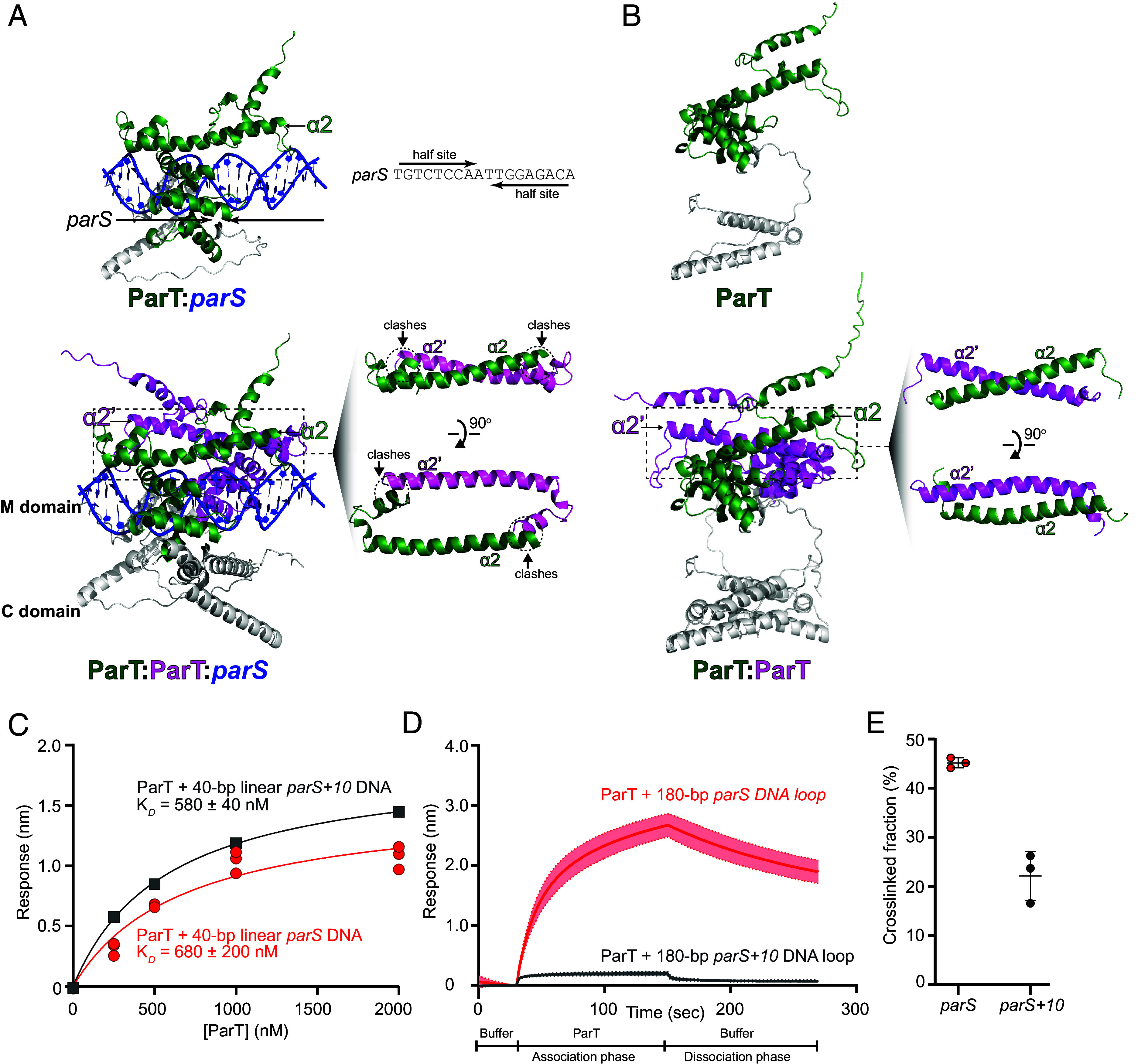
Each ParT subunit can accommodate a *parS* half site. However, when two opposing ParT subunits bind to *parS*, steric clashes are predicted to occur. (*A*) A structural model of AlphaFold2-predicted ParT bound to DNA. We superimposed two ParT chains onto *C. crescentus* ParB bound to opposing halves of its *parS* site (PDB: 6T1F), using the DNA-binding M domain to guide structural alignment. (*Top* panel) A model of a single ParT subunit bound at *parS*. An 18-bp *parS* is an inverted repeat, with each half site represented by an arrow. (*Bottom* panel) A model of ParT dimer bound at *parS* showed steric clashes between helix α2 and the first helix from the M domain of an opposing ParT subunit. (*B*) (*Top* panel) A model of a single ParT subunit. (*Bottom* panel) A model of a ParT dimer where opposing N-domain helices α2 and α2′ interact. (*C*–*E*) The distance between two *parS* half sites is important for ParT accumulation but not ParT loading on a DNA loop (*C*) *parS+10* DNA contains an additional 10 bp in between two *parS* half sites. BLI analysis of the interaction between increasing concentrations of ParT and 40-bp DNA duplexes harboring either a *parS* or a *parS+10* site. Experiments were performed in triplicates, and K_D_ values were estimated by curve-fitting. See also *SI Appendix*, Table S1 for K_D_ values for ParT-*parS/parS+10* interaction when SPR was used instead of BLI. (*D*) BLI analysis of the interaction between 1 µM ParT with a 180-bp dual biotin-labeled DNA that contains either a *parS* or a *parS+10* site. Mean and SD (shading) from three replicates are shown. (*E*) Quantification of the major BMOE-mediated crosslinked fraction of ParT (S48C) incubated with 40-bp *parS* or *parS+10* DNA duplexes.

To better understand how *parS* binding might promote subsequent self-dimerization of ParT N-domain, we generated a structural model of AlphaFold2-predicted ParT bound to DNA. We superimposed two ParT chains onto *C. crescentus* ParB bound to opposing halves of its *parS* site (PDB: 6T1F), using the DNA-binding M domain to guide structural alignment (Fig. 5*A*). Our analysis revealed that each ParT subunit can accommodate a *parS* half-site. However, when two opposing ParT subunits bind to *parS*, steric clashes occur (Fig. 5*A*). In contrast, when the ParT dimer was modeled without DNA, no steric clashes were observed at the N domain, and the two opposing helices α2 and α2′ dimerized together (Fig. 5*B*). Thus, we propose that *parS*-binding might stabilize an intermediate state where the clashes between two opposing N-domains (of a DNA-bound ParT dimer) are resolved, presumably through a conformational change in helix α2. This transient state might allow the dimerization of opposing helices α2 and α2′, coinciding with the escape of ParT from the *parS*-binding site.

The model here predicts that the steric clashes between opposing N-domains of the *parS*-bound ParT dimer, and their subsequent resolution, are essential for N-domain self-dimerization and the escape of ParT from *parS* site. To test this hypothesis, we eliminated the steric clashes by inserting 10-bp spacer (equivalent to a full DNA helical turn) between the two *parS* half sites to generate a *parS+10* DNA substrate. We found that ParT bound to a 40-bp *parS*+10 DNA substrate with a similar affinity to a wild-type *parS* DNA (Fig. 5*C*), indicating that the loading of ParT onto DNA substrates was not affected by the extra spacer. However, when a DNA loop was used (Fig. 5*D*), a low BLI signal was observed when 1 µM ParT was incubated with probes carrying a 180-bp dual-biotin *parS+10* DNA loop (Fig. 5*D*), indicating that ParT could no longer accumulate on such closed DNA loops. Consistently, the crosslinking efficiency of ParT (S48C) decreased to ~22% in the presence of 40-bp *parS+10* DNA duplexes compared to ~45% when a wild-type *parS* DNA was used (Fig. 5*E*). From these findings, we conclude that distancing *parS* half-sites eliminates steric clashes between opposing ParT subunits, thereby uncoupling ParT loading from ParT accumulation. Overall, these observations are consistent with the proposal that steric clashes between opposing ParT binding to each *parS* half-site are fundamental to the subsequent ParT N-domain self-dimerization, which enables ParT to escape from *parS* to the neighboring DNA.

### Disrupting the N-Domain Helix α2 Hinders ParT Accumulation but Not ParT Loading on DNA.

**Fig. 6.**
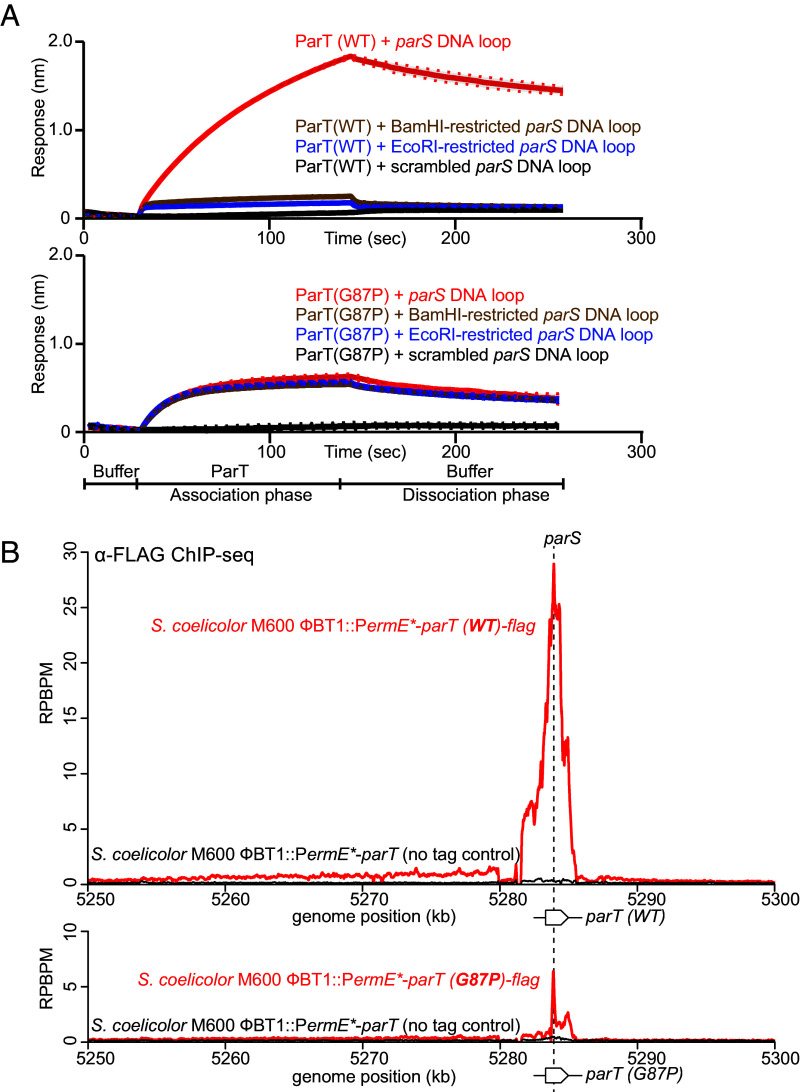
Substitution of glycine 87 on helix α2 by a proline hinders ParT accumulation but not ParT loading on DNA. (*A*) ParT (G87P) cannot accumulate on a closed *parS* DNA loop. BLI analysis of the interaction between 1 µM ParT (WT) (*Top* panel) or ParT (G87P) (bottom panel) with a 180-bp dual biotin-labeled DNA that contains either a *parS* or a scrambled *parS* site. Interactions between a dual biotinylated DNA and a streptavidin (SA)-coated probe created a closed DNA loop where both ends were blocked. 180-bp *parS* DNA loops were subsequently restricted by EcoRI or BamHI to generate an open free end. Mean and SD (shading) from three replicates are shown. See *SI Appendix*, Fig. S11*A* for the position of G87 residue on helix α2. (*B*) ParT (G87P) is defective in occupying DNA neighboring *parS*. α-FLAG ChIP-seq profiles show the enrichment of FLAG-tagged ParT (WT or G87P) at *parT* (WT/G87P) genes integrated at the ϕBT1 phage integration site on the chromosome of plasmid-less *S. coelicolor* M600. Profiles were plotted with the *x*-axis representing genomic positions and the *y*-axis representing the number of reads per base pair per million mapped reads (RPBPM). ChIP-seq experiments were performed twice using biological replicates, and a representative profile is shown.

To further test the model that *parS* binding stabilizes a ParT intermediate in which steric clashes in helices α2 and α2’ are rapidly resolved, we sought to find a mutation in helix α2 that allows the loading of ParT at *parS* but prevents its escape from *parS* and thus its accumulation on neighboring DNA. Fourteen amino acid substitutions, either to alanine or to proline, were introduced to α2 (*SI Appendix*, Fig. S11*A*). The purified ParT variants were assayed for binding to 40-bp linear *parS* or scrambled *parS* DNA, as well as for accumulation on a 180-bp *parS* DNA loop by BLI (*SI Appendix*, Fig. S11*B*). Among the 14 tested variants, ParT(G87P) was readily purified and bound both an intact 180-bp DNA loop and a BamHI /EcoRI-restricted 180-bp DNA to the same extent (Fig. 6*A*). In contrast, ParT (WT) exhibited ~five-fold higher response to an intact DNA loop compared to a restricted DNA (Fig. 6*A*). We reasoned that ParT (WT), after loading on *parS* site, diffuses to the neighboring DNA and slides off the free DNA end created by restriction enzyme digestion of the DNA loop. In contrast, ParT (G87P) presumably loaded onto *parS* but did not escape the *parS* site to diffuse onto neighboring DNA, resulting in no difference in BLI response regardless of whether an intact DNA loop or a restricted DNA loop was used.

To further corroborate that ParT(G87P) is loading-competent but sliding-defective, we expressed FLAG-tagged *parT (WT)* or *parT(G87P)* ectopically from the chromosome in a plasmid-free *S. coelicolor* strain and performed anti-FLAG ChIP-seq experiment. Note that there is a single *parS* site on the coding sequences of *parT* and *parT (G87P)* in these strains. Similar to data in Fig. 3*A*, the ChIP-seq profile of ParT (WT) showed a broad 5-kb peak with a summit at the *parS* site (Fig. 6*B*), consistent with ParT not only binding site-specifically at *parS* but also occupying neighboring DNA. In contrast, the ChIP-seq profile of ParT(G87P) was much narrower with a distinct summit at the *parS* site (Fig. 6*B*), consistent with the notion that ParT (G87P) can still be loaded at *parS* but is impaired in occupying neighboring DNA. We noted that the ParT (G87P) ChIP-seq peak submit is lower in height than that of ParT (WT), despite the equal protein levels in the cells as determined by anti-ParT immunoblotting (*SI Appendix*, Fig. S12). The reason for a lower occupancy specifically at *parS* is, however, not yet known.

The G87 residue is located in the middle of the helix α2 that mediates N-domain self-dimerization (*SI Appendix*, Fig. S11*A*); substitution by a proline at this position likely disrupts this key helix α2. Given the phenotype caused by the G87P substitution, we reason that disrupting helix α2 either i) prevents the steric clashes between opposing ParT N-domains at the *parS*-binding step, or ii) prevents the subsequent self-dimerization between α2 and α2′, thereby hindering escape from the *parS* site to neighboring DNA. Overall, these data support the existence of a molecular gate at ParT N-terminal domain (N-gate), whose closing is facilitated by *parS* and is dependent on helix α2.

## Discussion

### A Proposed Model for ParA-ParT-*parS* Complex Assembly.

**Fig. 7.**
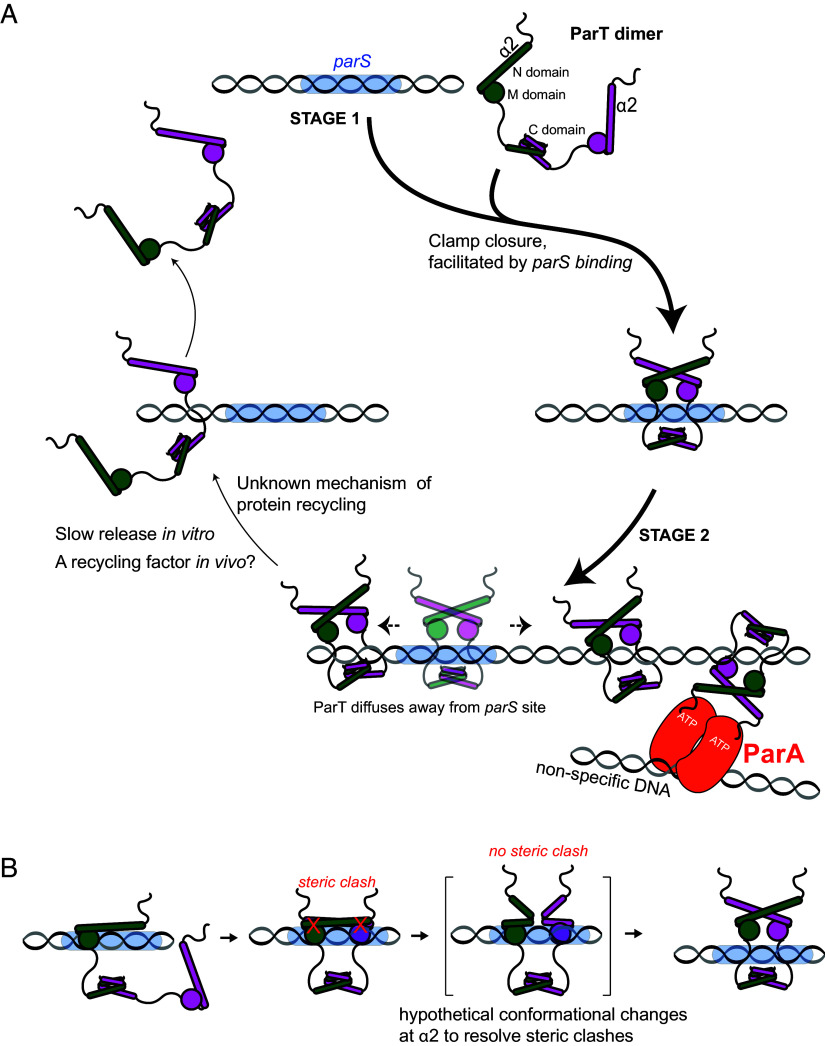
A proposed model for ParAT*S* complex assembly. (*A*) ParT has only a single gate at the N domain, while the ParT C domain functions as a constitutive dimerization domain. An open-clamp ParT, in which the M domain is accessible to DNA, can initially load onto *parS* DNA (cyan) (Stage I). Binding to *parS* facilitates the self-dimerization of the N-domain helix α2 from opposing ParT subunits, closing a clamp that enables ParT to escape to the neighboring DNA (see also panel B for further discussion on this stage). A now free *parS* site becomes available for the next ParT to load onto and subsequently diffuse away (Stage II). The repeating cycles of ParT loading and diffusion likely enable multiple ParT molecules to accumulate around the *parS* site, thereby creating a high local concentration of ParT to activate the ATPase activity of the partner protein, ParA (orange). The ParT clamp releases very slowly from the DNA in vitro (thin arrow). It is unknown whether ParT display higher turnover rate in vivo and whether a recycling factor might facilitate the release from DNA inside the cells. (*B*) A ParT subunit can bind each *parS* half-site individually but not simultaneously due to steric clashes (red crosses). Binding at *parS* potentially stabilizes a ParT intermediate where the steric clashes are rapidly resolved, likely through a conformational change in helices α2 and α2′. Notably, AlphaFold2 could not predict this rare conformational state, likely because it is unfavorable. In the ParT intermediate, the opposing helices α2 and α2′ dimerize because this conformational state is more favorable. Concomitantly, the opposing *parS*-binding domains (M) reorient and move closer together (as predicted by the AlphaFold2 model of ParT dimer; see also Fig. 5*B*), making them incompatible with binding two consecutive major grooves on the *parS* site. In this way, the ParT dimer escapes from a high-affinity *parS* binding site to the neighboring DNA.

In this work, we report an atypical DNA segregation protein, ParT, that self-loads onto DNA at a specific *parS* site to diffuse and accumulate on a DNA loop independently of NTP. Results from optical tweezers experiments show no evidence of a filament-like structure of ParT on DNA, indicating that ParT is unlikely to polymerize along the DNA. Furthermore, we did not observe ParT-mediated DNA condensation, suggesting that it does not bridge distal DNA together under the tested conditions. The observation of diffusive single ParT molecules in an optical tweezers experiment and the requirement of a closed DNA loop for ParT accumulation are more consistent with a model of ParT that diffuses/slides on DNA (Fig. 7).

Our data suggest that ParT has both open- and a closed-clamp conformations, enabling ParT i) to load site-specifically at *parS* (stage 1, Fig. 7*A*) and then ii) to escape a high-affinity *parS* site to diffuse/slide to neighboring DNA (stage 2, Fig. 7*A*), and iii) possibly to release from the DNA (Fig. 7*A*). AlphaFold2 Multimer predicted only the closed-clamp conformation, where the two opposing helices α2 self-dimerize to close the N-gate. Given that the self-interaction of helix α2, as suggested by bacterial two-hybrid assay (*SI Appendix*, Fig. S8*C*), is relatively weak, we propose the existence of an open-clamp conformation (where helix α2 is not self-interacting) in the cytoplasm before engaging with DNA (Fig. 7*A*). The open-clamp ParT can load site-specifically at *parS*. In this open conformation, the DNA-binding (M) domains from opposing ParT subunits are accessible to the *parS* site (Fig. 7*A*, stage I). The C domain of ParT could be substituted by a heterologous dimerization domain without impairing ParT functions (*SI Appendix*, Fig. S10), indicating that the C domain is constitutively dimerized. This suggests that the DNA can only access the M domain via the N-gate.

In stage 2, *parS*-bound ParT likely switches conformation to a closed clamp, allowing it to escape from the high-affinity binding site to diffuse to the neighboring DNA. In canonical ParB CTPases, it has been proposed that opposing ParB binding to each *parS* half-site sterically clash, forcing the CTPase (N) domain to untether from the DNA-binding (M) domain to now dimerize to the N-domain of the opposing ParB subunit (18). This self-dimerization of the N-domain, locked in placed by CTP, forms a closed-clamp conformation (15–18). In this state, the DNA-binding (M) domains of ParB reorient, preventing binding two consecutive major grooves of *parS* (15, 16). This CTP-dependent reorientation of the *parS* DNA-binding (M) domain enables ParB to escape from a high-affinity *parS* site to diffuse away to the adjacent DNA (13, 15, 17, 29). For canonical ParB, *parS*-binding selects and stabilizes the N-M-untethered ParB intermediate, facilitating its subsequent conversion to a closed-clamp conformation (18). Thus, *parS* serves essentially as a catalyst to accelerate the repeated conversion of *parS*-bound ParB to a closed-clamp ParB (18). In CTPase-lacking ParT, the formation of a closed clamp might just be facilitated by *parS* binding without the need of nucleotide binding. Modeling of DNA-bound ParT shows that each ParT subunit can accommodate a *parS* half site, but two opposing ParT clash at helices α2 and α2’ when bound to *parS* (Fig. 5*A*). We propose the presence of an intermediate state where these clashes are resolved, akin to the N-M-untethered ParB intermediate (18), allowing α2 and α2’ to then properly self-dimerize to close the N-gate (Fig. 7*B*). The role of *parS* here is to stabilize such ParT intermediate, to facilitate N-gate closure, a process that might be energetically favored but kinetically hindered (Fig. 7*B*). Supporting this role of *parS*, ParT (S48C) crosslinked twice more efficiently even in substoichiometric concentration of this DNA (*SI Appendix*, Fig. S9*C*). Additionally, when the distance between the two *parS* half sites was increased by a helical turn to eliminate clashes between the opposing helices α2 (Fig. 5 *C*–*E*), or when a proline substitution disrupted helix α2 (Fig. 6 and *SI Appendix*, Fig. S11*A*), N-domain self-dimerization and the ability to accumulate onto the neighboring DNA was notably reduced. Collectively, these data suggest a possible model for ParT clamp closure involving a steric clash when each ParT subunit binds to each *parS* half-site (Fig. 7*B*). A closed-clamp ParT escaping from the loading site to the neighboring DNA also vacates *parS* for the next ParT loading event. The repeated ParT loading on *parS*, followed by escape and diffusion to the neighboring DNA, results in multiple ParT molecules decorating the vicinity of *parS*.

The overall behavior of the ParT dimer is reminiscent of the ParB CTP-dependent DNA-sliding clamp (32, 33). A molecular switch, such as the canonical ParB, typically requires a nucleotide cofactor like CTP, as stable switching involves a transition from a high-energy to a low-energy conformational state, which is reversed through nucleotide hydrolysis. In contrast, ParT accumulates nearby to *parS* DNA in vitro seemingly without a nucleotide cofactor. The closed-clamp conformation can be explained by the achievement of a lower-energy state upon *parS* binding. However, how this state is reversed without the nucleotide cofactor hydrolysis remains unclear. We indeed observed that accumulated ParT on a DNA loop were very slowly released (~3.5-fold slower than that of *C. crescentus* ParB) (16) when the BLI probe was returned to a buffer-only solution (Fig. 3*D*, dissociation phase). It is not yet clear whether ParT displays higher turnover rate in vivo and whether a recycling factor (either a dedicated one or collisions with nucleoid-associated proteins or RNA/DNA polymerases) might facilitate the release from DNA inside the cells (Fig. 7, stage 3). Future in vivo works are necessary to resolve the question of the energetics of the nucleotide-free ParT local accumulation system.

### A High Local Concentration of ParT Activates the ATPase Activity of ParA.

The accumulation of ParT around *parS* results in a high local concentration that is essential for the activation of the ATPase activity of its partner ParA protein (Fig. 2*E*). These are essential features, that are shared with type-I ParAB*S* systems, to ensure the no return zone only forms where the ParAB*S* segrosome is. It would be detrimental to the DNA segregation process if ParB was to activate the ATPase activity of ParA at a low concentration because cytoplasmic DNA-unbound ParB would unproductively release ParA from the nucleoid at spatially random locations inside the cells. In a bigger picture, it has been noted that, despite the diversity of evolutionarily distinct DNA partition systems (type I, II, and III) with vastly different types of centromere-binding proteins (CBPs) and associated NTPases (1), there is a common feature in all systems: the requirement for a high local concentration of CBPs decorating the centromere-like *parS* region. The high local concentration of CBPs can either be functionally achieved by diffusion or oligomerization of CBP from a single *parS* site or by site-specific binding to an array of *parS* sites adjacent to each other (reviewed in ref. 1). In this context, CTP-independent ParT diffusion to accumulate on DNA represents another case of biological innovation to segregate bacterial DNA. Last, we note that, while both ParT and canonical ParB employ a positively charged N-terminal peptide to interact with their cognate ParA partners, the amino acid identity of their ParA-interacting motifs are distinct (ParT: SRR motif vs. ParB: LG-R/K-GL motif). This might contribute to ensuring the SCP2 ParAT*S* segrosome does not cross-interact, thus interfering with the several coexisting ParAB*S* systems in the same cell such as the single ParAB*S* system on the linear chromosome and two other ParAB*S* systems on the linear plasmid SCP1 (34–37).

## Materials and Methods

For complete Materials and Methods, see the *SI Appendix, Materials and Methods*.

### Plasmids and Strains.

All strains, plasmids, and oligonucleotides used in this study are listed in *SI Appendix*, Tables S3–S6. Details on constructions of plasmids and strains are in the *SI Appendix*.

### Measurement of ParA-ParT interaction by BLI assay.

Streptavidin (SA)-coated probes (Sartorius; cat# 18-5136) were first hydrated for 10 min in a binding buffer containing 100 mM Tris-HCl at pH 8, 150 mM sodium chloride, 1 mM magnesium chloride, and 0.005% (v/v) Tween-20. Biotinylated ParT was diluted in the binding buffer to the final concentration of 1 µM so that they could be immobilized onto the surface of the SA probe. Briefly, SA probes were attached to the BLI instrument (Fortebio) and incubated with shaking at 2,200 rpm sequentially in binding buffer for 30 s, then in 1 µM ParT solution in binding buffer for 120 s, and last in binding buffer for 120 s. To remove traces of nonspecifically bound ParT, probes were incubated for another 5 min in a high-salt buffer containing 100 mM Tris-HCl at pH 8, 1 M sodium chloride, 5 mM EDTA, and 0.005% (v/v) Tween-20, followed by a 10 min incubation in binding buffer.

To assay for ParA-ParT binding, ParA-His_6_ was first diluted in binding buffer to the final concentration of 1 µM, and preincubated on ice for 20 min with 1 mM ATP, or 0.05 mg/mL of salmon sperm DNA (Merck; cat# D1626), or combinations thereof. Afterward, ParT-coated probes were incubated in binding buffer for 30 s, with shaking at 2,200 rpm on the BLI instrument, to establish the baseline. The probes were subsequently transferred to a solution containing apo-ParA, ParA + ATP, or ParA + ATP + DNA and incubated with shaking for 120 s. Last, the probe was transferred to the binding buffer only and incubated for another 120 s. All assays were performed in triplicates, and the probe was regenerated in between each replicate by a 5-min incubation in high-salt buffer.

### Assessing ParT N-Terminus and C-Terminus Dimerization by In Vitro BMOE Crosslinking Assay.

ParT (S48C), ParT (Q271C), ParT (A68C), or ParT (WT), at 4 µM dimer concentration, was incubated on ice either alone or with 4 μM of 40-bp *parS* duplex or scrambled *parS* duplex in a crosslinking buffer (100 mM Tris-HCl pH 7.4, 150 mM sodium chloride, 5 mM magnesium chloride) for 5 min. To determine the crosslinking efficiency of ParT (WT/variants) in the presence of nucleotide triphosphates, the crosslinking reactions were supplemented with either 1 mM of ATP (Thermo Fisher Scientific; cat# R0441), CTP (Thermo Fisher Scientific; cat# R0451), GTP (Thermo Fisher Scientific; cat# R0461), or UTP (Thermo Fisher Scientific; cat# R0471). Afterward, 20 mM DMSO solution of bismaleimidoethane (ThermoFisher; cat# 22323) was added to the reaction mixture to the final concentration of 2 mM, and the mixture was incubated at room temperature for 5 min before the crosslinking reactions were quenched by adding SDS-PAGE loading dye containing β-mercaptoethanol. Quenched samples were heated to 96 °C for 3 min to denature the protein, cooled down, and loaded on 12% Tris-glycine polyacrylamide denaturing gels (ThermoFisher; cat# XP00122BOX). Crosslinked and noncrosslinked species were then resolved by electrophoresis at 150 V for 55 min. Upon completion of electrophoresis, gels were stained using an InstantBlue Coomassie solution (Abcam; cat# ab119211), and band intensity was quantified using ImageJ (NIH) (38). All experiments were performed in triplicates.

### ChIP-seq and Data Analysis.

*S. coelicolor* A3(2) or *S. coelicolor* M600 strains harboring a FLAG-tagged or nontagged *parT (WT/variants)* alleles under the control of an ermE* promoter were grown as liquid cultures for ChIP-seq. ChIP-seq was performed as described previously (16), and a detailed protocol for ChIP-seq and data analysis are reported in the *SI Appendix*.

### Confocal Optical Tweezers Experiments.

Confocal-optical tweezers experiments were carried out using a dual optical tweezers setup combined with confocal microscopy and microfluidics (C-Trap; Lumicks), essentially as described previously (30). A detailed protocol is reported in the *SI Appendix*.

## Supplementary Material

Appendix 01 (PDF)

Dataset S01 (PDF)

Dataset S02 (XLSX)

Dataset S03 (XLSX)

## Data Availability

ChIP-seq data have been deposited in Gene Expression Omnibus (ChIP-seq data are available at GEO (Accession code: GSE263222) (39). All other data are included in the manuscript and/or supporting information.
