# Supplementary material for: Expanding the diversity of bacterial DNA partitioning: A CTP-independent ParABS system for plasmid partitioning in Streptomyces
Source: Proc Natl Acad Sci U S A. 2025 Jul 2;122(27):e2406398122. doi: 10.1073/pnas.2406398122 (PMC12260392; doi:10.1073/pnas.2406398122)
Supplement: Supplementary file 1 — Appendix 01 (PDF) [file pnas.2406398122.sapp.pdf]

Supplementary Information for

Expanding the diversity of bacterial DNA partitioning: A CTP-independent ParAB*S* system for plasmid partitioning in *Streptomyces*

Kirill V. Sukhoverkov[1], Francisco Balaguer-Perez[2], Clara Aicart-Ramos[2], Ngat T. Tran[1], Abbas Maqbool[3], Martin Rejzek[1], Govind Chandra[1], Fernando Moreno-Herrero[2], Tung B. K. Le[1*]*

[1] Department of Molecular Microbiology
John Innes Centre, Norwich, NR4 7UH, United Kingdom
[2] Department of Macromolecular Structures
Centro Nacional de Biotecnología, Consejo Superior de Investigaciones Científicas, Madrid, Spain
[3] Department of Biochemistry and Metabolism
John Innes Centre, Norwich, NR4 7UH, United Kingdom

* corresponding authors

Tung B. K. Le: tung.le@jic.ac.uk

Fernando Moreno-Herrero: fernando.moreno@cnb.csic.es

The PDF file includes:

Supplementary Materials and Methods

Figs. S1 to S12

Tables S1 to S6

SUPPORTING TEXT

MATERIALS AND METHODS

Construction of plasmids and strains

Construction of pET21b::parT-(his)$_6$ (WT and mutants) and pET21b::parA-(his)$_6$ (WT)
Two overlapping DNA fragments *ParT_WT_f1* and *ParT_WT_f2* containing an N-terminal or a C-terminal half of a codon-optimized version of *parT* (SCP2.04c), respectively, were chemically synthesized (gBlocks, IDT). The two gBlocks fragments were assembled into an NdeI-HindIII-cut pET21b backbone using a 2x NEB Gibson assembly master mix (NEB; cat# E2611). To enable such assembly, pET21b was digested with NdeI (NEB, cat# R0111S) and HindIII-HF (NEB, cat# R3104S) and purified by gel extraction. A 10 µL reaction mixture was created with 5 µL 2x Gibson master mix and 5 µL of combined equimolar concentration of purified backbone and gBlocks fragments. This mixture was incubated at 50°C for 60 min. Gibson assembly was possible owing to a 37-bp sequence shared between the NdeI-HindIII-cut pET21b backbone (**Table S3**) and each of the two gBlocks fragments. The mixture was introduced into *E. coli* DH5α (**Table S4**) by heat-shock transformation and carbenicillin-resistant colonies were selected. Subsequently, plasmids were isolated and verified either by Sanger sequencing (Genewiz, UK) or by whole-plasmid sequencing (Plasmidsaurus). A correct plasmid was introduced into *E. coli* Rosetta (DE3) pLys (**Table S4**) to create an overexpression strain. The overexpression plasmids for ParT (variants)-His$_6$ were constructed similarly with the intended mutations introduced into either *f1* or *f2* gBlocks fragment at the designing step.
An overexpression plasmid for ParA-His$_6$ was also constructed similarly but using *ParA_WT_f1* + *ParA_WT_f2* pair of gBlocks fragments.

Introducing a FLAG-tagged parT allele to Streptomyces coelicolor A3(2) genome
To introduce a FLAG tag to the C-terminus of *parT*, DNA containing *parT* was amplified by PCR from *S. coelicolor* A3(2) genomic DNA using primers *ParT_CFLAG_Fw* and *ParT_CFLAG_Rv* (**Table S5**), and subsequently purified by gel extraction. The purified PCR product was assembled with an NdeI-HindIII-cut pIJ10257 backbone using a 2x Gibson master mix (NEB). The resulting plasmids were verified by Sanger sequencing (Genewiz, UK). A plasmid carrying a non-tagged version of *parT* was constructed similarly, but primers *ParT_Fw* and *ParT_Rv* (**Table S5**) were used instead.
To integrate *parT*-FLAG or the non-tagged version onto the *S. coelicolor* A3(2) ΦBT1 phage integration site, *E. coli* ET12567 + pUZ8002 (**Table S4**) were transformed with plasmid pIJ10257::*parT-flag* or pIJ10257::*parT* (**Table S3**), and subsequently conjugated to *S. coelicolor* A3(2) (**Table S4**) as previously described(1). Conjugants were purified by re-streaking to single colonies twice on SFM medium containing 50 µg/mL of hygromycin (Merck, cat# 10843555001). Spore stocks of resulting strains were eventually prepared and stored at -80°C.

Introducing mCherry-tagged parT allele to S. coelicolor A3(2) and S. coelicolor M600
To create a C-terminal mCherry-tagged *parT* allele, DNA containing *parT* was first amplified by PCR from *S. coelicolor* A3(2) genomic DNA as two fragments using the primer pairs *ParT_CCherry_Fw* + *ParT_par_disr_Rv* and *ParT_par_disr_Fw* + *ParT_CCherry_Rv* (**Table S5**) and subsequently purified by gel extraction. The oligos were designed so that the resulting *parT* allele, after Gibson assembly, would not contain an internal *parS* site but the coded amino acid sequence remains unchanged. The purified DNA fragments were then assembled with an NdeI-XhoI-digested pSS88 backbone using a 2x Gibson mastermix (NEB). The resulting plasmids were verified by Sanger sequencing (Genewiz, UK).

To integrate *parT-mcherry* allele into *S. coelicolor* A3(2) chromosome at the ΦBT1 phage integration site, *E. coli* ET12567 + pUZ8002 (**Table S4**) were transformed with plasmid pSS88::*parT-mcherry* (**Table S3**), and subsequently conjugated to *S. coelicolor* A3(2) or *S. coelicolor* M600 (**Table S4**) as previously described(1). Conjugants were purified by re-streaking to single colonies twice on SFM

medium containing 50 µg/mL of hygromycin (Merck, cat# 10843555001). Spore stocks of resulting strains were eventually prepared and stored at -80°C.

Construction of bacterial two-hybrid plasmids

DNA encoding the N-domain helix α2 or the C-domain of ParT were chemically synthesized as double-stranded gBlocks DNA fragments (IDT). These DNA fragments were assembled into a BamHI-EcoRI-cut pKT25 or a BamHI-EcoRI-cut pUT18C (**Table S3**) using a 2x Gibson master mix (NEB). Gibson assembly was possible due to a 23 bp sequence shared between the gBlocks DNA fragment and restricted pKT25 /pUT18C plasmids. These 23 bp regions were incorporated during the gBlocks design. The resulting plasmid was sequence verified by whole-plasmid sequencing (Plasmidsaurus).

Construction of bacterial two-hybrid strains

BTH101 *E. coli cya-* cells were made chemically competent and were doubly transformed with combinations of pKT25 and pUT18C plasmids (**Table S4**). Carbenicillin- and kanamycin-resistant colonies were subsequently selected.

Microscopy imaging of mCherry-tagged ParT in *S. coelicolor* A3(2) and M600

To image fluorescently labeled ParT *in vivo*, a Zeiss Axio Observer Z.1 inverted epifluorescence microscope fitted with an sCMOS camera (Hamamatsu Orca FLASH 4), a Zeiss Colibri 7LED light source, a Hamamatsu Orca Flash 4.0v3 sCMOS camera and a temperature-controlled incubation chamber was used. Images were acquired using a Zeiss Alpha Plan-Apo ×100/1.46 Oil DIC M27 objective with mCherry excitation/emission wavelength of 587 and 610 nm respectively. Still images were collected using Zen Blue (Zeiss).

To generate a vegetative hyphae sample, spores of *S. coelicolor* A3(2) ΦBT1::P*ermE*-parT-mcherry* and *S. coelicolor* M600 ΦBT1::P*ermE*-parT-mcherry* were spotted onto solid SFM agar (supplemented with 50 µg/mL of hygromycin) next to a microscope coverslip that had been inserted into the agar at approximately a 45° angle. Plates were incubated at 30 °C for five days. At the end of the incubation, glass coverslips with the climbed-up hyphae were pulled out from the SFM agar, then mounted onto slides affixed with an 1% agarose pad for imaging.

Overexpression and purification of ParT (WT/variants)-His$_6$ and ParA-His$_6$

The C-terminally His-tagged ParT (WT and variants) were expressed from the plasmid pET21b in *E. coli* Rosetta (DE3) pLys cells (Merck, UK) (**Table S4**). Overnight culture (80 mL) was used to inoculate 4 L of LB broth supplemented with carbenicillin and chloramphenicol. Cells were grown at 37°C with shaking at 200 rpm till OD_{600nm} reached ~0.4, then cultures were cooled down to 16°C before isopropyl-β-D-thiogalactopyranoside was added to the final concentration of 0.5 mM. The culture was incubated overnight at 16°C with shaking at 200 rpm before the cells were collected by centrifugation at 5400 g at 16°C.

Pelleted cells were resuspended in 50 mL of buffer containing 100 mM tris(hydroxymethyl)aminomethane hydrochloride (Tris-HCl) pH 7.4, 300 mM sodium chloride, 10 mM imidazole and 5% (v/v) glycerol (buffer A), supplemented with 1 µL of Benzonase (Merck; cat# E1014), 5 mg of lysozyme (Merck; cat# 4403), and an EDTA-free protease inhibitor tablet (Merck; cat# 11873580001). The cell suspension was incubated on a wheel rotator at room temperature for 45 min before cell lysis by sonification (10 cycles of 15 s on/off on ice at an amplitude of 20 microns).

The cell debris was pelleted by centrifugation at 42000 g for 40 min at 4°C. The supernatant was filtered through a 0.22 µm sterile filter (Starlab; cat# E4780-1226) and loaded onto a 1 mL His-Trap column (Cytiva; cat# 17524701) that had been pre-equilibrated with buffer A (100 mM Tris-HCl pH 7.4, 300 mM sodium chloride, 10 mM imidazole, and 5% (v/v) glycerol). The column was washed with buffer A until A_{280nm} plateaued. ParT was eluted from the column by an increasing gradient of buffer B (100 mM Tris-HCl pH 7.4, 300 mM sodium chloride, 500 mM imidazole, and

5% (v/v) glycerol). ParT-containing fractions were pooled and concentrated using Amicon Ultra-15 centrifugal filter units (Merck; cat# UFC901024), and buffer exchanged to a low-salt buffer (100 mM Tris-HCl pH 8, 25 mM sodium chloride and 10% (v/v) glycerol) using PD-10 desalting columns (Cytiva; cat# 17085101).

The low-salt ParT solution was loaded onto a 1 mL Heparin HP column (Cytiva; cat# 17040601) that had been pre-equilibrated with the low-salt buffer. The column was then washed with the low-salt buffer until A_{280nm} plateaued. ParT was eluted from the column by an increasing gradient of a high-salt buffer (100 mM Tris-HCl pH 7.4, 1 M sodium chloride, 10% (v/v) glycerol). ParT-containing fractions were pooled, concentrated using Amicon Ultra-15 centrifugal filter units, and buffer exchanged to an EDTA-containing buffer (100 mM Tris-HCl pH 8, 250 mM sodium chloride, 10 mM EDTA and 10% (v/v) glycerol) using PD-10 desalting columns. Afterward, to remove traces of non-specific DNA, metal ions, or nucleotide triphosphates, the ParT solution in EDTA-containing buffer was concentrated to ~0.5 mL and dialyzed overnight against 2 L of the EDTA-containing buffer using a dialysis tubing with a molecular weight cut-off of 10 kDa (Thermo Fisher Scientific; cat# 68100). The dialyzed protein was centrifuged at 17000 g at 4°C to remove the precipitated protein, then the supernatant was loaded onto a gel-filtration HiLoad 16/600 Superdex 200pg column (Cytiva; cat# 28989335) pre-equilibrated with a buffer containing 10 mM Tris-HCl pH 8.0 and 250 mM sodium chloride. Eluted fractions corresponding to non-aggregated ParT were pooled, concentrated, and mixed with glycerol to the final concentration of 5% (v/v). The protein was then aliquoted, flash-frozen in liquid nitrogen, and stored at -80°C.

The ParT (S48C)-His_6, ParT (Q271C)-His_6, ParT (A68C)-His_6 and ParA-His_6 were prepared following a similar procedure, however, the purification on heparin columns was omitted. No contaminant was observed by SDS-PAGE (**Fig. S1B**). Additionally, to ensure that cysteine residues were reduced, ParT (S48C)-His_6, ParT (Q271C)-His_6, and ParT (A68C)-His_6 aliquots were supplemented with 1 mM tris (2-carboxyethyl) phosphine hydrochloride (Merck; cat# C4706) before flash-freezing in liquid nitrogen. Other ParT variants (**Fig. S11**) were purified using Ni-affinity chromatography only.

Biotinylation of ParT

NHS-PEG_4-Biotin (Thermo Scientific; cat# A39259) reagent was used to biotinylate ParT-His_6 for bio-layer interferometry (BLI) assays. A 10 µL of NHS-PEG_4-Biotin (stock: 40 mM, freshly dissolved in DMSO) was added to 0.5 mL of 40 µM ParT solution in a reaction buffer containing 100 mM HEPES pH 7.4, 300 mM sodium chloride, and 10% (v/v) glycerol. The solution was mixed by inverting the tube several times and incubated at room temperature for 30 min. Then, to remove residual unincorporated biotinylating reagent, the reaction mixture was buffer exchanged to a storage buffer containing 100 mM Tris-HCl pH 7.4, 300 mM sodium chloride, and 10% (v/v) glycerol using PD-10 desalting columns. Afterward, the protein solution was concentrated to the final concentration of ~60 µM using Amicon Ultra-15 centrifugal filter units, aliquoted, flash-frozen in liquid nitrogen, and stored at -80°C. The same protocol was used to prepare the biotinylated ParTΔN3 variant.

Preparation of biotinylated linear *parS* DNA

A pair of 40-bp single-stranded DNA oligonucleotides: a 5´-biotinylated *parS_Fw* and a non-biotinylated *parS_Rv* (**Table S5**) were dissolved in annealing buffer (1 mM Tris-HCl pH 8.0, 5 mM sodium chloride) to the final concentration of 100 µM. An equal volume of each oligonucleotide solution was mixed and heated at 98°C for 5 min before being left to cool down to room temperature overnight to form 50 µM double-stranded biotinylated *parS* duplex. A biotinylated scrambled *parS* duplex was also prepared similarly but using a 5´-biotinylated *scrambled parS_Fw* and a non-biotinylated *scrambled parS_Rv* oligonucleotides instead (**Table S5**).

Preparation of double-biotinylated DNA substrate

A DNA construct containing BamHI and EcoRI restriction sites, a *parS* sequence, a *tetO* sequence, *M13For* and *M13Rev* homologous regions at each end was chemically synthesized (gBlocks, IDT). To generate a dual biotin-labeled DNA substrate, PCR reactions were performed using a 2x GoTaq PCR master mix (Promega; cat# M7122), a 5´-biotinylated *M13For* and a 5´-biotinylated *M13Rev*

primers (**Table S5**), and the gBlock fragment as a template. The PCR product from eight reactions was pooled together and purified by agarose gel extraction.

Measurement of ParT NTPase activity by EnzChek phosphate release assay
NTP hydrolysis by ParT was monitored by following a similar procedure used for the measurement of ParA ATPase activity. ParT-His_6 was diluted to the final concentration of 10 µM in either a reaction buffer or in a reaction buffer supplemented with 10 µM of 40-bp *parS* duplex, and incubated on ice for 10 min. Afterward, 10 µL of ParT or ParT-*parS* mixture was mixed with 80 µL of the assay working solution in separate wells of a 96-well plate and incubated for 5 min at room temperature. Then, an NTPase reaction was initiated by adding 10 µL of 10 mM ATP, CTP, GTP, or UTP to wells containing the ParT-*parS* mixture in the working solution and the hydrolysis kinetics was monitored using BioTek Eon plate reader. The positive control Noc-His_6 was assayed similarly, but only CTPase activity in the presence of an equimolar amount of 22-bp *NBS* duplex was monitored.

Measurement of ParA ATPase activity by EnzChek phosphate release assay
ATP hydrolysis was monitored using an EnzCheck phosphate assay kit (Thermo Fisher Scientific; cat# E6646). ParA, ParT, and salmon-sperm DNA were mixed and pre-incubated on ice for 10 min at the following final concentrations of 5 µM, 5-150 µM, and 2 mg/mL, respectively, in a reaction buffer containing 100 mM Tris-HCl pH 7.4, 150 mM sodium chloride, and 5 mM magnesium chloride. In parallel, an NTPase assay working solution was prepared by diluting freshly defrosted aliquots of aqueous 1 mM 2-amino-6-mercapto-7-methylpurine riboside and 100 IU/mL of purine nucleoside phosphorylase in the reaction buffer to the final concentrations of 0.2 mM and 1 IU/mL, respectively. Afterward, 10 µL of the ParA-ParT-DNA mixtures were mixed with 80 µL of the assay working solution in separate wells of a 96-well plate (Cellstar; cat# 655180) and incubated for 5 min at room temperature. During this incubation, a fresh working solution of ATP was prepared by diluting 100 mM ATP (Thermo Fisher Scientific; cat# R0441) to 10 mM in the reaction buffer. ATP hydrolysis reactions were initiated by adding 10 µL of 10 mM ATP to the wells containing ParA, ParT, DNA in the working solution. Then, the plates were incubated at 25°C with constant shaking and the A_{360nm} was monitored using BioTek Eon plate reader (Agilent). Samples containing only ParA, ParT, or DNA were monitored similarly and served as controls. To convert A_{360nm} values to concentrations of inorganic phosphate, a calibration curve was generated using the same working solution. Briefly, potassium dihydrogen phosphate was added to the final concentration of 5 to 150 µM into wells containing the working solution, and A_{360nm} was monitored as described above. The results were analyzed using Excel and plotted in GraphPad Prism 9.

Measurement of protein-DNA interaction by BLI assay
A modified version of a BLI-based ParA-ParT binding assay was used to assay ParT-DNA interaction. Briefly, 40-bp biotinylated *parS* duplexes were immobilized on streptavidin-coated probes as described above, and then ParT-*parS* binding was measured in increasing ParT concentration from 0.25 to 2 µM, in three technical replicates per concentration. A 40-bp biotinylated scrambled *parS* duplex was also assayed in the same condition to assess non-specific DNA binding by ParT. To calculate the binding constants (K_D), the maximal values of BLI signal in the association phase were plotted as a function of ParT concentration. The resulting curve was approximated using GraphPad Prism 10 non-linear regression model “One site - Specific binding” with the default parameters using the following equation:

$$response\ (nm) = \frac{B_{max} \cdot ParT\ (\mu M)}{K_d + ParT\ (\mu M)}$$

Where *response (nm)* is the maximal values of BLI signal in the association phase measured in nm, *ParT (µM)* is ParT concentration measured in µM, K_d is an apparent ParT-*parS* dissociation constant measured in µM, and B_{max} is the estimated maximum specific binding possible under the given conditions, measured in nm.

Measurement of ParT-DNA loop interaction by BLI assay

A double biotinylated 180-bp DNA was diluted in binding buffer (100 mM Tris-HCl at pH 8, 150 mM sodium chloride, 1 mM magnesium chloride, and 0.005% (v/v) Tween-20) to the final concentration of 1 µM and immobilized on streptavidin-coated probes by sequential incubation with shaking at 2200 rpm in binding buffer for 30 s, then in the DNA substrate solution for 120 s, and finally in binding buffer for 120 s. To remove the traces of non-specifically bound DNA, the probes were incubated for another 5 min in a high-salt buffer (100 mM Tris-HCl at pH 8, 1 M sodium chloride, 5 mM EDTA, and 0.005% (v/v) Tween-20), followed by a 10 min incubation in binding buffer.

To measure ParT (WT/variants) binding onto the 180-bp DNA loop, DNA-coated probes were incubated in binding buffer for 30 s, with shaking at 2200 rpm on the BLI instrument, to establish the baseline and subsequently transferred to a solution containing 1 µM ParT in binding buffer and incubated with shaking for 120 s. Lastly, the probes were transferred to binding buffer and incubated for another 120 s. The assay was repeated three times, each time using a new probe.

To generate an open end on the left or right hand side of the 180-bp DNA loop, the tips of DNA-coated probes were immersed in 1x rCutSmart buffer (NEB; cat# B6004S) containing either 400 U/mL of BamHI-HF (NEB; cat# R3136S) or EcoRI-HF (NEB; cat# R3101S) and incubated for 2.5 hrs at 37°C. A control probe was also incubated in 1x rCutSmart buffer only to account for any possible non-enzymatic DNA degradation. For each digestion, three DNA-coated probes were used.

Surface Plasmon Resonance (SPR)

Single stranded DNA fragments (Sigma Aldrich) were annealed to 50 µM concentration in water as described previously (2). Experiments were performed at 4°C using a Biacore 8K (Cytiva) and the running buffer (10 mM HEPES pH 7.5, 200 mM NaCl, and 0.05% (v/v) Tween20). All experiments were conducted using previously published ReDCaT method and Series S Sensor Chip streptavidin (SA) from Cytiva (2). The SA chip has 16 flow cells. Flow cells 1, 3, 5, 7, 9, 11, 13, 15 were used a reference (FCref) and flow cells 2, 4, 6, 8, 10, 12, 14, 16 were used as test flow cells (FCtest). A multi-cycle kinetics approach was used to study interaction between different DNA fragments (*parS*, scrambled *parS*, or *parS+10*) and ParT protein. For each cycle, DNA fragments were injected over FCtest at a flow rate of 10 µl min^{-1} for 60 sec to gain a response between 50 and 60 RU. The protein was then flowed over both FCref and FCtest for 60 sec, followed by a dissociation time of 420 sec. The ReDCaT chip was then regenerated (stripping of captured DNA by denaturation) using buffer containing 1.0 M NaCl and 50 mM NaOH for 60 sec. Different concentrations of protein (ranging from 150 to 1875 nM) and buffer only controls were injected over both FCref and FCtest. The background response from FCref (nonspecific binding of the protein to the chip) was subtracted from the response from FCtest (binding of protein to DNA fragments). The inclusion of buffer-only controls enabled the use of double referencing whereby for each analyte measurement, in addition to subtracting the response in FCref from the response in FCref, a further buffer-only subtraction was made to correct for bulk refractive index changes or machine effects (3). The resulting sensorgrams were analysed using the Biacore Insight Evaluation Software (GE Healthcare). For kinetics analysis, a 1:1 binding model was applied to the data. The Rmax was fitted locally to reflect the regeneration of the chip and subsequent recapture of DNA and protein binding between cycles. SPR data were exported and plotted using Microsoft Excel. Each experiment was repeated a minimum of three times, with similar results.

Table S1. K_D values of ParT-DNA interactions. K_D values were obtained using different techniques: (1) biolayer interferometry with equilibrium analysis and (2) surface plasmon resonance with kinetics analysis. K_D values varied by less than five-fold across two different methodologies.

K_D (nM)	Determined by biolayer interferometry	Determined by surface plasmon resonance
ParT + *parS* DNA	680 ± 200 nM	150 ± 16 nM

ParT + scrambled *parS* DNA	no binding	no binding
ParT + *parS+10* DNA	580 ± 40 nM	730 ± 60 nM

Chromatin immunoprecipitation with deep sequencing (ChIP-seq) and data analysis
S. coelicolor A3(2) or *S. coelicolor* M600 strains harboring a FLAG-tagged or non-tagged *parT (WT/variants)* alleles under the control of an *ermE** promoter (**Table S4**) were grown as liquid cultures as follows. First, the spore stocks were diluted to the final $OD_{450\ nm}$ of 0.25 in 5 mL of 0.05M TES buffer and were incubated standing at 50°C for 10 min before adding 5 mL of 2x PG medium (1% (w/v) of yeast extract, 1% (w/v) of casamino acids and 10 mM of calcium chloride). Then, the heat-shocked spores suspensions were incubated with shaking at 37°C for 3 hours and subsequently pelleted by centrifugation at 3000 rpm for 10 min at room temperature. The pelleted spores were resuspended in 0.5 mL of growth media comprising 40% (v/v) of YEME and 60% (v/v) TSB and then transferred to a 50 mL of the same media in a conical flask where a metal spring was fit in the bottom to facilitate a disperse growth of *Streptomyces* mycelium. The cultures were then grown overnight at 30°C with shaking. Formaldehyde (Merck; cat# F8775) was added to the cultures to the final concentration of 1% (v/v) and the cultures were incubated for 30 min at 30°C with shaking to fix the cells. The crosslinking reaction was quenched by adding glycine (Fisher Scientific; cat# 10467963) to the final concentration of 0.1 M, followed by a 10-min incubation at room temperature on a wheel rotator. Afterward, cells were collected at 8800 g for seven minutes at 4°C, washed twice with 25 mL of phosphate-buffered saline (pH 7.4), and resuspended in 1.5 mL of the same buffer before final centrifugation at 17000 g for 5 min at 4°C. The supernatant was then removed, and the pelleted cells were resuspended in 0.75 mL of the lysis buffer (20 mM of potassium 4-(2-hydroxyethyl)-1-piperazineethanesulfonic acid salt pH 7.9, 50 mM potassium chloride, 10% glycerol, 15 mg/mL lysozyme, and an EDTA-free protease inhibitor tablet).

The cell suspension was incubated in a 37°C water bath for 25 min, and then placed on ice before addition of 0.75 mL of the lysis buffer without lysozyme. Cells were lysed by sonication at 8 µm amplitude in 15 s pulses followed by 15 s pauses for eleven cycles in total using a Soniprep 150 ultrasonic sonicator (Sanyo). Cell debris was then pelleted by centrifugation at 17000 g for 20 min at 4°C, and the supernatant was transferred to a Lo-bind Eppendorf tube (Eppendorf; cat# 022431021). To prepare the supernatant for further processing, 1 M Tris-HCl (pH 8), 5 M sodium chloride, and 10% (v/v) Nonidet P-40 octyl phenoxypolyethoxylethanol (NP-40) were added to the supernatant to the final concentrations of 10 mM, 100 mM, and 0.1 % (v/v), respectively.

To immune-precipitate protein-DNA crosslinking products, 100 µL of anti-FLAG beads were pre-equilibrated in the IPP-150 buffer (10 mM Tris-HCl pH 8, 150 mM sodium chloride, 0.1% NP-40), added to the supernatant, and incubated overnight on a wheel rotator at 4°C. Subsequently, the beads were pelleted by centrifugation at 13000 g for 30 s at 4°C, and the beads were washed five times with 1 mL of IPP-150 each. Lastly, the beads were washed with 1 mL of TE buffer (10 mM Tris-HCl pH 7.4 and 1 mM EDTA).

Beads were resuspended in 150 µL of elution buffer (50 mM Tris-HCl pH 8, 10 mM EDTA, and 1% (w/v) sodium dodecyl sulfate), and incubated at 65°C for 15 min, to reverse the crosslinking. Afterward, beads were pelleted by centrifugation at 17000 g for 5 min at room temperature, and the supernatant was transferred to a 2 mL Lo-bind Eppendorf tube. The remaining beads were resuspended in 100 µL of TE buffer + 1% (w/w) of sodium dodecyl sulfate, and incubated for 5 min at 65°C. Beads were then pelleted as described above, and the supernatant was pooled together with the supernatant from the previous step, and incubated overnight at 65°C to completely reverse protein-DNA crosslinking.

Once the incubation was completed, the sample was centrifuged at 13000 g for three min at room temperature. Afterward, the supernatant was recovered and mixed with five volumes of PB buffer from QIAquick PCR purification kit (Qiagen; cat# 28104) and passed through a QIAquick PCR purification column to allow DNA binding to the membrane. The column was washed twice with

520 µL of PE buffer and finally centrifuged at 17000 g for two min to remove residual buffer. To elute DNA, 20 µL of distilled water was placed on the membrane, and the column was centrifuged at 13000 g for two min to collect the eluate in a 1.5 mL Lo-bind Eppendorf tube. The elution was repeated once more, and the eluate was pooled together and used to construct DNA libraries for Illumina deep sequencing using an NEB DNA Library Prep Kit (NEB; cat # E7645S). The prepared DNA libraries were stored at -80°C before being sequenced at the Tufts University Core Facilities (US).

For analysis of ChIP-seq data in **Fig. 3A**, a reference genome of *S. coelicolor* A3(2) SCP1$^+$ SCP2$^+$ ϕBT1::*parT-flag* was first constructed by concatenating the sequences of a linear chromosome (Genbank ID: AL645882.2) (with the sequence of pIJ10257::*parT-flag* plasmid inserted at the ϕBT1 locus), a linear plasmid SCP1 (Genbank ID: AL589148.1), and a circular plasmid SCP2 (Genbank ID: AL645771.1) together. For analysis of ChIP-seq data in **Fig. 6B**, a reference genome of *S. coelicolor* M600 SCP1$^-$ SCP2$^-$ ϕBT1::*parT-flag* was first constructed by concatenating the sequences of a linear chromosome (Genbank ID: AL645882.2) (with the sequence of pIJ10257::*parT (WT/variant)-flag* plasmid inserted at the ϕBT1 locus). Subsequently, Hiseq 2500 (50 bp) or NextSeq 550 Illumina (75 bp) short reads were mapped back to this reference genome using Bowtie 1(4) and the following command: bowtie -M 1 -n 1 –best –strata -p 4 –chunkmbs 512 A32-bowtie –sam *.fastq > output.sam. Subsequently, the sequencing coverage at each nucleotide position was computed using BEDTools (5) using the following command: bedtools genomecov -d -ibam output.sorted.bam -g A32.fna > coverage_output.txt. ChIP-seq profiles were plotted with the x-axis representing genomic positions and the y-axis is the number of reads per base pair per million mapped reads (RPBPM) or number of reads per kb per million mapped reads (RPKPM) using custom R scripts. For the list of ChIP-seq datasets in this study, see **Table S6**.

Identification of ParT structural homologues by FoldSeek

The amino acid sequence of ParT (UniProt database entry: Q8VWE5) was used to build an AlphaFold2 model of a ParT dimer, which was then used to query FoldSeek for structurally similar proteins in the AlphaFold Database (6, 7). Afterward, upon removal of duplicate entries, we used a bespoke Perl script utilizing the Net::FTP module to download associated genomic assemblies where these were available in the NCBI bacterial genome assemblies collection. To search for structural homologs of ParT that possibly constitute a ParAB*S* system, we created a consensus ParA sequence based on the collection of experimentally confirmed ParA enzymes (**Supporting Dataset 3**) and used BLAST2.13.0 search (8) with an expectation value E = $1x10^{-9}$; followed by enforcing the percentage of identity to 30% or more and percentage of coverage of queries and subjects to 50% or more to identify ParA-like enzymes localized on the same contigs with ParT structural homologs.

To establish evolutionary relationship between ParT homologues, the corresponding protein sequences were aligned using MUSCLE algorithm and the output used to construct a maximum likelihood tree with RAxML version 8.2.12(9) using the Geneious CIPRES REST API Plug-In (10) . The rate heterogeneity and substitution models selected were Protein GAMMA and BLOSSUM62 with no correction for ascertainment bias. The full job input parameters are as follows:

raxmlHPC-HYBRID-AVX -T 4 -f a -n result -s infile.txt -p 12345 -m PROTGAMMABLOSUM62X -k -x 12345 --asc-corr lewis. The resulting tree, containing 113 entries, was visualized and formatted using iTOL(11).

Mass photometry measurements of ParT oligomeric state

All mass photometric measurements were recorded at 25°C using the Refeyn OneMP mass photometer (Refeyn Ltd, UK). The instrument was calibrated with a set of calibrants, β-amylase (56, 112, and 224 kDa), BSA (66 kDa), and urease (90.7, 272, and 545 kDa). A stock solution of 1 µM ParT (WT)-His_6 in a buffer containing 10 mM Tris-HCl pH 8.0 and 250 mM sodium chloride was diluted in PBS buffer on a coverslip to the final concentrations of 100 nM, 50 nM, and 25 nM. Movies were recorded by using AcquireMP software (version R1) for 60 s with a frame rate of 60 per s and using a large field of view. The data were processed using DiscoverMP software (version R1.2). The

mass of ParT-His$_6$ was estimated by fitting a Gaussian distribution into mass histograms and taking the value at the mode of the distribution.

Design and construction of a large plasmid with 3x *parS* sites for confocal optical tweezers (C-Trap) experiments

The large DNA plasmid containing 3 copies of the 22 bp-inverted repeat sequence *parS* (CGTGTCTCCAATTGGAGACATC) was produced as follows. First, a large DNA plasmid with a single *parS* site was fabricated by ligating a dsDNA duplex containing a single copy of the *parS* site into a large plasmid previously prepared in our laboratory that did not contain this site. This previous large plasmid named as p64.large plasmid backbone (20482 bp, **Table S3**) had been fabricated by ligation of three DNA pieces following the protocol described previously (12). The dsDNA duplex with the *parS* site was obtained by annealing two oligonucleotides (**Table S5**) by heating at 95˚C for 5 min and cooling down to 20˚C at a rate of -1˚C min^{-1} in hybridization buffer (10 mM Tris-HCl pH 8.0, 1 mM EDTA, 200 mM NaCl, and 5 mM $MgCl_2$) followed by a phosphorylation step of the 5´-terminal ends by the T4 PNK (NEB). This dsDNA duplex was ligated into p64.large plasmid backbone digested with KpnI (NEB) and dephosphorylated with rSAP (NEB). This resulted in a large plasmid with a single *parS* site (p145A.1x *parS,* 20532 bp, **Table S3**). To fabricate the plasmid with 3x *parS* sites, the previous large plasmid containing 1x *parS* site was digested with NruI (NEB) and dephosphorylated. A new dsDNA duplex was prepared by annealing two oligonucleotides containing 2x *parS* sites separated by 40 bp. These oligonucleotides were designed to contain several internal restriction sites flanking the *parS* sites. These restriction sites were used to digest the duplex with different pairs of restriction enzymes generating different ends. The diverse dsDNA duplexes were used in different ligation steps during the fabrication of the plasmids for magnetic tweezers experiments (see below). For this specific cloning, the dsDNA duplex once annealed was digested with SfoI and HpaI (NEB). The digested dsDNA duplex was ligated into the linearized p145A.1x *parS* plasmid, resulting in a large plasmid with 3x *parS* sites (p145B.3x *parS*, 20622 bp, **Table S3**). Plasmids were introduced into *E. coli* DH5α competent cells and potentially positive colonies were then verified by colony PCR(12) . Plasmids were purified from the cultures using a QIAprep Spin Miniprep Kit (QIAGEN), analyzed by restriction enzyme digestion, and finally verified by Sanger sequencing. This plasmid was used to produce a C-Trap dsDNA construct (see below).

Design and construction of a DNA plasmid with 5x *parS* sites for magnetic tweezers (MT) experiments

A DNA plasmid containing 5x *parS* sites was produced by following several cloning steps. To fabricate these plasmids, we employed an enlarged pUC19 plasmid of 7699 bp (pUC19_v2, **Table S3**) (13). First, this plasmid was digested with KpnI and PshAI (NEB), and the vector fragment of 6190 bp was gel extracted (QIAGEN). On the other hand, a small fragment containing a single *parS* site was produced by PCR amplifying a region of the large plasmid p145A.1x *parS* described above with Phusion High-Fidelity DNA Polymerase (Thermo Scientific) (**Table S5**). The PCR fragment was then digested with KpnI in one end, followed by phosphorylation of the blunt end with T4 PNK and ligated into the 6190 bp vector fragment. This resulted in a plasmid of 6417 bp with 1x *parS* site (p144A.1x *parS,* **Table S3**).

To increase the number of *parS* sites the annealed two long oligonucleotides containing 2x *parS* sites described above for the C-trap large plasmid p145B.3x *parS* (**Table S5**) were employed. To fabricate a plasmid with 3x *parS* sites, the dsDNA duplex with 2x *parS* sites digested with SfoI and HpaI was ligated into the plasmid with 1x *parS* site digested with SfoI and dephosphorylated. This resulted in a plasmid with 3x *parS* sites (p144B.3x *parS*, 6507 bp, **Table S3**). To fabricate a plasmid with 5x *parS* sites, the dsDNA duplex with 2x *parS* sites digested with XhoI and SalI (NEB) was ligated into the plasmid with 3x *parS* sites digested with XhoI and dephosphorylated. This resulted in the final plasmid with 5x *parS* sites (p144C.5x *parS* 6614 bp, **Table S3**). The plasmids were cloned and analyzed as described for C-Trap plasmids. This plasmid with 5x *parS* sites was used to prepare a magnetic tweezers dsDNA construct (see below).

Magnetic tweezers dsDNA construct with 5x *parS* sites

The dsDNA construct for magnetic tweezers experiments consisted of a central dsDNA fragment of 6602 bp containing 5x *parS* sites, obtained by digestion with NotI and ApaI (NEB) of the final MT plasmid p144C.5x *parS* described above, flanked by two highly labeled DNA fragments, one with digoxigenins and the other with biotins, of 997 bp and 140 bp, respectively, used as immobilization handles. The biotinylated handle was shorter to minimize the attachment of two beads per DNA tether. Handles for MT constructs were prepared by PCR (**Table S5**) with 200 µM final concentration of each dNTP (dGTP, dCTP, dATP), 140 µM dTTP, and 66 µM Bio-16-dUTP or Dig-11 dUTP (Roche) using plasmid pSP73-JY0 (14) as template, followed by digestion with the restriction enzyme ApaI or PspOMI (NEB), respectively. The Dig-PspOMI handle was dephosphorylated with rSAP to fabricate non-torsionally constrained tethers. Labeled handles were ligated to the central part overnight using T4 DNA Ligase (NEB). The sample was then ready for use in MT experiments without further purification. The DNAs were never exposed to intercalating dyes or UV radiation during their production and were stored at 4°C.

C-Trap dsDNA construct with 3x *parS* sites
C-Trap experiments were performed on dsDNA molecules of 20622 bp containing 3x *parS* sites. The central part of the dsDNA construct was obtained by digestion of the large C-Trap plasmid p145B.3x *parS* described above with NotI (NEB) produced following published protocols (12). Without further purification, the fragment was ligated to highly biotinylated handles of ~1 kb ending in PspOMI. Handles for C-Trap constructs were prepared by PCR (**Table S5**) as described for biotin-labeled MT handles. These handles were highly biotinylated to facilitate the capture of DNA molecules in the C-Trap experiments. As both sides of the DNA fragment end in NotI, it is possible to generate tandem (double length) tethers flanked by the labeled handles. The sample was ready for use in C-Trap experiments without further purification. The DNAs were not exposed to intercalating dyes or UV radiation during their production and were stored at 4˚C.

A control dsDNA construct of 20482 bp without *parS* sites was similarly prepared but using as central part the fragment corresponding to the linearization of the large plasmid p64.large plasmid backbone that did not contain any *parS* site.

Bacterial-two hybrid assay
Bacterial-two hybrid assays were performed exactly as described in the Euromedex manual. Briefly, *E. coli* BTH101 cells were co-transformed with a pair of plasmids by electroporation before being plated out on LB + carbenicilin + kanamycin and incubated at 30°C. Three colonies from each plasmid combination were grown up in LB + carbenicilin + kanamycin overnight. To measure β-galactosidase activities, we used overnight cultures. Three biological replicates were performed to obtain the mean and standard deviation of β-galactosidase activities.

Nucleotide Content analysis
Purified ParT (WT) (60 µL, 6.0 mg/mL) was mixed with MeOH (60 µL) and the sample was agitated vigorously for 60 sec and then subjected to centrifugation (9,391 x g, 10 min, ambient temperature). The supernatant was removed and analysed by liquid chromatography coupled with electrospray ionization tandem mass spectrometry (LC-ESI-MS/MS)(15). Separation was achieved on an Acquity UPLC using surface-conditioned porous graphitic carbon (PGC) column (Hypercarb) and the target analytes were detected by negative electrospray ionization using Xevo TQ Absolute triple quadrupole mass spectrometer (Waters) operated in MRM mode. ESI-MS/MS analysis was performed in negative ion mode using a source with a capillary voltage of 1.5 kV, 600 °C desolvation temperature, 900 l/h desolvation gas, 150 l/h cone gas, and 7 bar nebulizer pressure. MRM transitions for nucleotide standards in negative ESI mode (see table below) were generated using IntelliStart software. Samples of nucleotide standards (10 µM, ADP, GDP, CDP, UDP, ATP, GTP, CTP and UTP) were introduced at 10 µL/min combined with a flow from the UPLC pump typical of an LC run. Once LC retention times of standards have been established, the mass transitions were collected in time-windows centered on the relevant peaks, to avoid collecting excessive numbers of transitions simultaneously. MassLynx software (Waters) was used to collect, to analyze and to process data. Liquid chromatography separation of nucleotides was achieved on a surface-

conditioned PGC column (Hypercarb, Thermo Scientific, dimensions 1 x 100 mm, particle size 5 µm) equipped with a column guard (Hypercarb, 5 µm, 1 x 10 mm). Nucleotides were eluted using mobile phase A: formic acid 0.3% brought to pH 9.0 with ammonia and mobile phase B: acetonitrile using the following multistep gradient at a flow rate 80 µL/min: 0 min: 2% B; 20 min: 10% B; 26 min: 50% B; 27 min: 90% B; 30 min: 90% B; 31 min: 2% B; 50 min: 2% B. Nucleotide standards (10 µM) were injected (5 µL) to determine retention times. Limit of detection for CTP was determined to be 10 fmol on column using a serial dilution. To ensure analyte retention time (R_f) stability the PGC column had to be regenerated before use(16). The regeneration was performed using a standard HPLC system (Ultimate 3000, Dionex) with UV detection at 265 nm. The column was first washed in mobile phase A for 10 min, followed by a 3 h wash in mobile phase C (acetonitrile 80%, water 20 %, TFA 0.1%). The column was then flushed with 10% A and 90% B for 30 min, followed by a last wash of 100% A for 10 min. All wash steps were done at a 0.1 ml/min. The column was then reduced with freshly prepared sodium sulfite (100 mM) for 22 h, using a flow rate gradient from 0.08 ml/min to 0.1 ml/min over that period. The regeneration of the column was followed by a wash in 100% A at 0.1 ml/min for 1 h, and a wash in 10% A and 90% B for 30 min, before the column was equilibrated in 98% A and 2 % B for 10 min.

Table S2. MRM transitions of nucleotides analysed in this work

Nucleotide	MRM transitions	Cone [V]	Collision Energy [eV]	Fragment
ADP	426 → 134<br>426 → 159	34.00<br>34.00	20.00<br>24.00	$[N\text{-}H]^-$<br>$[H_4P_2O_7\text{-}H_3O]^-$
GDP	442 → 150<br>442 → 159	12.00<br>12.00	28.00<br>26.00	$[N\text{-}H]^-$<br>$[H_4P_2O_7\text{-}H_3O]^-$
CDP	402 → 110<br>402 → 159	22.00<br>22.00	22.00<br>28.00	$[N\text{-}H]^-$<br>$[H_4P_2O_7\text{-}H_3O]^-$
UDP	403 → 111<br>403 → 159	10.00<br>10.00	18.00<br>26.00	$[N\text{-}H]^-$<br>$[H_4P_2O_7\text{-}H_3O]^-$
ATP	506 → 134<br>506 → 159	22.00<br>22.00	42.00<br>40.00	$[N\text{-}H]^-$<br>$[H_4P_2O_7\text{-}H_3O]^-$
GTP	522 → 159<br>522 → 424	30.00<br>30.00	34.00<br>20.00	$[H_4P_2O_7\text{-}H_3O]^-$<br>$[NDP\text{-}H\text{-}H_2O]^-$
CTP	482 → 159<br>482 → 384	32.00<br>32.00	32.00<br>20.00	$[H_4P_2O_7\text{-}H_3O]^-$<br>$[NDP\text{-}H\text{-}H_2O]^-$
UTP	483 → 159<br>483 → 385	28.00<br>28.00	30.00<br>18.00	$[H_4P_2O_7\text{-}H_3O]^-$<br>$[NDP\text{-}H\text{-}H_2O]^-$

Magnetic tweezers experiments

Magnetic tweezers experiments were performed using a homemade setup that was previously described (17, 18). Briefly, optical images of micron-sized superparamagnetic beads tethered to a glass surface by DNA substrates were acquired using a 100x oil immersion objective and a CCD camera operating at 120 Hz. Real-time image analysis allows the spatial coordinates of the beads to be determined with nm accuracy in the x, y, and z directions. We controlled the stretching force of the DNA by using a step motor coupled to a pair of magnets located above the sample. The applied force is quantified from the Brownian motion of the bead and the extension of the tether, obtained by direct comparison of images taken at different focal planes (19, 20).

Magnetic tweezers experiments were performed as follows. First, a double PARAFILM (Sigma)-layer flow chamber was incubated at 4ºC overnight with 25ng/µL Digoxigenin Antibody (Bio-Rad) that adsorbed onto the polystyrene-covered lower surface. During the experiment 8 µL of a DNA containing 5x *parS* (1.4nM) was diluted 1:300 in TE (10mM Tris pH 8, 1 mM EDTA) and mixed with 20 µl of 1 µm-diameter magnetic beads (Dynabeads MyOne Streptavidin T1, Thermo Fisher Scientific) diluted 1:10 in PBS-BSA (0.4 mg/mL BSA (NEB)). After 10 minutes the excess of DNA in

solution was removed by precipitating the beads with a magnet and discarding all the supernatant volume. Beads were then washed three times with fresh PBS-BSA and resuspended in 80 μl before flushing into the flow cell. The DNA-beads were then incubated in the chamber for 15 minutes to promote the interaction of the digoxigenin handle with the anti-digoxigenin surface. The excess beads were washed away by flushing around 1 ml PBS. Torsionally constrained molecules and beads containing more than a single DNA molecule were identified from their distinct rotation-extension curves and discarded for further analysis. Force-extension curves were generated by measuring the extension of the tethers at decreasing forces from 5 pN to 0.006 pN. The curves were first measured on naked DNA molecules and then the experiment was repeated using 1 μM of *Bacillus subtilis* ParB or 1 μM ParT in the reaction buffer (100 mM Tris pH 8, 100 mM NaCl, 1 mM $MgCl_2$, 1 mM DTT and 0.1 mg/mL BSA) supplemented with 2 mM CTP. Data were analyzed and plotted using Origin software.

Confocal optical tweezers experiments

Confocal-optical tweezers experiments were carried out using a dual optical tweezers setup combined with confocal microscopy and microfluidics (C-Trap; Lumicks). A computer-controlled stage allowed rapid displacement of the optical traps within a five-channel fluid cell, allowing the transfer of the tethered DNA between different channels separated by laminar flow. Channel 1 contained 4.34 µm streptavidin-coated polystyrene beads (Spherotech). Channel 2 contained the DNA substrate harboring 3x *parS* sequences or the control DNA without *parS* both of them labeled with multiple biotins at both ends. DNA and beads were diluted in 20 mM HEPES pH 7.8, 100 mM KCl, and 5 mM $MgCl_2$. A single DNA tether was assembled by first capturing two beads in channel 1, one in each optical trap, and fishing for a DNA molecule in channel 2. The tether was then transferred to channel 3 filled with reaction buffer (100 mM Tris pH 8, 100 mM NaCl, 1 mM $MgCl_2$, and 1 mM DTT) to verify the length of the correct length of the DNA by force-extension curves. The DNA was then incubated in channel 4 filled with 1 μM ParT. To reduce the fluorescence background in single ParT diffusion measurements, imaging was performed in channel 3 after protein incubation in channel 4.

To visualize the AF488-HALO-ParT, a 488 nm laser was used for excitation, and emission was detected using a 500-525 nm filter. Protein-containing channels were passivated with BSA (0.1% w/v in PBS) for 30 min before the experiment. Kymographs were generated by single line scans between the two beads using a pixel size of 100 nm and a pixel time of 0.1 ms, resulting in a typical time per line of ~22 ms. The confocal laser intensity at the sample was 2.2 µW. Experiments were performed in constant-force mode at 15 pN.

For the calculation of the diffusion coefficient, we used custom Python scripts to access, visualize, and export confocal data from Bluelake (Lumicks) HDF5 files obtained from C-Trap experiments. The quantification of the individual ParT trajectories was done by using a custom LabVIEW software that provides the position of individual proteins along the DNA for a given time (t). The length of the time courses was restricted to 2.5 sec to increase the statistical sample. The mean square displacement (MSD) was then calculated for a given time interval (Δt) and the diffusion coefficient (*D*) was obtained as described in (17, 21, 22). A total of 154 trajectories were used for the diffusion coefficient calculation.

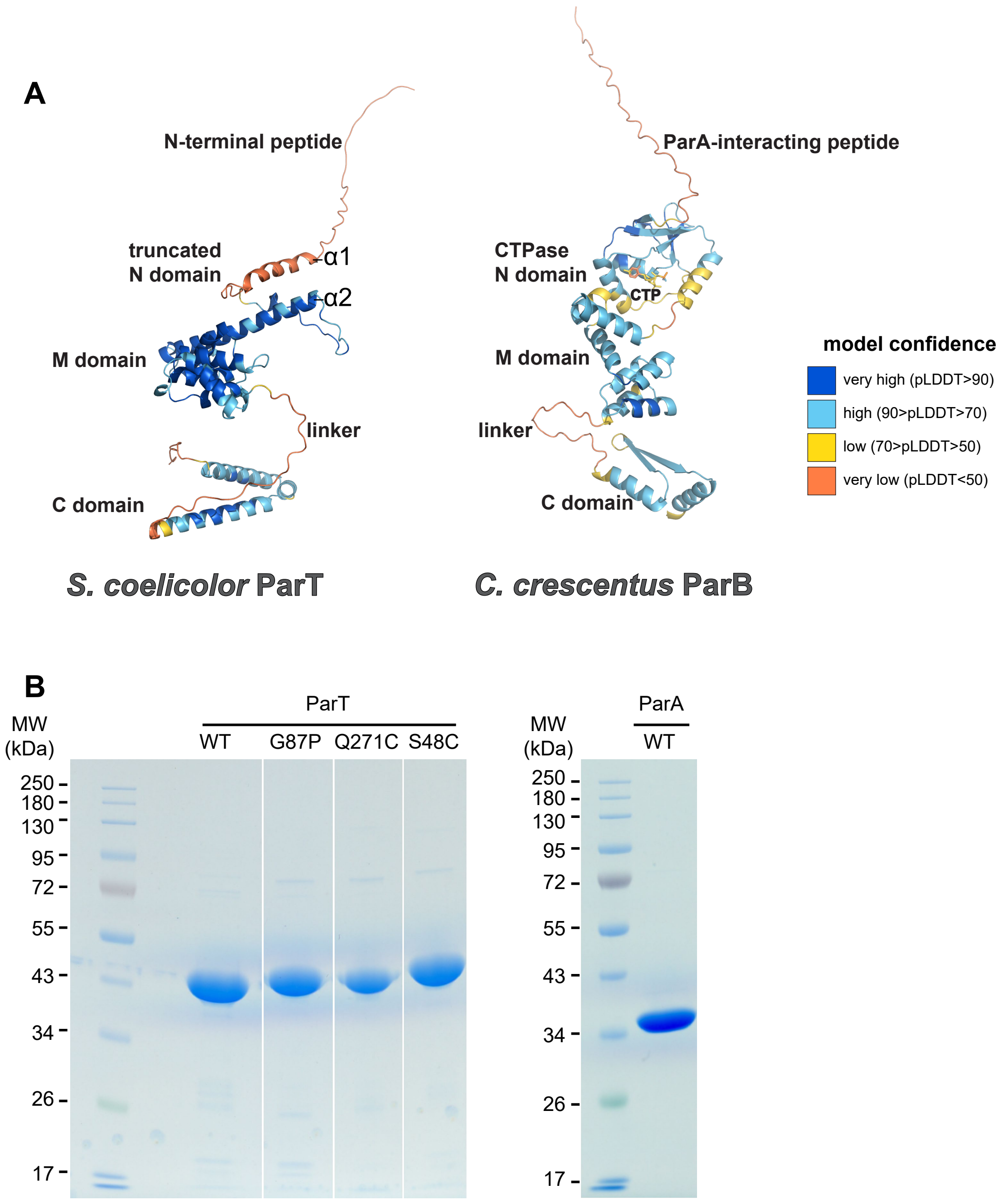

Fig. S1. *S. coelicolor* ParT lacks a CTPase domain. (A) AlphaFold2-predicted structures of *S. coelicolor* ParT and *C. crescentus* chromosomal ParB. Structures were colored according to the model confidence score (pLDDT). **(B)** SDS-PAGE analysis of purified ParT (WT/variants) and purified ParA used in this study.

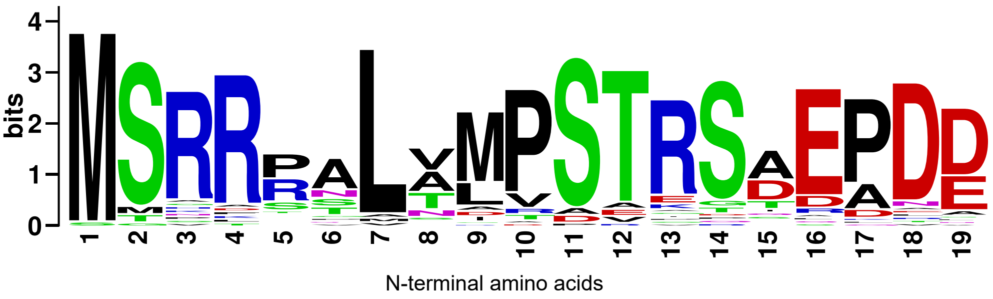

Fig. S2. Amino acid conservation at the N-terminus of ParT homologs. Sequences of the first 19 amino acids of proteins encoded by *parT* homologs with adjacent *parA* genes were aligned and subsequently showed by a WebLogo representation. Amino acids were colored based on their chemical properties (GSTYC, polar; QN, neutral; KRH, basic; DE, acidic; and AVLIPWFM, hydrophobic).

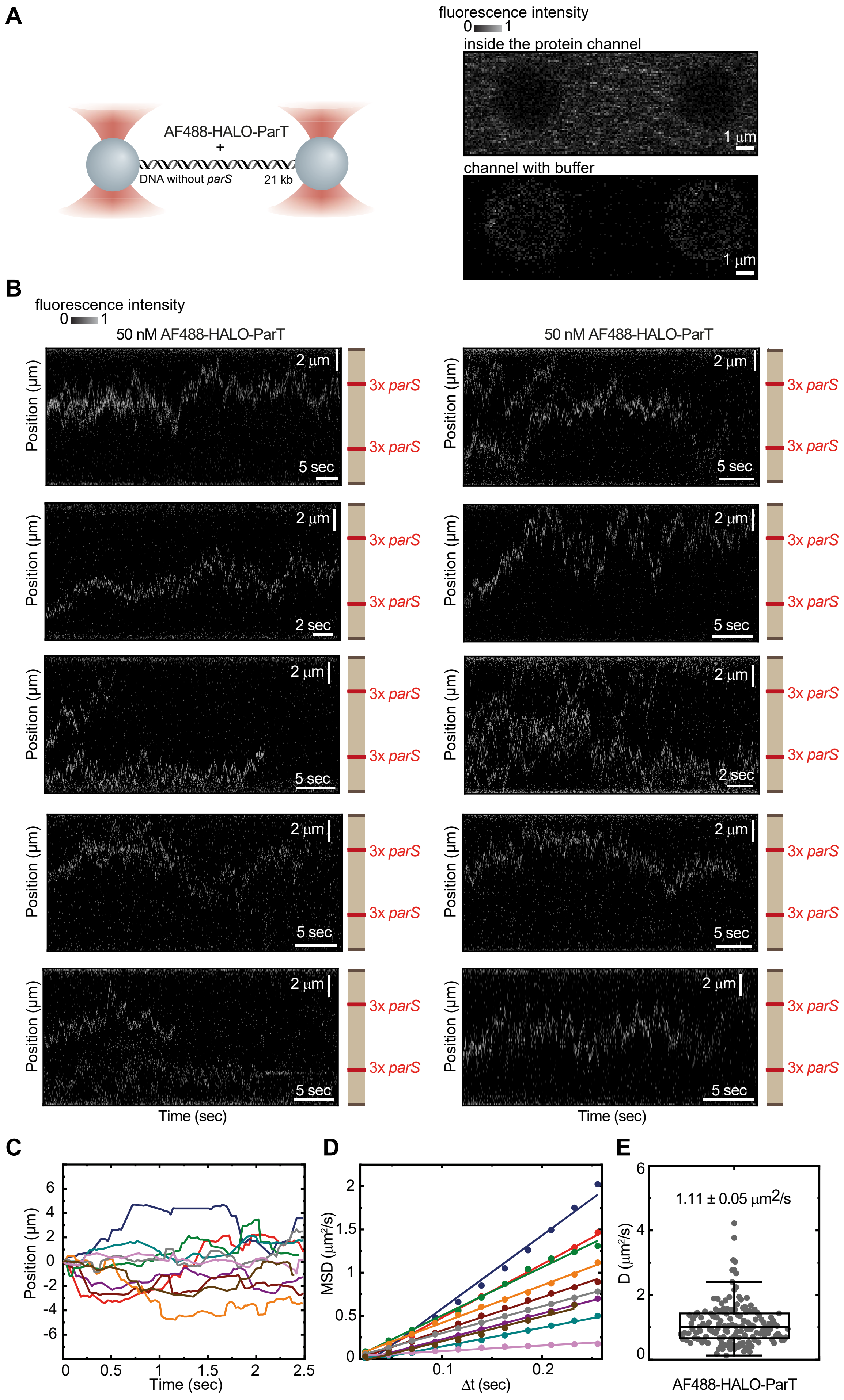
A
fluorescence intensity
0 1
inside the protein channel
1 μm
channel with buffer
1 μm
AF488-HALO-ParT
+
DNA without parS
21 kb
B
fluorescence intensity
0 1
50 nM AF488-HALO-ParT
50 nM AF488-HALO-ParT
Position (μm)
2 μm
5 sec
2 sec
3x parS
Time (sec)
C
Position (μm)
Time (sec)
D
MSD (μm²/s)
Δt (sec)
E
D (μm²/s)
1.11 ± 0.05 μm2/s
AF488-HALO-ParT

Fig. S3. ParT diffuses on *parS* DNA independently of NTP. (A) AF488-HALO-ParT did not bind DNA lacking *parS* site. (left panel) Representative cartoon showing a 21-kb DNA, without a *parS* site, trapped between the two beads in the optical tweezers. (upper right panel) A scan showing DNA trapped between two beads, inside a protein channel containing 50 nM AF488-HALO-ParT. (lower right panel) the same DNA molecule, after a minute of incubation with AF488-HALO-ParT in the protein channel, was subsequently transferred to a channel with buffer. We did not observe any binding event, only a high background from the fluorescently labeled protein. **(B)** Additional representative kymographs showing AF488-HALO-ParT binding and diffusing on *parS*-containing DNA. **(C)** Representative AF488-HALO-ParT trajectories were measured on the DNA (n=154). **(D)** Mean squared displacement (MSD) of AF488-HALO-ParT trajectories for different time intervals (Δt). The diffusion constant of ParT was calculated as half of the slope of the linear fit of MSD versus Δt. **(E)** Boxplot showing the diffusion constant of individual ParT diffusion on *parS* DNA ($D = 1.11 \pm 0.05$ $\mu m^2/s$, mean ± standard error of measurement (SEM), n = 154).

A

positive control (a mixture of ATP, GTP, CTP, and UTP at 25 μM)

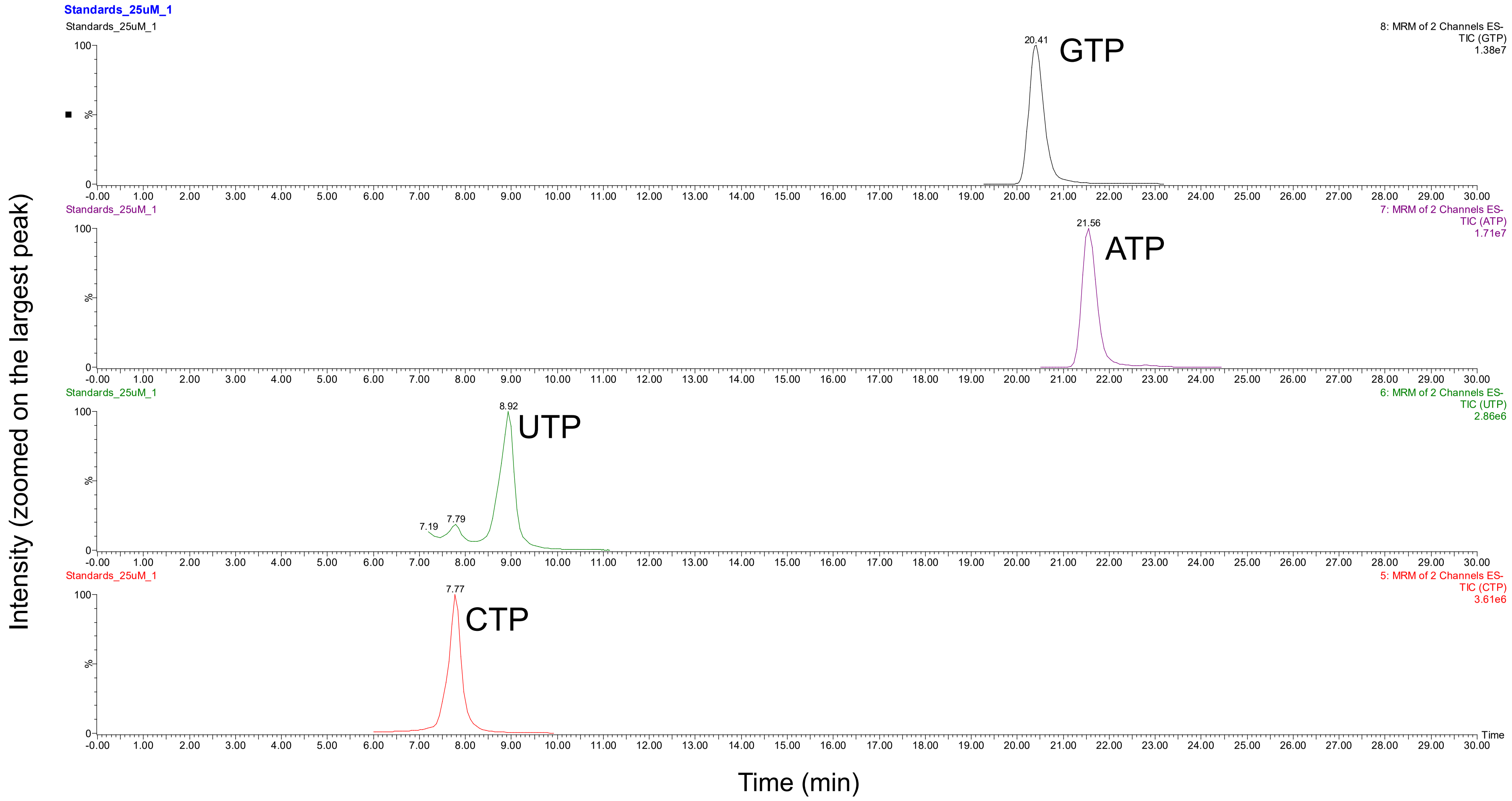

B

Intensity (zoomed on the largest peak)

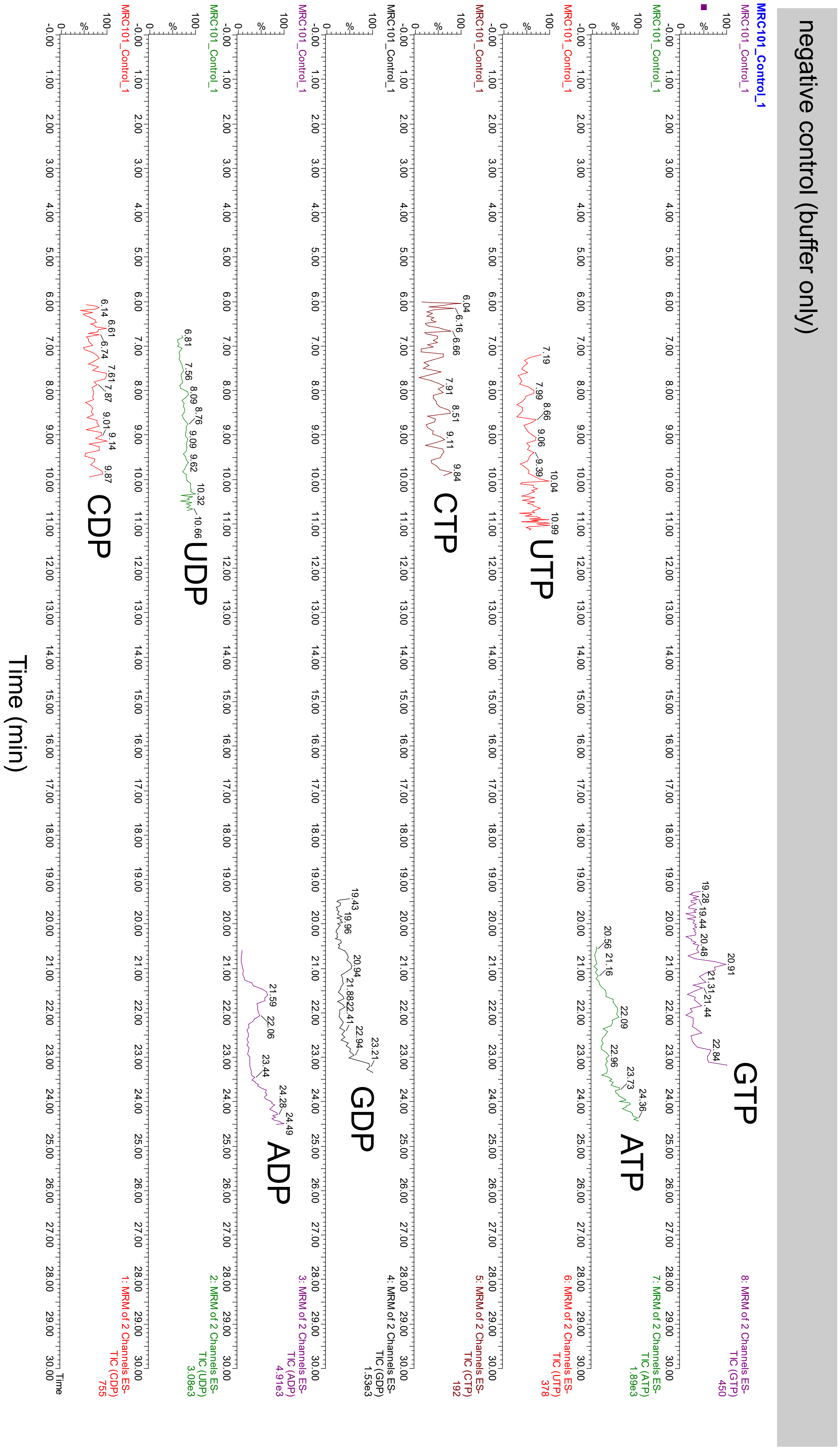

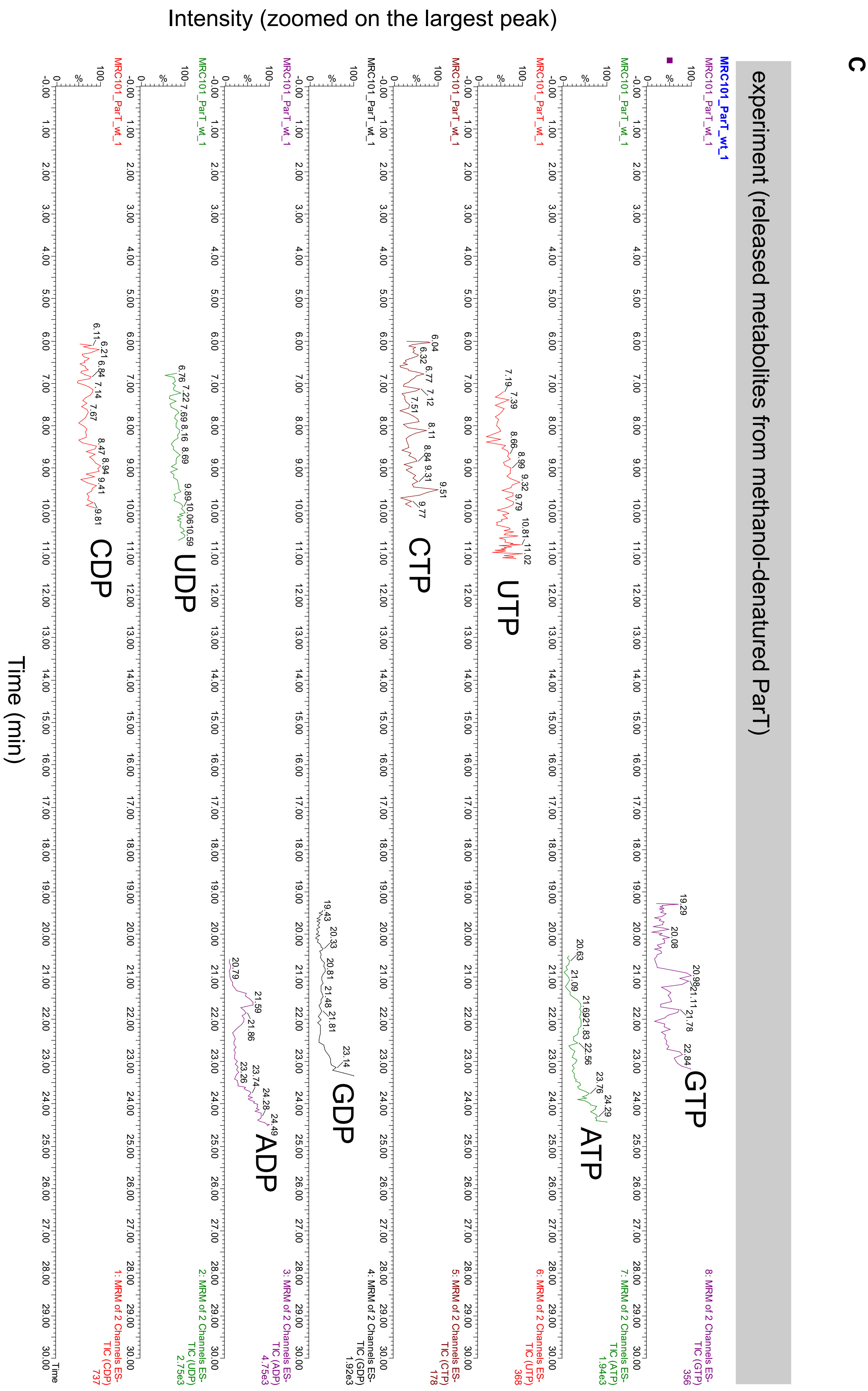
c
experiment (released metabolites from methanol-denatured ParT)
Intensity (zoomed on the largest peak)
Time (min)
GTP
ATP
UTP
CTP
GDP
ADP
UDP
CDP
MRC101_ParT_wt_1
8: MRM of 2 Channels ES-
TIC (GTP)
356
7: MRM of 2 Channels ES-
TIC (ATP)
1.94e3
6: MRM of 2 Channels ES-
TIC (UTP)
368
5: MRM of 2 Channels ES-
TIC (CTP)
178
4: MRM of 2 Channels ES-
TIC (GDP)
1.92e3
3: MRM of 2 Channels ES-
TIC (ADP)
4.75e3
2: MRM of 2 Channels ES-
TIC (UDP)
2.75e3
1: MRM of 2 Channels ES-
TIC (CDP)
737

Fig. S4. ParT did not co-purify with nucleotide triphosphates. Nucleotide content analysis by liquid chromatography with tandem mass spectrometry (LC-MS/MS) of purified ParT after denaturation by methanol. **(A)** a positive control (a mixture of NTPs at 25 μM each) showed well-defined peaks and detection of each NTP by LC-MS/MS. **(B)** a negative control (buffer only) showed background level of NTPs or NDPs. **(C)** When the released metabolites from methanol-denatured ParT was subjected to LC-MS/MS, no reliable peak were detected and only background level of NTPs/NDPs were observed, indicating that purified ParT did not co-purify with NTPs or NDPs.

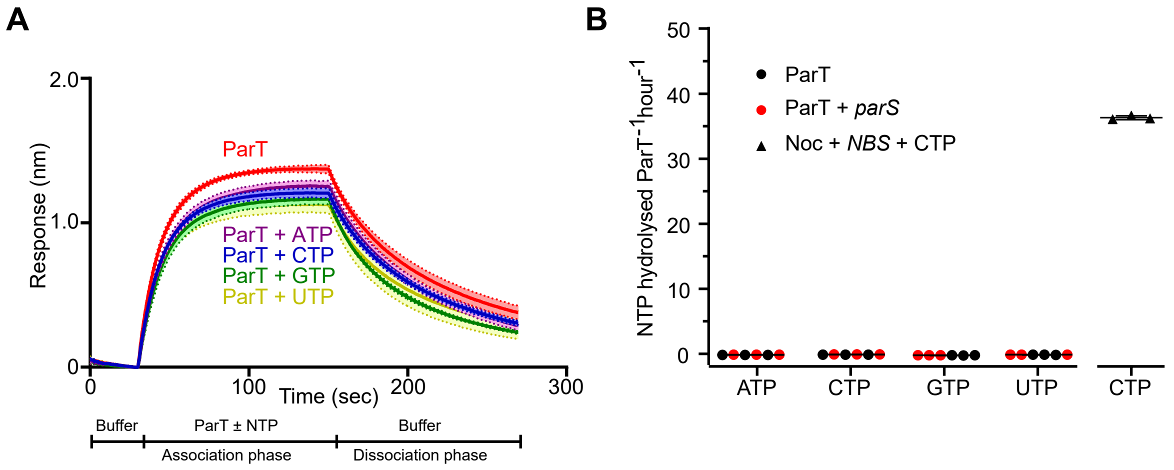

Fig. S5. Accumulation of ParT on a 180-bp *parS* closed DNA loop did not change significantly in the presence of NTP. (A) BLI analysis of the interaction between a premix of 1 µM ParT with/without 1 mM NTP with a 180-bp dual biotin-labeled DNA that contains either a *parS* or a scrambled *parS* site. Mean and standard deviation (shading) from three replicates are shown. **(B)** ParT does not have detectable NTPase activity compared to a ParB-like CTPase positive control, Noc (23). NTPase activities were measured at 1 µM of ParT/Noc and 1 mM NTP, in the presence or absence of 1 µM *parS* DNA or Noc-binding site (*NBS*) DNA. Experiments were performed in triplicates.

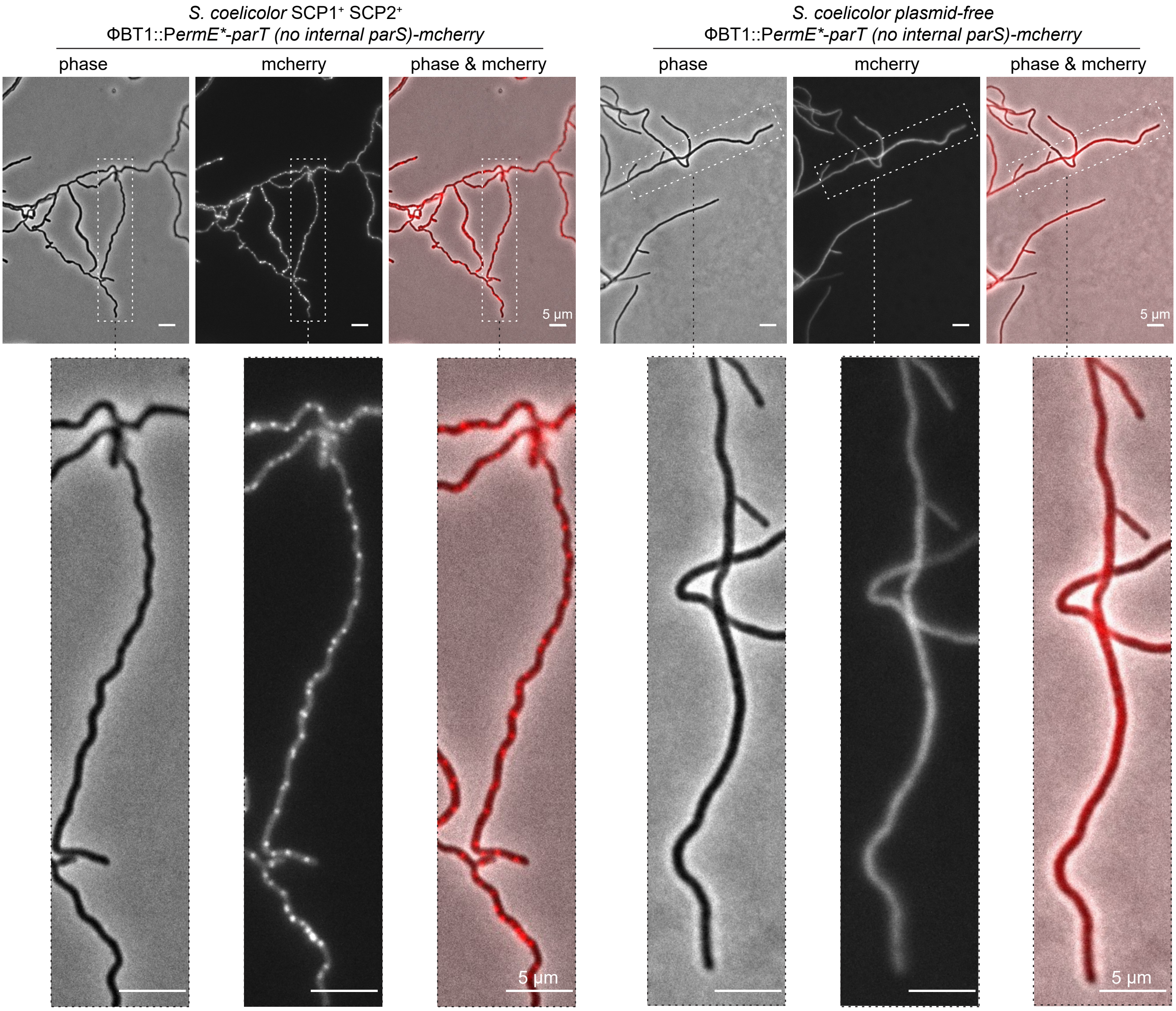

Fig. S6. Mcherry-labeled ParT formed bright foci *in vivo* in an SCP2-dependent manner. We visualized an mCherry-tagged ParT expressed either in *S. coelicolor* A3(2) carrying the SCP2 plasmid (left panel) or a plasmid-free *S. coelicolor* M600 strain (right panel). To ensure that the only *parS* site present was on the SCP2 plasmid, the internal *parS* site within the coding sequence of *mCherry*-tagged *parT* was mutated while maintaining the coded amino acid sequence.

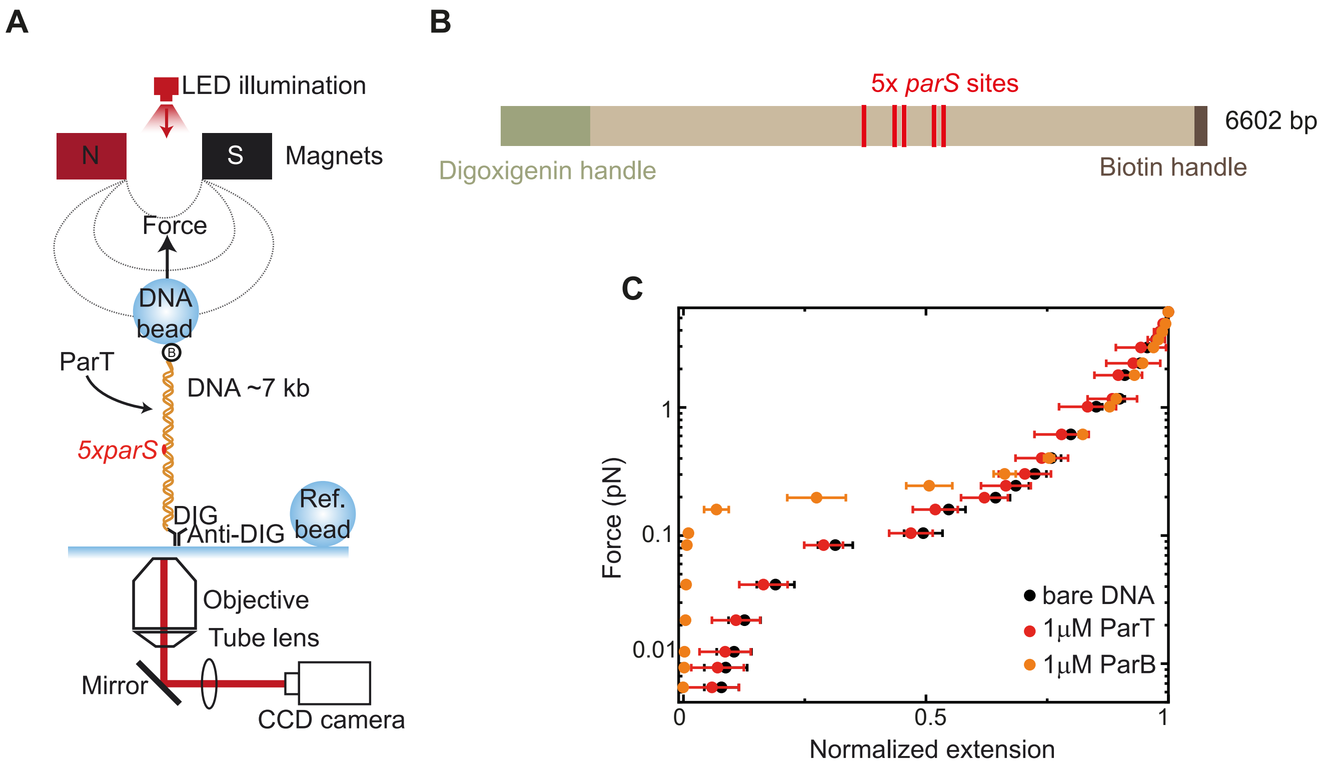

Fig. S7. ParT did not condense a DNA containing 5x *parS* sites. (A) Schematic diagram of the basic magnetic tweezers (MT) components and the layout of the experiment. **(B)** A schematic representation of a 5x *parS* DNA, the position of the *parS* sites is represented to scale. **(C)** Average force-extension curves (mean ± SEM) of bare 5x *parS* DNA molecules (n = 29) and in the presence of 1 μM ParT + 2 mM CTP (n = 29) or 1 μM *Bacillus subtilis* ParB + 2mM CTP (n= 11). Note that *B. subtilis* ParB here non-specifically condenses DNA at high concentrations (1–2 μM), as previously reported (17, 24). *B. subtilis* ParB has a different loading sequence *parS* to the *parS* site from SCP2 plasmid.

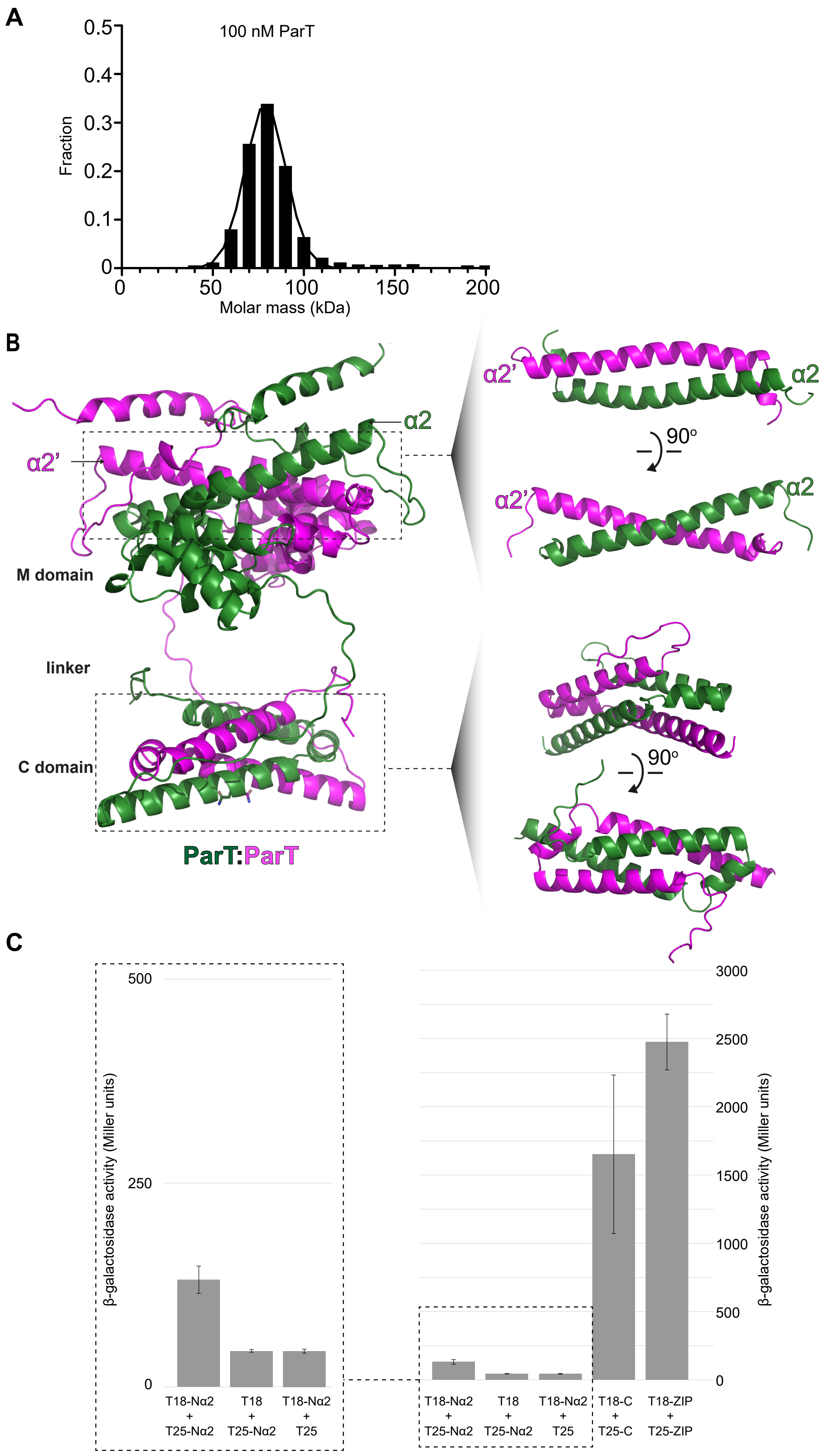
A
100 nM ParT
Fraction
0.5
0.4
0.3
0.2
0.1
0
0
50
100
150
200
Molar mass (kDa)
B
α2
α2'
M domain
linker
C domain
ParT:ParT
α2'
α2
90°
α2'
α2
90°
C
500
250
0
β-galactosidase activity (Miller units)
T18-Nα2 + T25-Nα2
T18 + T25-Nα2
T18-Nα2 + T25
3000
2500
2000
1500
1000
500
0
T18-Nα2 + T25-Nα2
T18 + T25-Nα2
T18-Nα2 + T25
T18-C + T25-C
T18-ZIP + T25-ZIP
β-galactosidase activity (Miller units)

Fig. S8. ParT can dimerize at both the N and C domains. (A) Mass photometry indicated that ParT is a dimer in solution. Mass photometry measurements were performed at room temperature at 100 nM ParT. The mass distribution curve was merged from three independent mass-photometry experiments. **(B)** An AlphaFold2 Multimer-predicted structure of a ParT dimer and its dimerization interfaces at the N domain and C domain. **(C)** ParT N and C domain self-interact. Helix α2 at the N domain or the C domain of ParT were expressed from an IPTG-inducible promoter as a C-terminal fusion to the T18 fragment or the T25 fragment of *Bordetella pertussis* adenylate cyclase. Interactions between partners were assessed by β-galactosidase assay. Three biological replicates were performed for each pair of interacting partners. Negative control (T18 or T25 fragment alone) and a positive control (T25-ZIP and T18-ZIP) were also included. Helix α2 at the N domain of ParT (Nα2) self-interacted weakly, but reliably more than the negative controls.

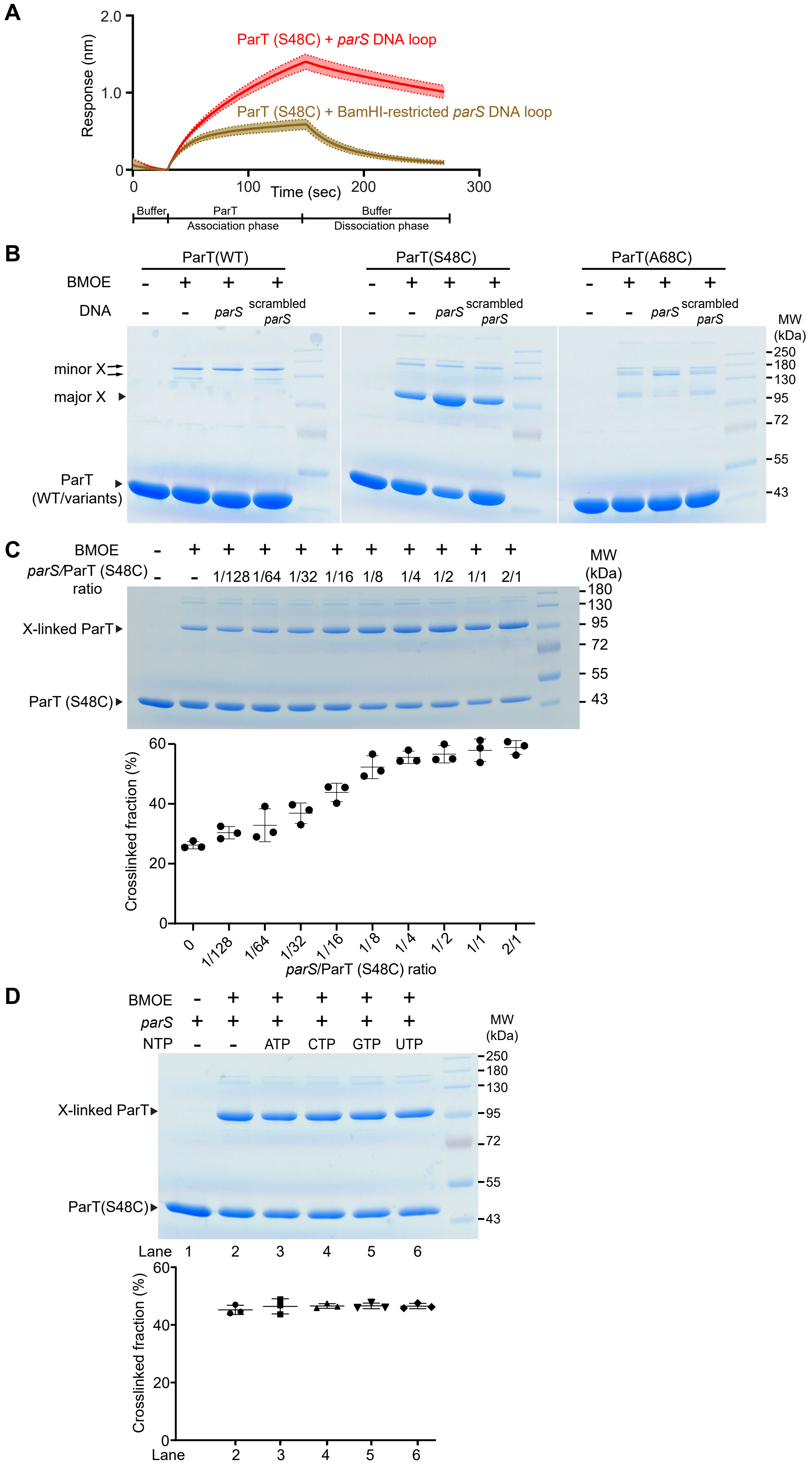
A
Response (nm)
2.0
1.0
0
ParT (S48C) + parS DNA loop
ParT (S48C) + BamHI-restricted parS DNA loop
0
100
200
300
Time (sec)
Buffer
ParT
Association phase
Buffer
Dissociation phase
B
ParT(WT)
ParT(S48C)
ParT(A68C)
BMOE
DNA
parS
scrambled parS
minor X
major X
ParT (WT/variants)
MW (kDa)
250
180
130
95
72
55
43
C
BMOE
parS/ParT (S48C) ratio
1/128
1/64
1/32
1/16
1/8
1/4
1/2
1/1
2/1
X-linked ParT
ParT (S48C)
MW (kDa)
180
130
95
72
55
43
Crosslinked fraction (%)
60
40
20
0
parS/ParT (S48C) ratio
D
BMOE
parS
NTP
ATP
CTP
GTP
UTP
X-linked ParT
ParT(S48C)
MW (kDa)
250
180
130
95
72
55
43
Lane
1
2
3
4
5
6
Crosslinked fraction (%)
60
40
20
0
Lane

Fig. S9. The crosslinking of ParT (S48C) is not affected by NTP. (A) BLI analysis of the interaction between 1 μM ParT (S48C) with a 180-bp dual biotin-labeled *parS* closed DNA loop (red) or a BamHI-restricted loop (brown) to generate a free DNA end. BLI analysis indicated that ParT (S48C) could accumulate on a closed DNA loop but the accumulation was much reduced on a DNA with an open end. Mean and standard deviation (shading) from three replicates are shown. **(B)** SDS-PAGE analysis of BMOE crosslinking products of ParT (WT), ParT (S48C), and ParT (A68C) with or without 40-bp *parS* DNA duplex or scrambled *parS* DNA duplex. ParT (WT) with a native cysteine residue C81 crosslinked minimally in tested conditions (double arrows, minor X species). In additional negative control, ParT(A68C) variant, in which the opposing cysteines at position 68 were predicted to be 64 Å apart-too distant for the 8Å BMOE crosslinker, also crosslinked minimally, regardless of the presence of additional *parS* DNA. Most crosslinked products (single arrow, major X) appeared when a ParT (S48C) variant was employed instead. **(C)** Sub-stoichiometric concentrations of *parS* are sufficient to promote crosslinking of ParT (S48C). SDS-PAGE analysis of BMOE crosslinking products of 4 μM of KorB (S48C) dimer + increasing concentration of 40-bp *parS* DNA (from 1/128 to 2/1 *parS*-to-ParT molar ratio). A ratio of ~8-fold less *parS* DNA to ParT was sufficient to achieve maximal crosslinking. Data are represented as mean values ± SD from three replicates. **(D)** SDS-PAGE analysis of BMOE crosslinking products of 4 μM ParT (S48C) in the presence of 4 μM 40-bp *parS* DNA and 1 mM NTP. Experiments were performed in triplicates, and a quantification (mean ± SD) of the major crosslinked fraction is shown below the representative image.

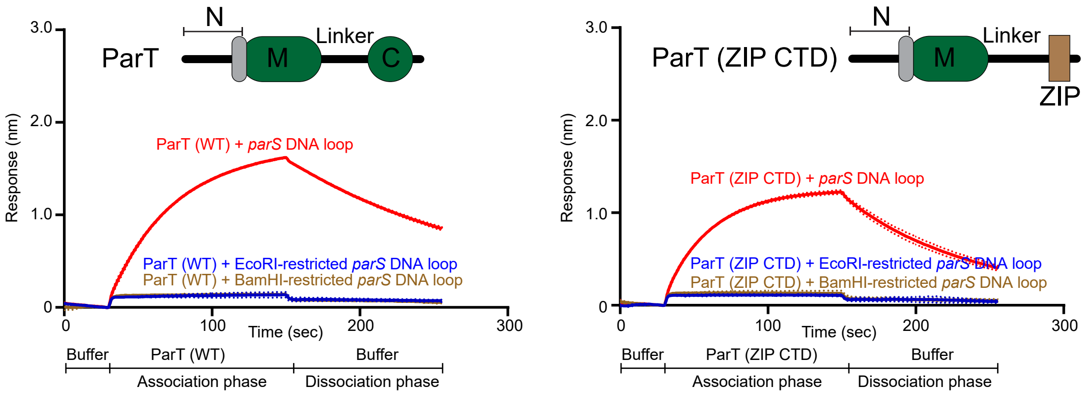

Fig. S10. ParT (ZIP CTD), in which the C domain has been substituted by a constitutive leucine zipper dimerization peptide, bound and accumulated on a closed *parS* DNA loop. BLI analysis of the interaction between 1 μM ParT (WT) (left panel) or ParT (ZIP CTD) (right panel) with a 180-bp dual biotin-labeled *parS* closed DNA loop (red) or a BamHI-restricted loop (brown) or an EcoRI-restricted loop (blue) to generate a free DNA end. Mean and standard deviation (shading) from three replicates are shown.

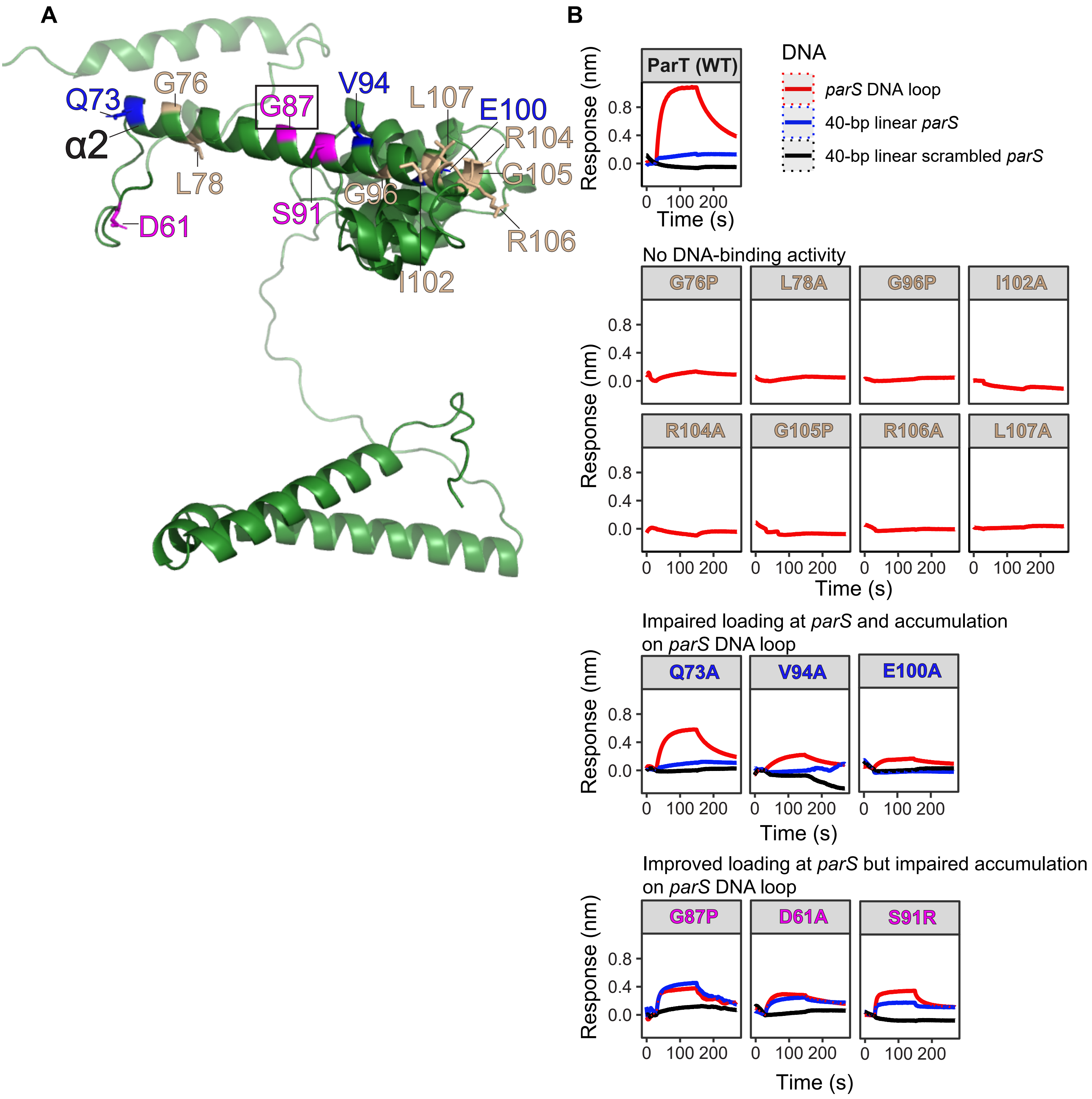

Fig. S11. Alanine and proline scanning mutagenesis of the N-domain helix α2 of ParT. (A) AlphaFold2-predicted structure of a ParT monomer with the positions of mutagenized residues indicated. **(B)** BLI analysis of the interaction between 1 μM ParT (WT or variants from panel A) with a 180-bp dual biotin-labeled *parS* closed DNA loop (red) or a 40-bp biotin-labeled linear *parS* DNA (blue) or a 40-bp biotin-labeled linear scrambled *parS* DNA (black).

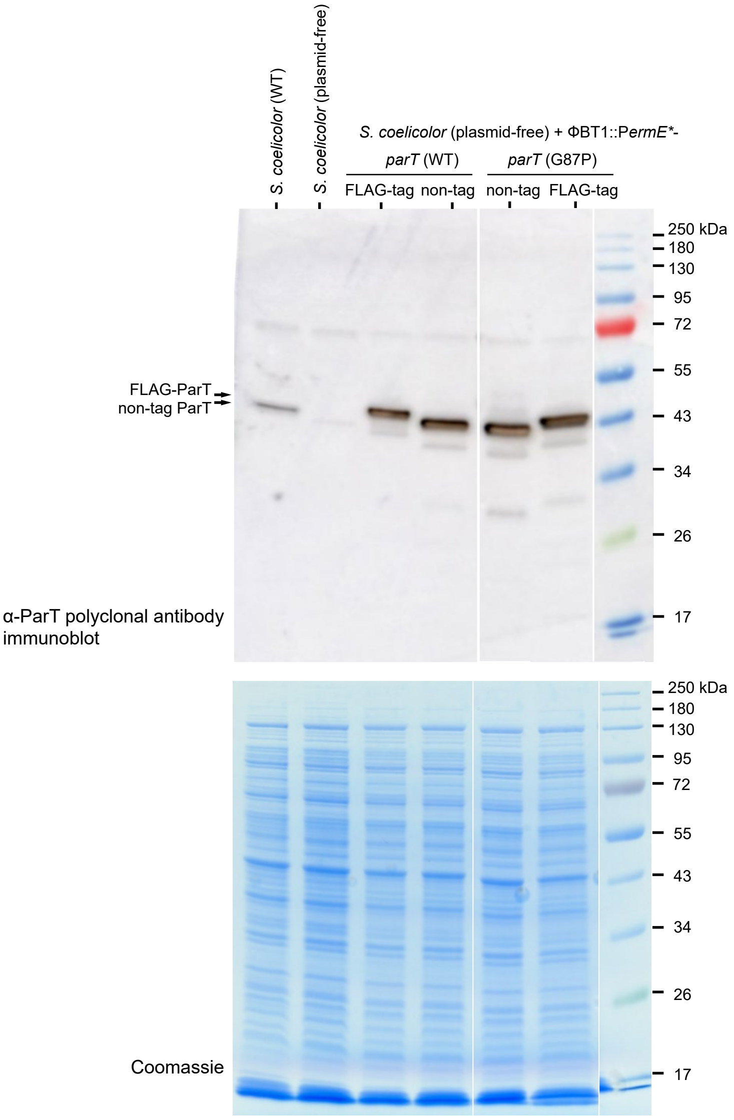

Fig. S12. ParT (WT) or ParT (G87P) were produced to the same level from the *ermE promoter in the plasmid-free *S. coelicolor* M600 strain.** Immunoblot of ParT (WT or variants) using a polyclonal antibody against purified ParT. Loading controls (Coomassie-stained SDS-PAGE) from lysates of cells used in the same experiments are shown below the immunoblot.

Table S3. Plasmids used in this study

Vector	Description	Source
pET21b	Protein overexpression vector, IPTG inducible T7 promoter, carbenicilin^R	Lab stock
pIJ10257	Plasmid for the conjugal transfer of DNA from *E. coli* to *Streptomyces* spp, cloned gene is expressed from the *ermE** promoter. Plasmid is integrated specifically at the ΦBT1 phage integration site on the *S. coelicolor* chromosome, hygromycin^R	(25)
pUZ8002	A helper plasmid for conjugative transfer of pIJ10257 from *E. coli* to *S. coelicolor*, kanamycin^R	(26)
pET21b::parT(WT)-his$_6$	Over-expression plasmid for ParT (WT), 6xHis-tagged at the C-terminus, carbenicilin^R	This study
pET21b::parA(WT)-his$_6$	Over-expression plasmid for ParA (WT), 6xHis-tagged at the C-terminus, carbenicilin^R	This study
pET21b::parT(S48C)-his$_6$	Over-expression plasmid for ParT (S48C), 6xHis-tagged at the C-terminus, carbenicilin^R	This study
pET21b::parT(Q271C)-his$_6$	Over-expression plasmid for ParT (Q271C), 6xHis-tagged at the C-terminus, carbenicilin^R	This study
pET21b::parT(A68C)-his$_6$	Over-expression plasmid for ParT (A68C), 6xHis-tagged at the C-terminus, carbenicilin^R	This study
pET21b::parT(G76P)-his$_6$	Over-expression plasmid for ParT (G76P), 6xHis-tagged at the C-terminus, carbenicilin^R	This study
pET21b::parT(L78A)-his$_6$	Over-expression plasmid for ParT (L78A), 6xHis-tagged at the C-terminus, carbenicilin^R	This study
pET21b::parT(G96P)-his$_6$	Over-expression plasmid for ParT (G96P), 6xHis-tagged at the C-terminus, carbenicilin^R	This study
pET21b::parT(I102A)-his$_6$	Over-expression plasmid for ParT (I102A), 6xHis-tagged at the C-terminus, carbenicilin^R	This study
pET21b::parT(R104A)-his$_6$	Over-expression plasmid for ParT (R104A), 6xHis-tagged at the C-terminus, carbenicilin^R	This study
pET21b::parT(G105P)-his$_6$	Over-expression plasmid for ParT (G105P), 6xHis-tagged at the C-terminus, carbenicilin^R	This study
pET21b::parT(R106A)-his$_6$	Over-expression plasmid for ParT (R106A), 6xHis-tagged at the C-terminus, carbenicilin^R	This study
pET21b::parT(L107A)-his$_6$	Over-expression plasmid for ParT (L107A), 6xHis-tagged at the C-terminus, carbenicilin^R	This study
pET21b::parT(Q73A)-his$_6$	Over-expression plasmid for ParT (Q73A), 6xHis-tagged at the C-terminus, carbenicilin^R	This study
pET21b::parT(V94A)-his$_6$	Over-expression plasmid for ParT (V94A), 6xHis-tagged at the C-terminus, carbenicilin^R	This study
pET21b::parT(E100A)-his$_6$	Over-expression plasmid for ParT (E100A), 6xHis-tagged at the C-terminus, carbenicilin^R	This study
pET21b::parT(G87P)-his$_6$	Over-expression plasmid for ParT (G87P), 6xHis-tagged at the C-terminus, carbenicilin^R	This study
pET21b::parT(D61A)-his$_6$	Over-expression plasmid for ParT (D61A), 6xHis-tagged at the C-terminus, carbenicilin^R	This study
pET21b::parT(S91R)-his$_6$	Over-expression plasmid for ParT (S91R), 6xHis-tagged at the C-terminus, carbenicilin^R	This study
pET21b::HALO-ParT	Over-expression plasmid for HALO-tagged ParT (WT), 6xHis-tagged at the C-terminus, carbenicilin^R	This study
pIJ10257::parT-flag	Integrative plasmid haboring a *parT* variant with a FLAG tag at the C-terminus, hygromycin^R	This study
pIJ10257::parT	Integrative plasmid harboring a non-tagged WT *parT*, hygromycin^R	This study
pSS88::parT-mcherry	Integrative plasmid haboring a *parT* variant with an mcherry tag at the C-terminus, hygromycin^R	This study
pUT18C::empty	Empty plasmid for bacterial two-hybrid assay, carbenicilin^R	Euromedex
pKT25::empty	Empty plasmid for bacterial two-hybrid assay, kanamycin^R	Euromedex
pUT18C::zip	Bacterial two-hybrid plasmid expressing a leucine zipper domain from GCN4, carbenicilin^R	Euromedex

pKT25::zip	Bacterial two-hybrid plasmid expressing a leucine zipper domain from GCN4, kanamycin^R	Euromedex
pUT18C-N domain α2	Bacterial two-hybrid plasmid expressing the N-domain of ParT, carbenicilin^R	This study
pUT18C-C domain	Bacterial two-hybrid plasmid expressing the C-domain of ParT, carbenicilin^R	This study
pKT25-N domain α2	Bacterial two-hybrid plasmid expressing the N-domain of ParT, kanamycin^R	This study
pKT25-C domain	Bacterial two-hybrid plasmid expressing the C-domain of ParT, kanamycin^R	This study
p64.large plasmid backbone	Large plasmid fabricated as described in (12) derived from pNLrep (27) employed as backbone to easily fabricate new large plasmids for C-Trap experiments. Also here employed for C-Trap experiments, ampicillin^R	Lab stock
p145A.1x parS	Large plasmid derived from p64.large plasmid backbone containing 1x parS employed in additional clonings but also useful for C-Trap experiments, ampicillin^R	This study
p145B.3x parS	Large plasmid derived from p145A.1x parS containing 3x parS for C-Trap experiments, ampicillin^R	This study
pUC19_v2	Enlarged pUC19 plasmid fabricated in (13), ampicillin^R	Lab stock
p144A.1x parS	Plasmid derived from pUC19_v2 containing 1x parS employed in additional clonings but also useful for magnetic tweezers experiments, ampicillin^R	This study
p144B.3x parS	Plasmid derived from p144A.1x parS containing 3x parS employed in additional clonings but also useful for magnetic tweezers experiments, ampicillinR	This study
p144C.5x parS	Plasmid derived from p144B.3x parS containing 5x parS for magnetic tweezers experiments, ampicillin^R	This study
pSP73-JY0	Plasmid employed as template for PCR amplification to generate the handles for magnetic tweezers and C-Trap experiments, ampicillin^R	Lab stock, (14)

Table S4. Strains used in this study

**Strain**	**Use**	**Source**
E. coli DH5α	A cloning strain for the plasmids in this study	Lab stock
E. coli Rosetta (DE3) pLys	A host strain for protein over-expression plasmids	Lab stock
BTH101	*cya- E. coli* host for bacterial-two hybrid assay	Euromedex
Sreptomyces coelicolor A3(2)	WT *S. coelicolor* A3(2), which was used as a recipient for *parT* variants, SCP1$^+$ SCP2$^+$	Lab stock
Sreptomyces coelicolor M600	Plasmid-free *S. coelicolor* M600, which was used as a recipient for *parT* variants, SCP1$^-$ SCP2$^-$	Lab stock
E. coli Rosetta (DE3) pLys pET21b::parT-his$_6$	Over-expression of ParT (WT) 6xHis	This study
E. coli Rosetta (DE3) pLys pET21b::parA-his$_6$	Over-expression of ParA (WT) 6xHis	This study
E. coli Rosetta (DE3) pLys pET21b::parT (S48C)-his$_6$	Over-expression of ParT (S48C) 6xHis	This study
E. coli Rosetta (DE3) pLys pET21b::parT (Q271C)-his$_6$	Over-expression of ParT (Q271C) 6xHis	This study
E. coli Rosetta (DE3) pLys pET21b::parT(A68C)-his$_6$	Over-expression of ParT (A68C) 6xHis	This study
E. coli Rosetta (DE3) pLys pET21b::parT(G76P)-his$_6$	Over-expression of ParT (G76P) 6xHis	This study
E. coli Rosetta (DE3) pLys pET21b::parT(L78A)-his$_6$	Over-expression of ParT (L78A) 6xHis	This study
E. coli Rosetta (DE3) pLys pET21b::parT(G96P)-his$_6$	Over-expression of ParT (G96P) 6xHis	This study
E. coli Rosetta (DE3) pLys pET21b::parT(I102A)-his$_6$	Over-expression of ParT (I102A) 6xHis	This study

E. coli Rosetta (DE3) pLys pET21b::parT(R104A)-his$_6$	Over-expression of ParT (R104A) 6xHis	This study
E. coli Rosetta (DE3) pLys pET21b::parT(G105P)-his$_6$	Over-expression of ParT (G105P) 6xHis	This study
E. coli Rosetta (DE3) pLys pET21b::parT(R106A)-his$_6$	Over-expression of ParT (R106A) 6xHis	This study
E. coli Rosetta (DE3) pLys pET21b::parT(L107A)-his$_6$	Over-expression of ParT (L107A) 6xHis	This study
E. coli Rosetta (DE3) pLys pET21b::parT(Q73A)-his$_6$	Over-expression of ParT (Q73A) 6xHis	This study
E. coli Rosetta (DE3) pLys pET21b::parT(V94A)-his$_6$	Over-expression of ParT (V94A) 6xHis	This study
E. coli Rosetta (DE3) pLys pET21b::parT(E100A)-his$_6$	Over-expression of ParT (E100A) 6xHis	This study
E. coli Rosetta (DE3) pLys pET21b::parT(G87P)-his$_6$	Over-expression of ParT (G87P) 6xHis	This study
E. coli Rosetta (DE3) pLys pET21b::parT(D61A)-his$_6$	Over-expression of ParT (D61A) 6xHis	This study
E. coli Rosetta (DE3) pLys pET21b::parT(S91R)-his$_6$	Over-expression of ParT (S91R) 6xHis	This study
E. coli Rosetta (DE3) pLys pET21b::HALO-parT	Over-expression of HALO-tagged ParT (WT) 6xHis	This study
E. coli ET12567 + pUZ8002	*E. coli* strain carrying a conjugative helper plasmid pUZ8002	(26)
E. coli ET12567 pIJ10257::parT-flag + pUZ8002	Conjugative transfer from *E. coli* to *S. coelicolor* A3(2), to introduce a FLAG-tagged *parT* variant at the ΦBT1 phage integration site	This study
E. coli ET12567 pIJ10257::parT + pUZ8002	Conjugative transfer from *E. coli* to *S. coelicolor* A3(2), to introduce a WT *parT* variant at the ΦBT1 phage integration site	This study
S. coelicolor A3(2) ΦBT1*::parT-flag*	ChIP-seq experiment using anti-FLAG antibody	This study
S. coelicolor A3(2) ΦBT1*::parT*	ChIP-seq experiment (negative control) using anti-FLAG antibody	This study
S. coelicolor A3(2) ΦBT1*::parT-mcherry*	Microscopy imaging of mCherry-tagged ParT in *S. coelicolor* A3(2)	This study
S. coelicolor M600 ΦBT1*::parT-mcherry*	Microscopy imaging of mCherry-tagged ParT in *S. coelicolor* M600	This study
BTH101 pUT18C-N domain α2 + pKT25-N domain α2	Bacterial two-hybrid assays	This study
BTH101 pUT18C::empty + pKT25-N domain α2	Bacterial two-hybrid assays	This study
BTH101 pUT18C-N domain α2 + pKT25::empty	Bacterial two-hybrid assays	This study
BTH101 pUT18C-C domain + pKT25-C domain	Bacterial two-hybrid assays	This study
BTH101 pUT18C-zip + pKT25-zip	Bacterial two-hybrid assays	This study

Table S5. DNA oligonucleotides used in this study

**Name**	**Sequence**
parS_Fw_bio	5’BIOTIN-GGCCGACCTCGTGTCTCCAATTGGAGACATCAACGAGGGC
parS_Rv	GCCCTCGTTGATGTCTCCAATTGGAGACACGAGGTCGGCC
scram parS_Fw	5’BIOTIN-ATCGGGCACCTTAGGGCAATCGTAAGTTCGGCGCACGACC
scram parS_Rv	GGTCGTGCGCCGAACTTACGATTGCCCTAAGGTGCCCGAT
ParT_CCherry_Fw	GTTGGTAGGATCGTCTAGAACAGGAGGCCCCATATGAGCCGCCGCTCCCTCGCCCTCCC
ParT_par_disr_Rv	GTTGATGTCGCCGATCGGGCTGACGAGGTCGGCCGGTCGCCACCCCTCGATC

ParT_par_disr_Fw	CTCGTCAGCCCGATCGGCGACATCAACGAGGGCCAGGCCCGCGAGCTG	
ParT_CCherry_Rv	GTCCTCCTCGCCCTTGGAGACCATCTCGAGCGCGTCGTCGGCCACCTGCTCACCG GAGG	
M13F-biotin	5'BIOTIN-CGCCAGGGTTTTCCCAGTCACGAC	
M13R-biotin	5'BIOTIN-AGGAAACAGCTATGACCAT	
ParT_CFLAG_Fw	GATCGTCTAGAACAGGAGGCCCCATATGAGCCGCCGCTCCCTCGCCCTC	
ParT_CFLAG_Rv	TCCGCTCATGAGAACCTAGGATCCATTACTTGTCGTCATCGTCCTTGTAGTCGCCC GCGTCGTCGGCCACCTGCTCACCG	
ParT_Fw	GTGAGCCGCCGCTCCCTCGCCCTCCCGTCG	
ParT_Rv	TTACGCGTCGTCGGCCACCTGCTCACCGGAG	
Annealed oligonucleotides with 1x *parS* site	342.R-1xparS-ParT	GCGTCGCCCTCGTTGATGTCTCCAATTGGAGACAC GAGGTCGGCCGGTAC
	347.F-1xparS-ParT ctrap	CGGCCGACCTCGTGTCTCCAATTGGAGACATCAAC GAGGGCGACGCGTAC
Annealed oligonucleotides with 2x *parS* site	343.F-2parS dianas	TGAGCTCGAGGCGCCCGTGTCTCCAATTGGAGACA TCAAGTATAGAGCACCTGTTACGTACCTAGTCAAGG ACTCAGCGTGTCTCCAATTGGAGACATCGTTAACGT CGACGC
	344.R-2parS dianas	TCAGCGTCGACGTTAACGATGTCTCCAATTGGAGA CACGCTGAGTCCTTGACTAGGTACGTAACAGGTGC TCTATACTTGATGTCTCCAATTGGAGACACGGGCGC CTCGAGC
MT 1x *parS* PCR fragment	329.PCR_template_T7pCD NA3_FW	ATCGAAATTAATACGACTCACTATAGG
	325.R req lambda 4	GCCCGGATTCAAATGCTGCAG
MT DIG handle	57.FMH_F2_BamHI-ApaI	GCGTAAGTGGATCCGGGCCCCGACTCACTATAGGG AGAC<br>CGGC
	JOE_R1	AGTAAGCGCCGTCAGACCAG
MT BIO handle	57.FMH_F2_BamHI-ApaI	GCGTAAGTGGATCCGGGCCCCGACTCACTATAGGG AGAC<br>CGGC
	209.BsrGI 71short handle	CGATAACCAACTGGCGATG
C-trap BIO handle	57.FMH_F2_BamHI-ApaI	GCGTAAGTGGATCCGGGCCCCGACTCACTATAGGG AGACCGGC
	JOE_R1	AGTAAGCGCCGTCAGACCAG

Table S6. List of ChIP-seq data used in this study

ChIP-seq samples	Genetic backgrounds	antibody	replicates	Source
sample 1	*S. coelicolor A3(2)* ΦBT1*::parT*	anti-FLAG	replicate 1	This study
sample 2	*S. coelicolor A3(2)* ΦBT1*::parT-flag*	anti-FLAG	replicate 1	This study
sample 3	*S. coelicolor A3(2)* ΦBT1*::parT*	anti-FLAG	replicate 2	This study

sample 4	*S. coelicolor A3(2)* ΦBT1*::parT-flag*	anti-FLAG	replicate 2	This study
sample 5	*S. coelicolor M600* ΦBT1*::parT*	anti-FLAG	replicate 1	This study
sample 6	*S. coelicolor M600* ΦBT1*::parT-flag*	anti-FLAG	replicate 1	This study
sample 7	*S. coelicolor M600* ΦBT1*::parT*	anti-FLAG	replicate 2	This study
sample 8	*S. coelicolor M600* ΦBT1*::parT-flag*	anti-FLAG	replicate 2	This study
sample 9	*S. coelicolor M600* ΦBT1*::parT (G87P)-flag*	anti-FLAG	replicate 1	This study
sample 10	*S. coelicolor M600* ΦBT1*::parT (G87P)-flag*	anti-FLAG	replicate 2	This study

REFERENCES

1. Kieser, Tobias, Bibb, Mervyn J, Buttner, Mark J, Chater, Keith F, Hopwood, David A, "Introduction of DNA into *Streptomyces*" in *Practical Streptomyces Genetics*, (Crowes, 2000), pp. 229–253.

2. C. E. Stevenson, *et al.*, Investigation of DNA sequence recognition by a streptomycete MarR family transcriptional regulator through surface plasmon resonance and X-ray crystallography. *Nucleic Acids Res.* **41**, 7009–22 (2013).

3. D. G. Myszka, Improving biosensor analysis. *J. Mol. Recognit.* **12**, 279–284 (1999).

4. B. Langmead, C. Trapnell, M. Pop, S. L. Salzberg, Ultrafast and memory-efficient alignment of short DNA sequences to the human genome. *Genome Biol.* **10**, R25 (2009).

5. A. R. Quinlan, I. M. Hall, BEDTools: a flexible suite of utilities for comparing genomic features. *Bioinformatics* **26**, 841–842 (2010).

6. UniProt Consortium, UniProt: the Universal Protein Knowledgebase in 2023. *Nucleic Acids Res.* **51**, D523–D531 (2023).

7. M. Varadi, *et al.*, AlphaFold Protein Structure Database: massively expanding the structural coverage of protein-sequence space with high-accuracy models. *Nucleic Acids Res.* **50**, D439–D444 (2022).

8. S. F. Altschul, *et al.*, Gapped BLAST and PSI-BLAST: a new generation of protein database search programs. *Nucleic Acids Res.* **25**, 3389–3402 (1997).

9. A. Stamatakis, RAxML version 8: a tool for phylogenetic analysis and post-analysis of large phylogenies. *Bioinformatics* **30**, 1312–1313 (2014).

10. M. A. Miller, *et al.*, A RESTful API for access to phylogenetic tools via the CIPRES science gateway. *Evol. Bioinform. Online* **11**, 43–48 (2015).

11. I. Letunic, P. Bork, Interactive tree of life (iTOL) v3: an online tool for the display and annotation of phylogenetic and other trees. *Nucleic Acids Res.* **44**, W242-245 (2016).

12. C. Aicart-Ramos, S. Hormeno, O. J. Wilkinson, M. S. Dillingham, F. Moreno-Herrero, Long DNA constructs to study helicases and nucleic acid translocases using optical tweezers. *Methods Enzymol.* **673**, 311–358 (2022).

13. T. C. McLean, *et al.*, KorB switching from DNA-sliding clamp to repressor mediates long-range gene silencing in a multi-drug resistance plasmid. *Nat. Microbiol.* 1–20 (2025). https://doi.org/10.1038/s41564-024-01915-3.

14. N. Fili, *et al.*, Visualizing helicases unwinding DNA at the single molecule level. *Nucleic Acids Res.* **38**, 4448–4457 (2010).

15. M. Pabst, *et al.*, Nucleotide and nucleotide sugar analysis by liquid chromatography-electrospray ionization-mass spectrometry on surface-conditioned porous graphitic carbon. *Anal. Chem.* **82**, 9782–9788 (2010).

16. M. Rejzek, *et al.*, Profiling of Sugar Nucleotides. *Methods Enzymol.* **597**, 209–238 (2017).

17. F. de A. Balaguer, *et al.*, CTP promotes efficient ParB-dependent DNA condensation by facilitating one-dimensional diffusion from parS. *eLife* **10**, e67554 (2021).

18. C. L. Pastrana, *et al.*, Force and twist dependence of RepC nicking activity on torsionally-constrained DNA molecules. *Nucleic Acids Res.* **44**, 8885–8896 (2016).

19. T. R. Strick, J. F. Allemand, D. Bensimon, V. Croquette, Behavior of supercoiled DNA. *Biophys. J.* **74**, 2016–2028 (1998).

20. T. Strick, J. Allemand, V. Croquette, D. Bensimon, Twisting and stretching single DNA molecules. *Prog. Biophys. Mol. Biol.* **74**, 115–140 (2000).

21. J. Gorman, E. C. Greene, Visualizing one-dimensional diffusion of proteins along DNA. *Nat. Struct. Mol. Biol.* **15**, 768–774 (2008).

22. I. Heller, *et al.*, STED nanoscopy combined with optical tweezers reveals protein dynamics on densely covered DNA. *Nat. Methods* **10**, 910–916 (2013).

23. A. S. B. Jalal, *et al.*, CTP regulates membrane-binding activity of the nucleoid occlusion protein Noc. *Mol. Cell* **81**, 3623-3636.e6 (2021).

24. G. L. Fisher, *et al.*, The structural basis for dynamic DNA binding and bridging interactions which condense the bacterial centromere. *Elife* **6** (2017).

25. H.-J. Hong, M. I. Hutchings, L. M. Hill, M. J. Buttner, The role of the novel Fem protein VanK in vancomycin resistance in Streptomyces coelicolor. *J. Biol. Chem.* **280**, 13055–13061 (2005).

26. M. S. B. Paget, L. Chamberlin, A. Atrih, S. J. Foster, M. J. Buttner, Evidence that the extracytoplasmic function sigma factor E is required for normal cell wall structure in Streptomyces coelicolor A3(2). *J. Bacteriol.* **181**, 204–211 (1999).

27. N. Luzzietti, *et al.*, Efficient preparation of internally modified single-molecule constructs using nicking enzymes. *Nucleic Acids Res.* **39**, e15 (2011).
