# Supplementary material for: Expanding the diversity of bacterial DNA partitioning: A CTP-independent ParABS system for plasmid partitioning in Streptomyces
Source: Proc Natl Acad Sci U S A. 2025 Jul 2;122(27):e2406398122. doi: 10.1073/pnas.2406398122 (PMC12260392; doi:10.1073/pnas.2406398122)
Supplement: Supplementary file 2 — Dataset S01 (PDF) [file pnas.2406398122.sd01.pdf]

magenta: helix 2
green: ParT homolog
blue: *S. coelicolor* ParT

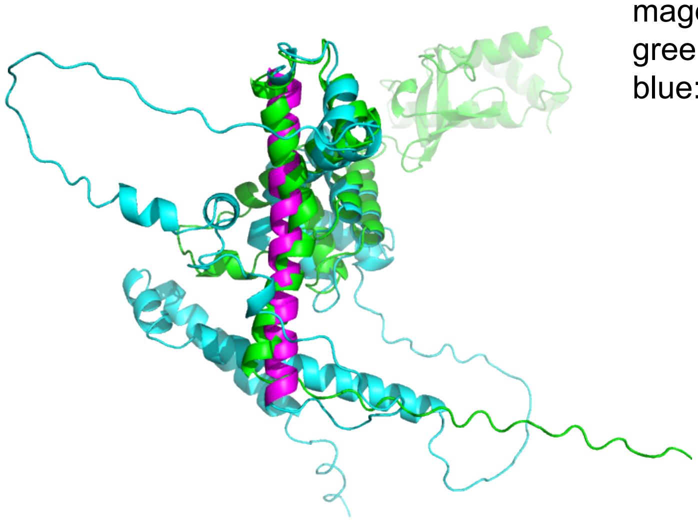

ACB54283_aligned_scene

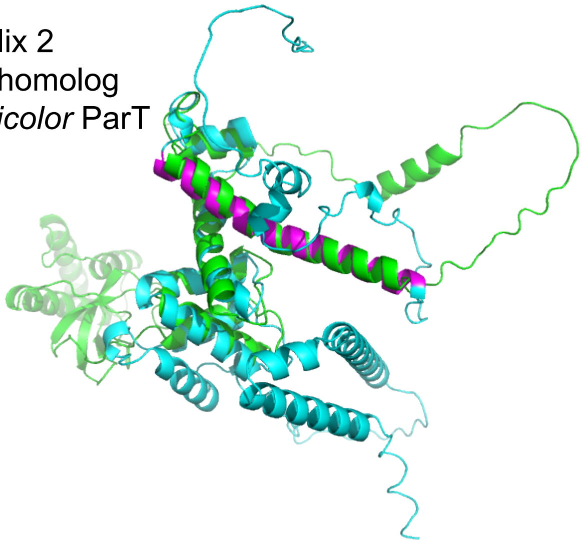

ACK73771_aligned_scene

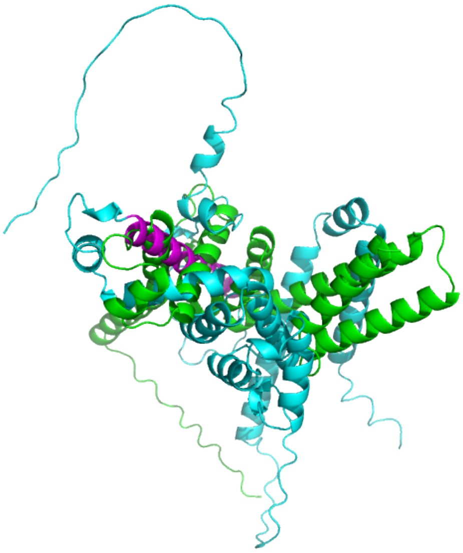

ACL05267_aligned_scene

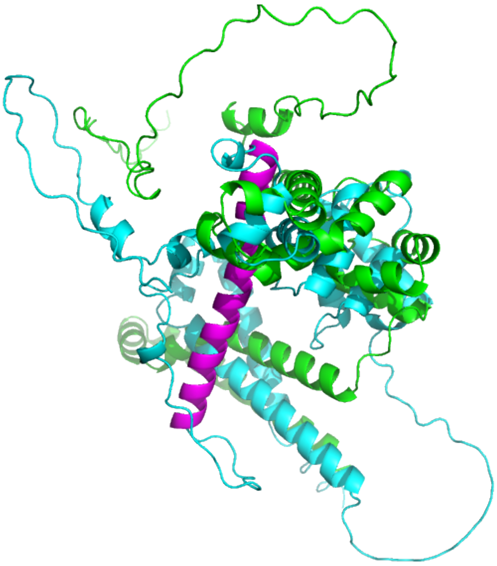

ACZ92024_aligned_scene

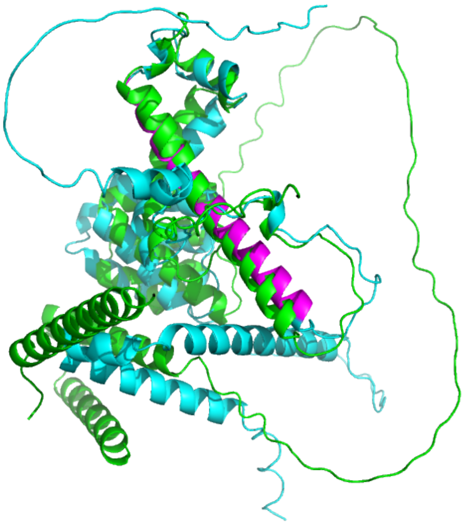

AEY94349_aligned_scene

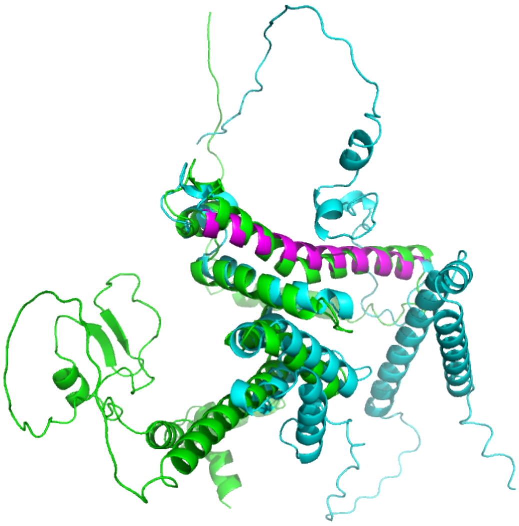

AFZ10710_aligned_scene

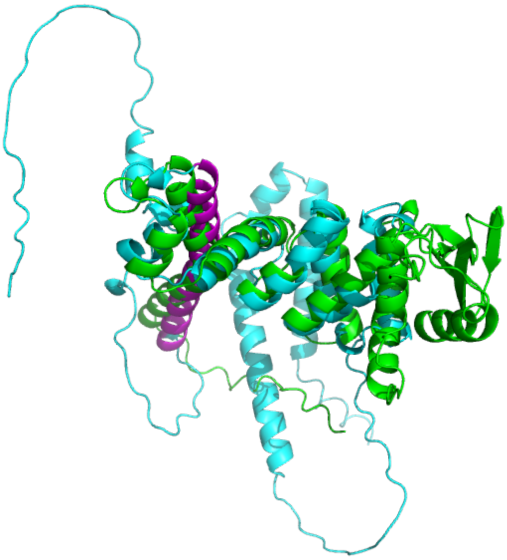

AFZ67101_aligned_scene

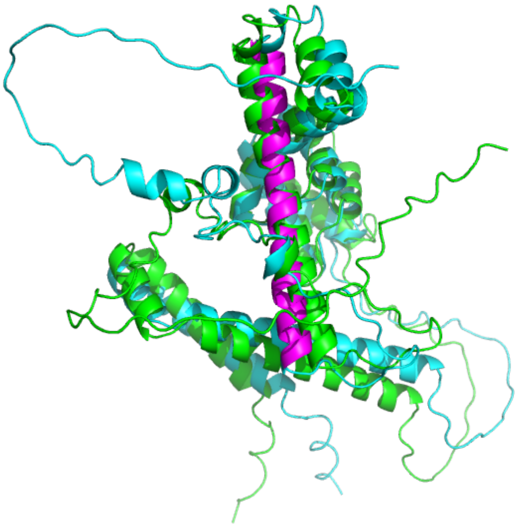

AGJ59499_aligned_scene

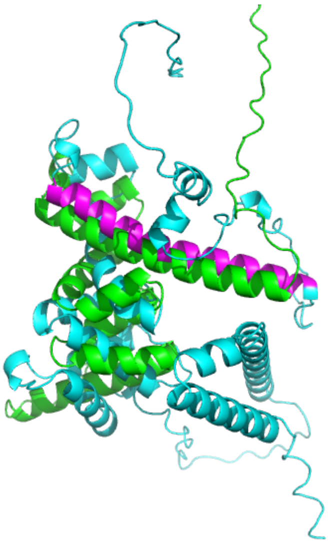

ASJ72247_aligned_scene

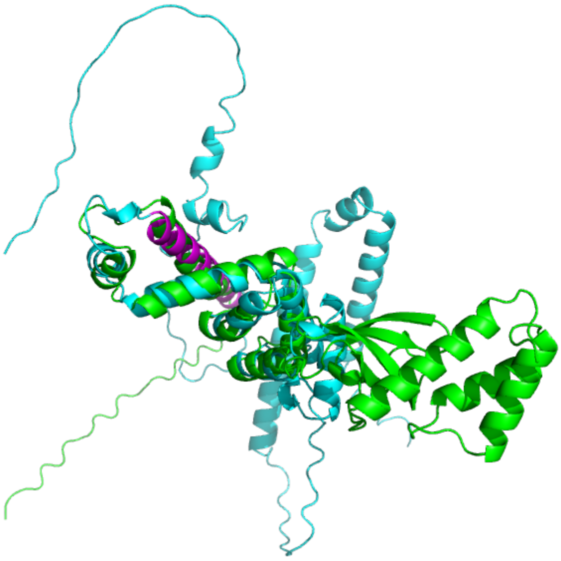

BAG03246_aligned_scene

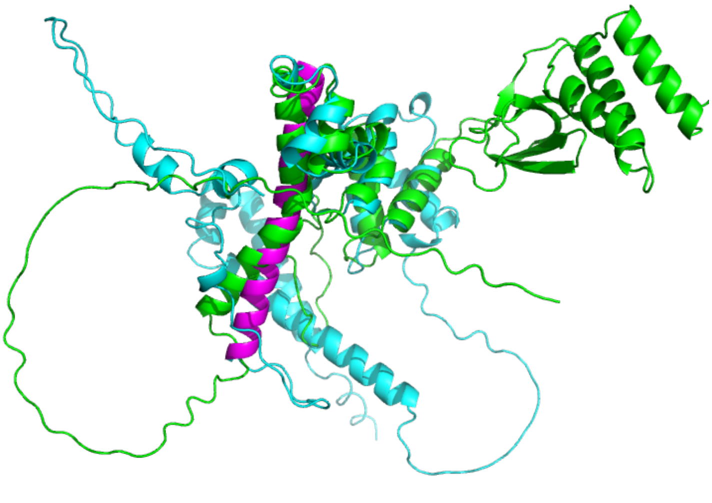

BAY20069_aligned_scene

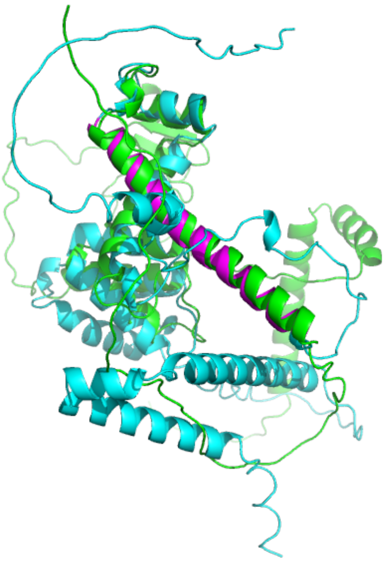

BAY35543_aligned_scene

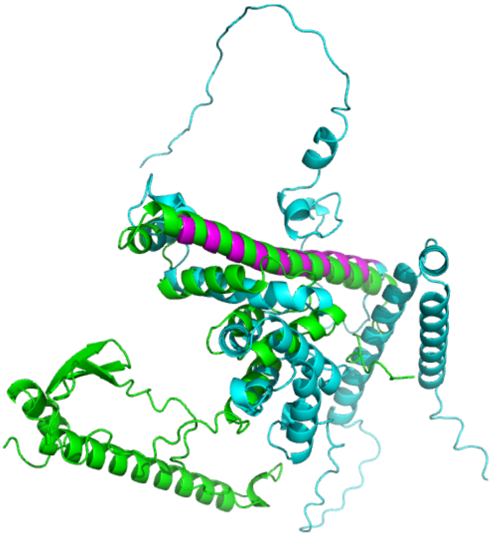

BAY55808_aligned_scene

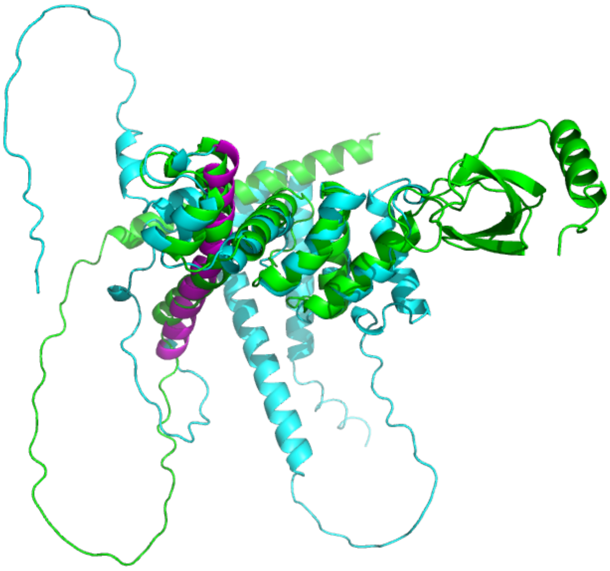

BAY72894_aligned_scene

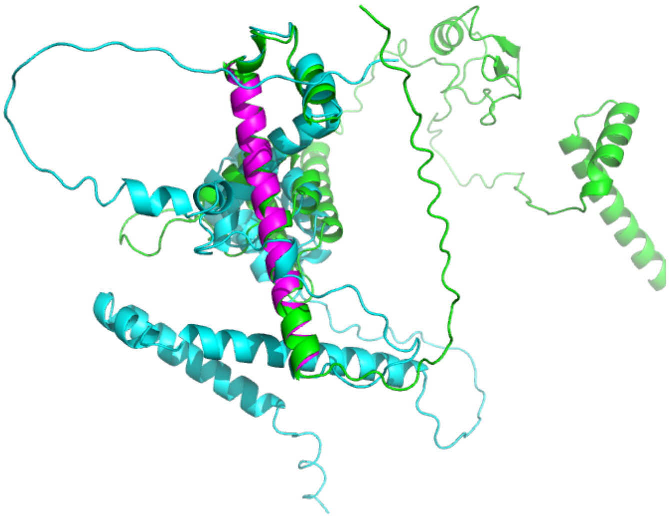

BAZ09198_aligned_scene

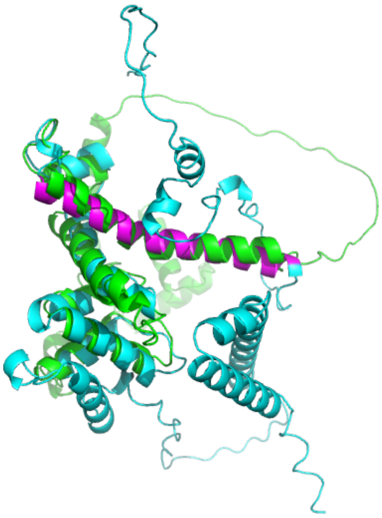

BAZ46978_aligned_scene

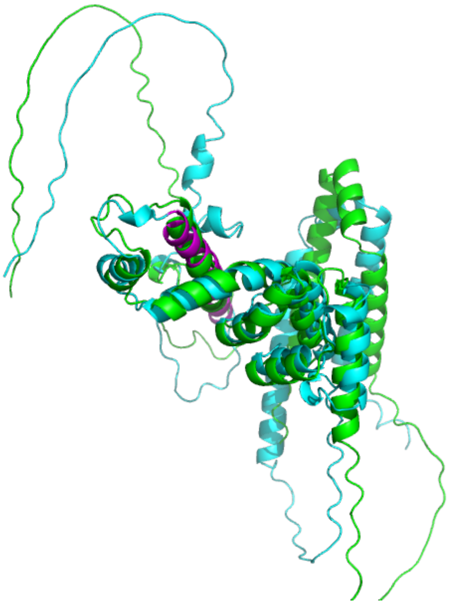

BBG20724_aligned_scene

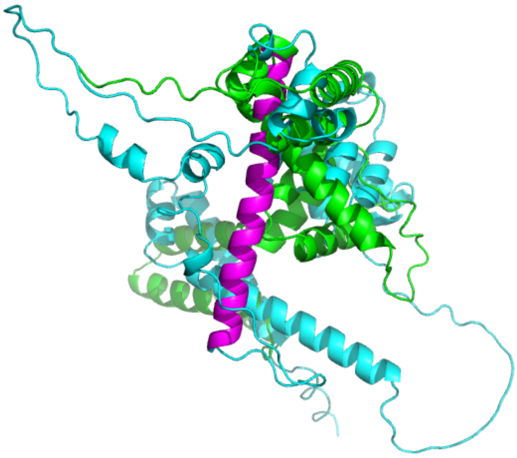

BBO74368_aligned_scene

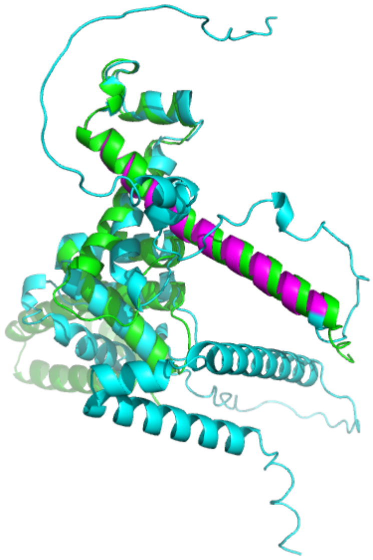

BBO74437_aligned_scene

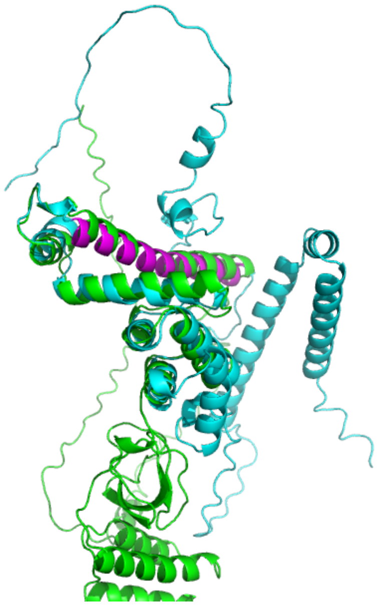

CCQ49684_aligned_scene

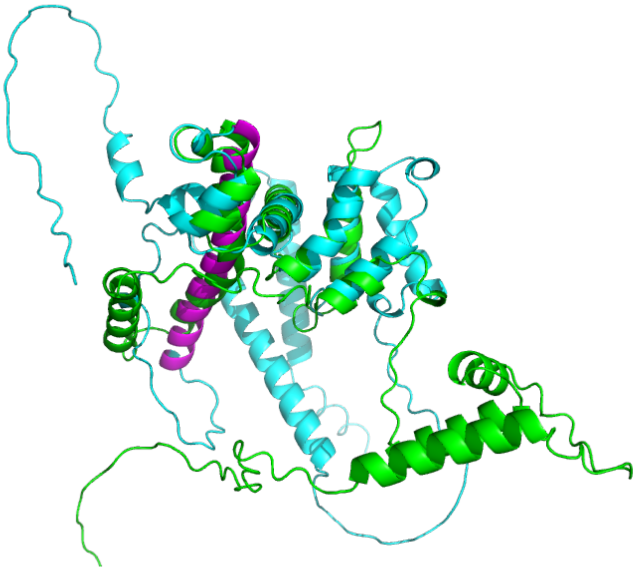

EGK89269_aligned_scene

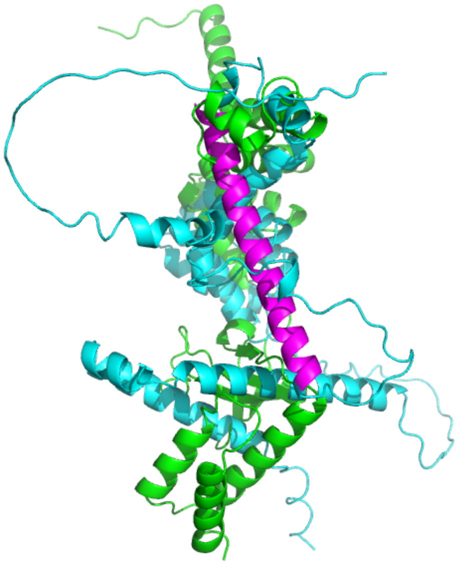

ELS00351_aligned_scene

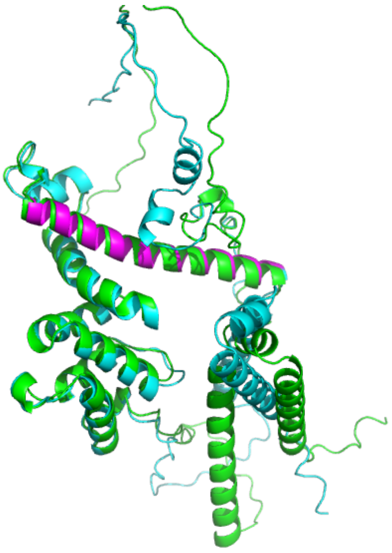

GAA0925100_aligned_scene

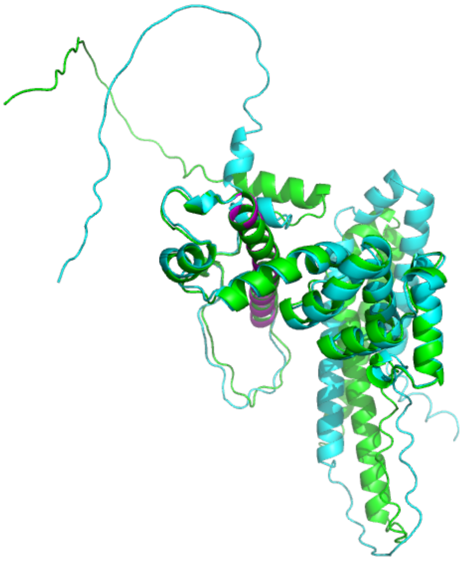

GAA1016867_aligned_scene

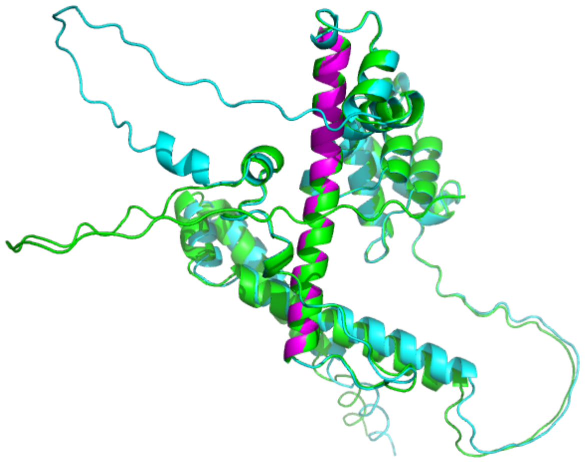

GAA2704711_aligned_scene

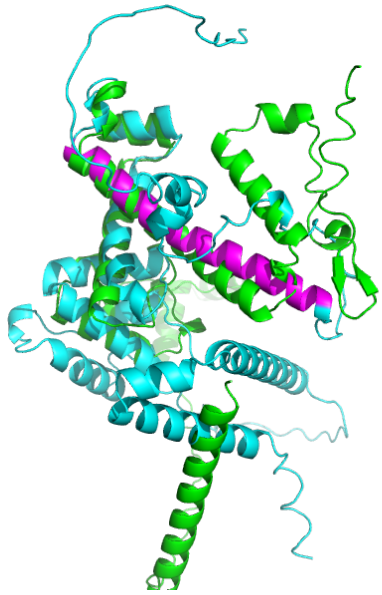

GBF79710_aligned_scene

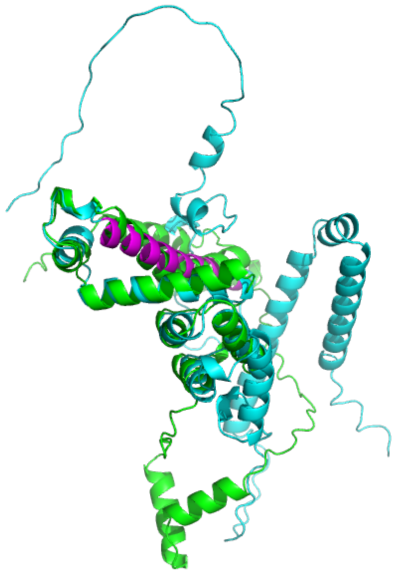

GBF82962_aligned_scene

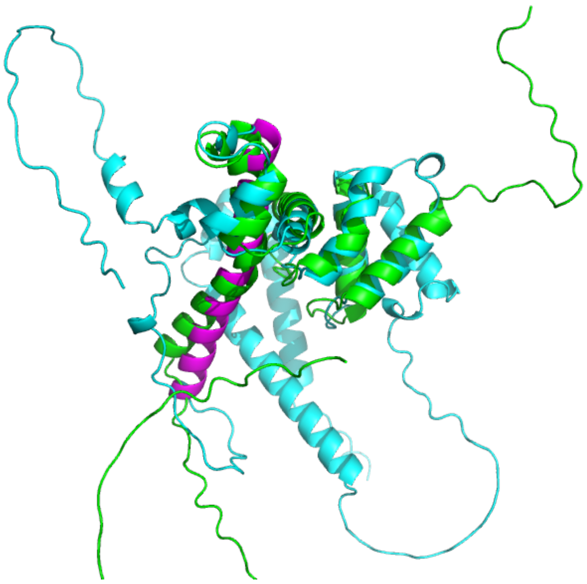

GBG23367_aligned_scene

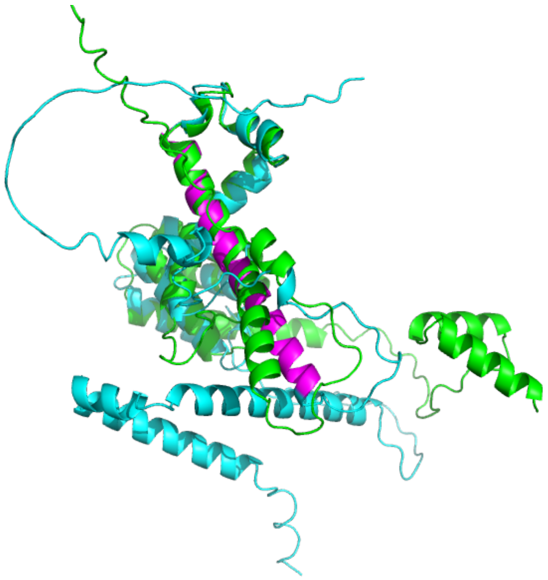

GDY60603_aligned_scene

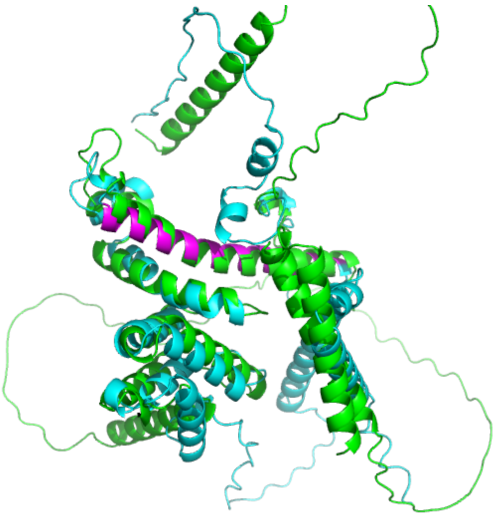

KIZ15626_aligned_scene

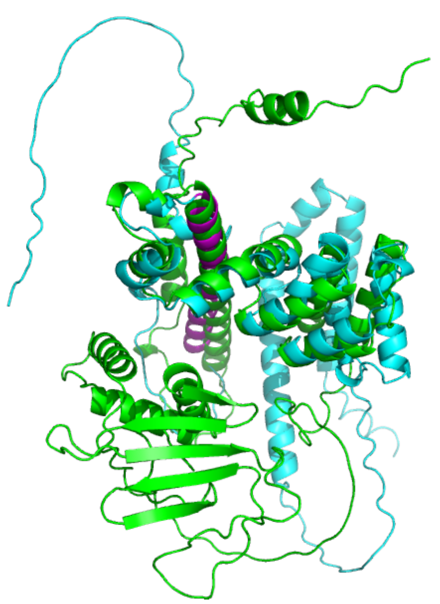

KOG85999_aligned_scene

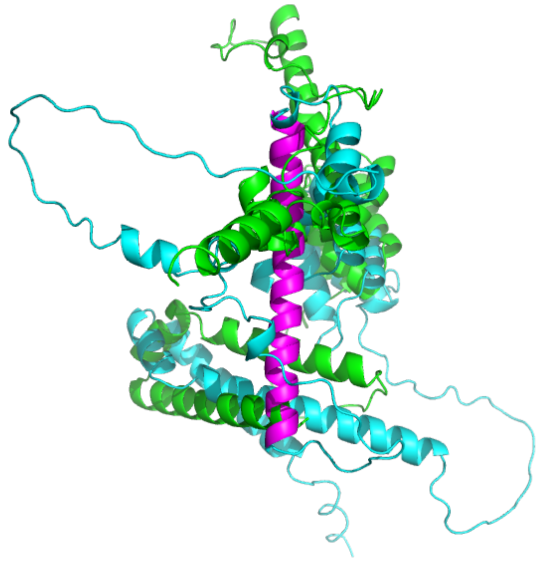

MBB0232181_aligned_scene

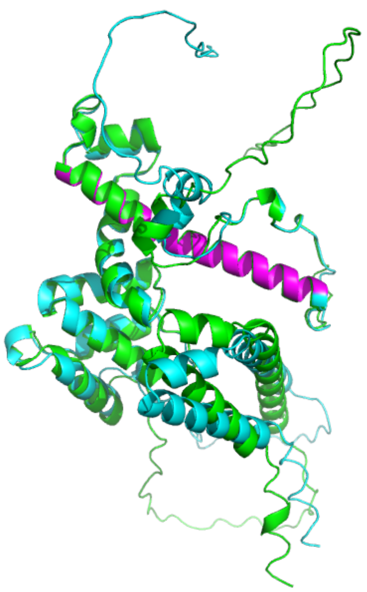

MBZ6086596_aligned_scene

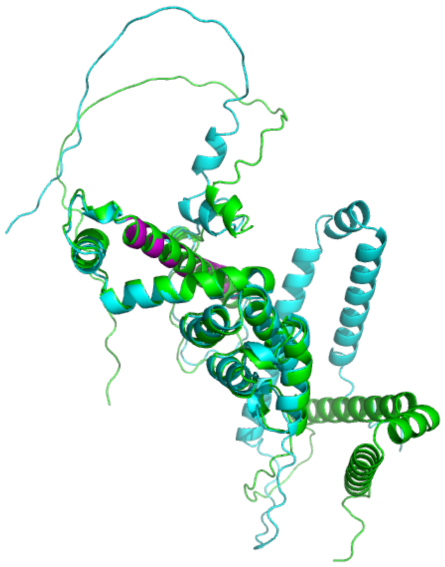

MDC2951048_aligned_scene

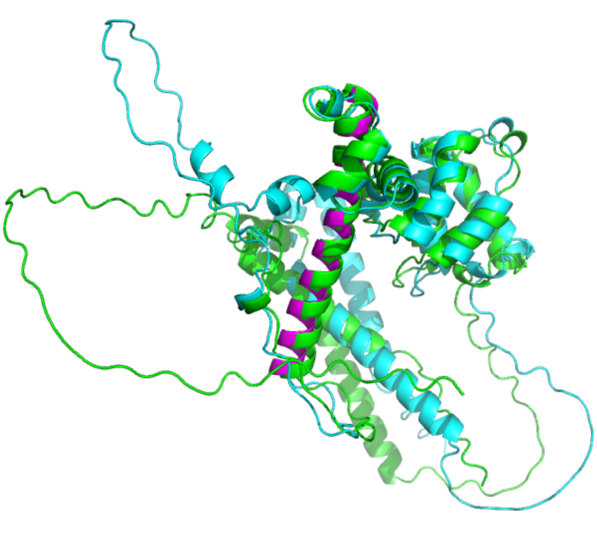

MDV9190951_aligned_scene

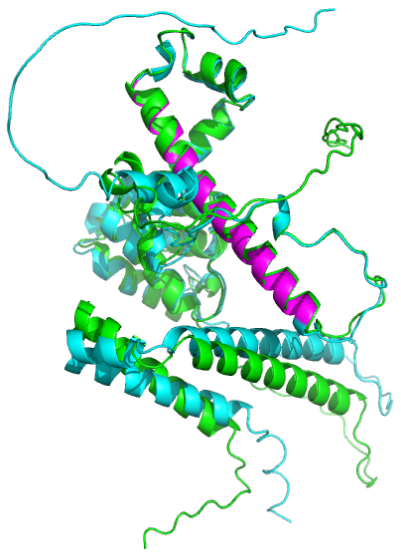

MEF9908618_aligned_scene

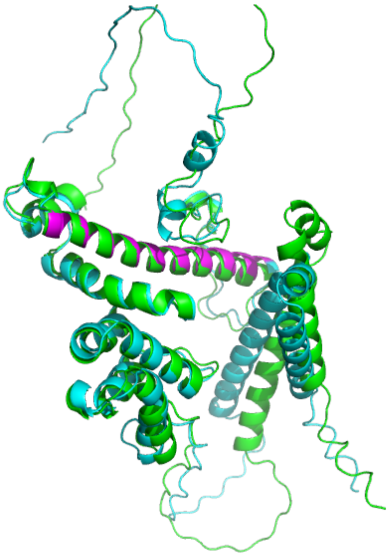

MEQ6028202_aligned_scene

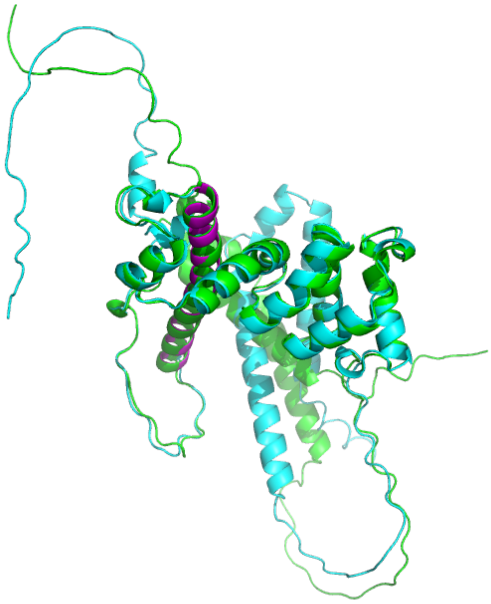

MER5555953_aligned_scene

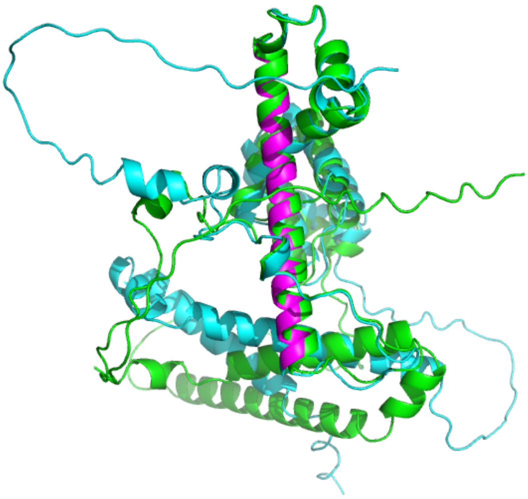

MER6789504_aligned_scene

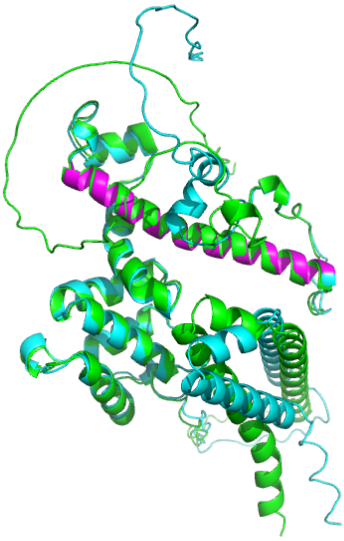

MET8221312_aligned_scene

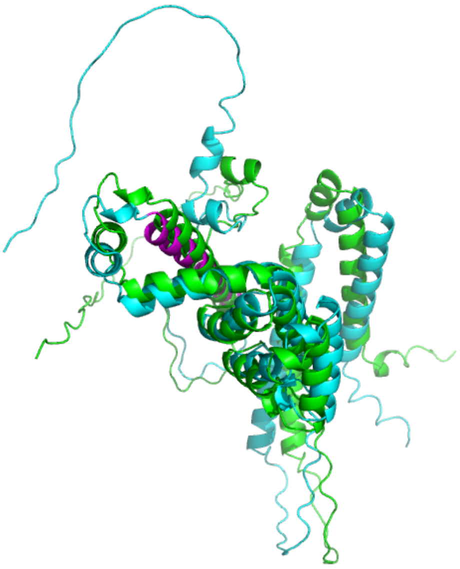

MEU1216650_aligned_scene

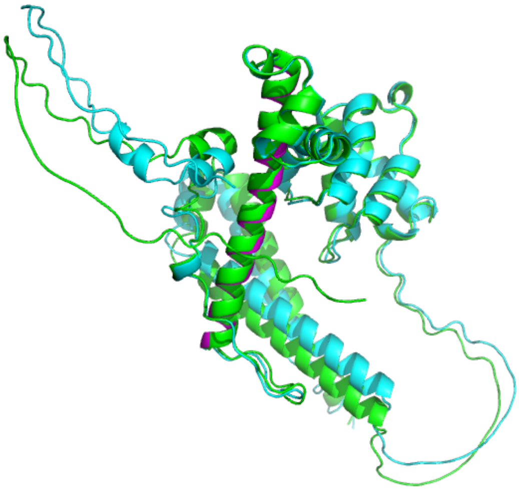

MEU2757282_aligned_scene

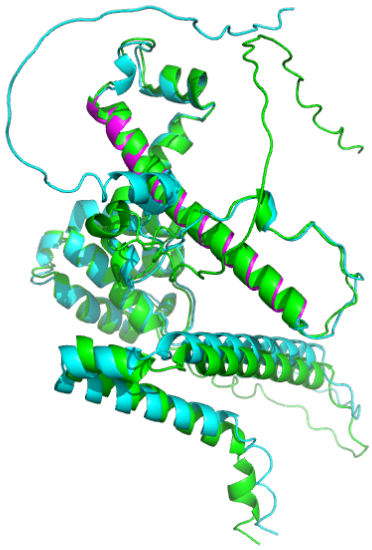

MEU3384097_aligned_scene

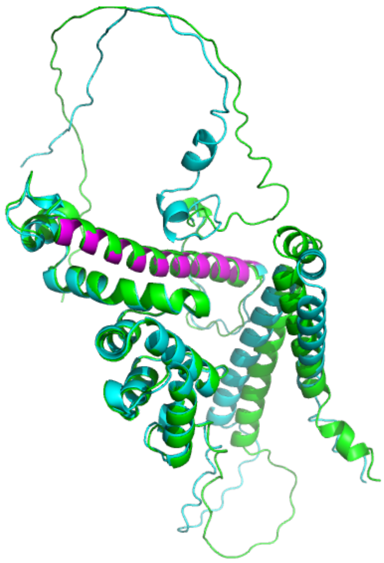

MEV5308791_aligned_scene

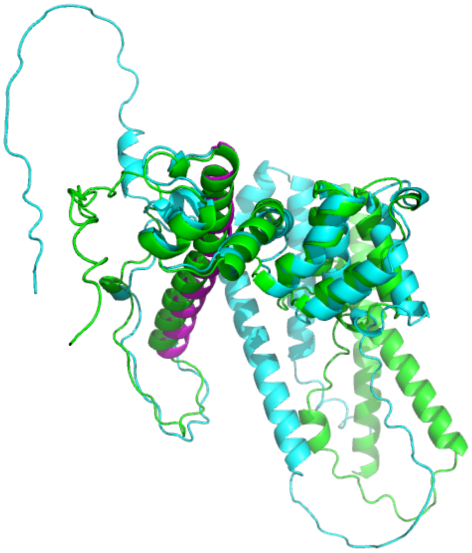

MEW1604972_aligned_scene

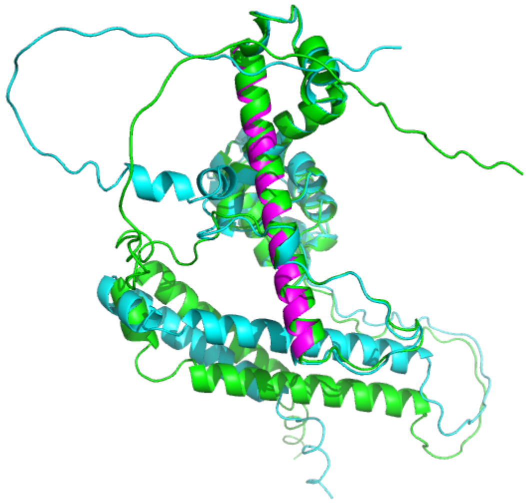

MEW2553669_aligned_scene

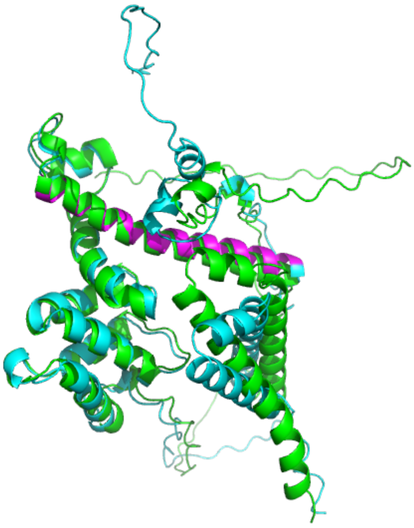

MFC5170652_aligned_scene

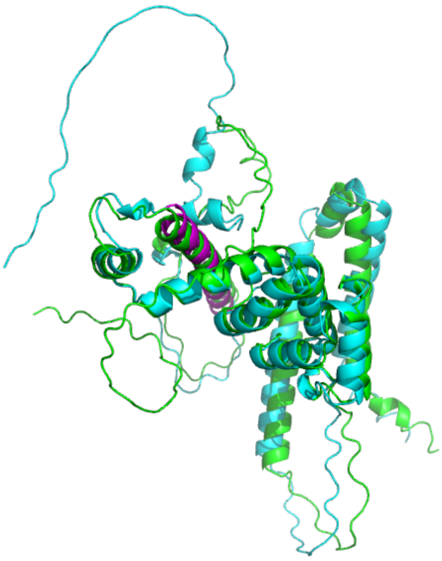

MFD5493388_aligned_scene

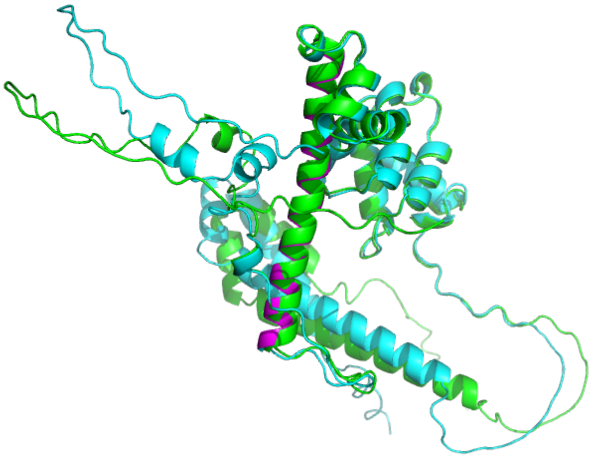

MFD6855123_aligned_scene

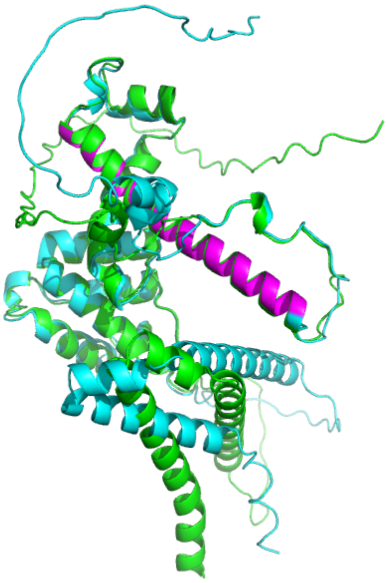

MFD9087792_aligned_scene

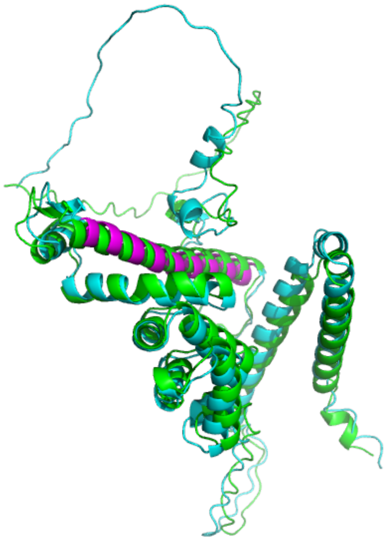

MFD9496864_aligned_scene

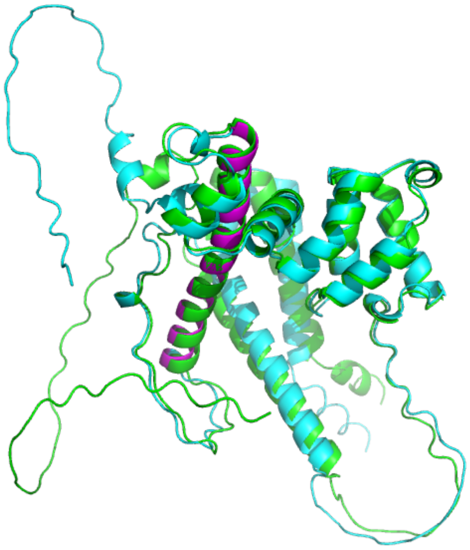

MFE2768756_aligned_scene

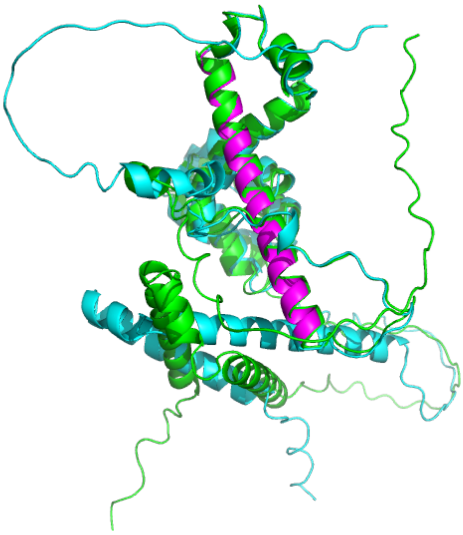

MFE7365913_aligned_scene

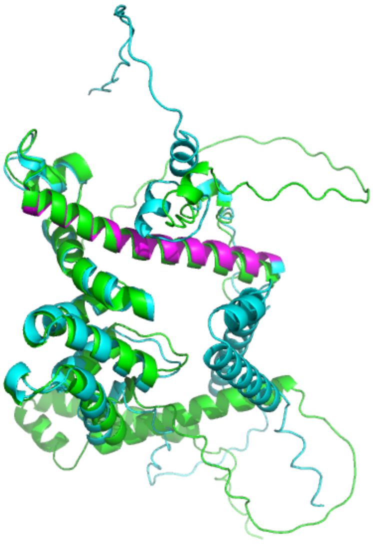

MFE9783647_aligned_scene

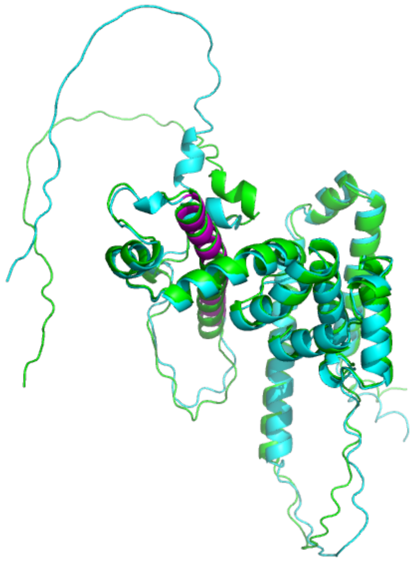

MFF0164033_aligned_scene

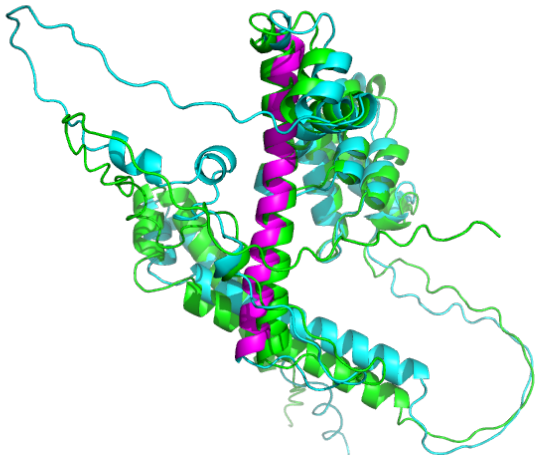

MFF3061566_aligned_scene

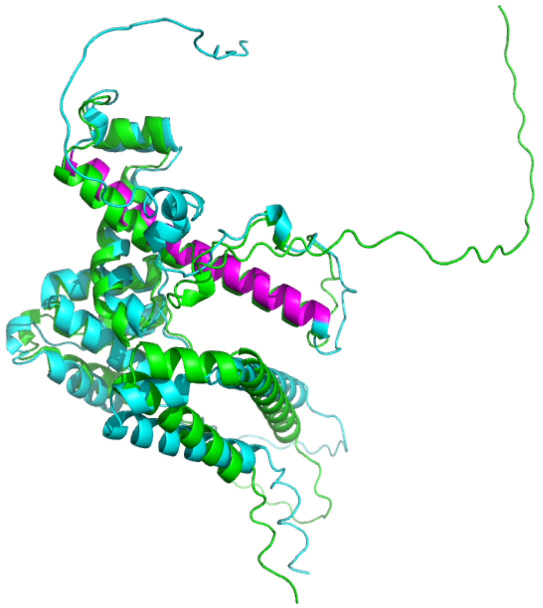

MFF4337950_aligned_scene

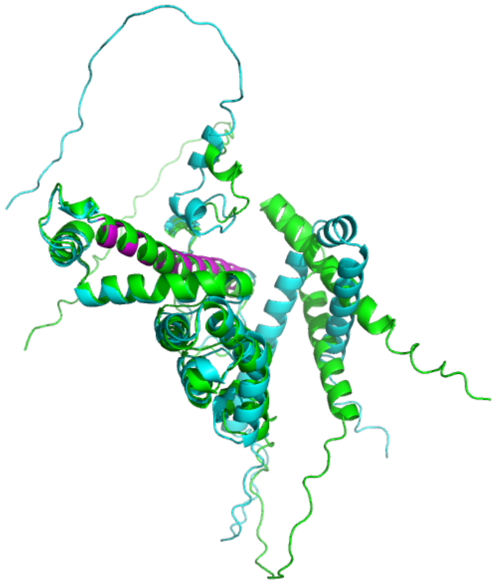

MFF5379426_aligned_scene

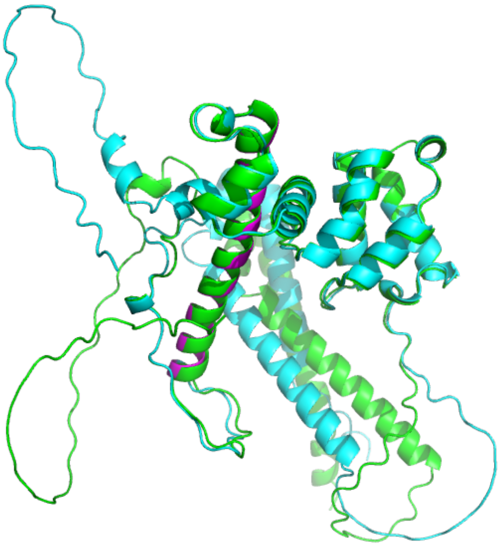

MFF5695044_aligned_scene

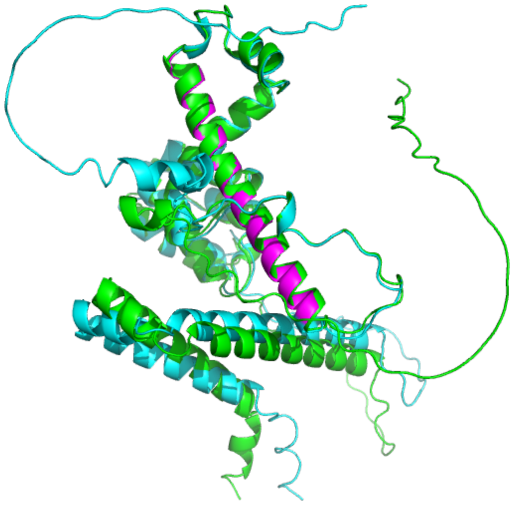

MFF8925554_aligned_scene

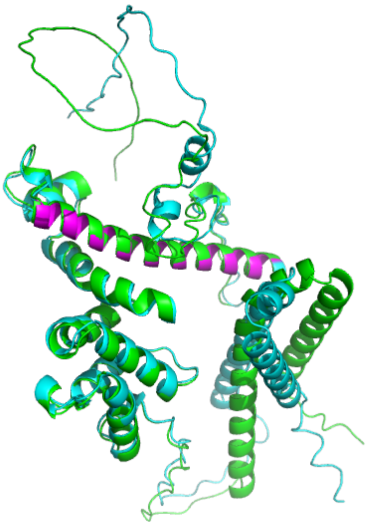

MFH8797669_aligned_scene

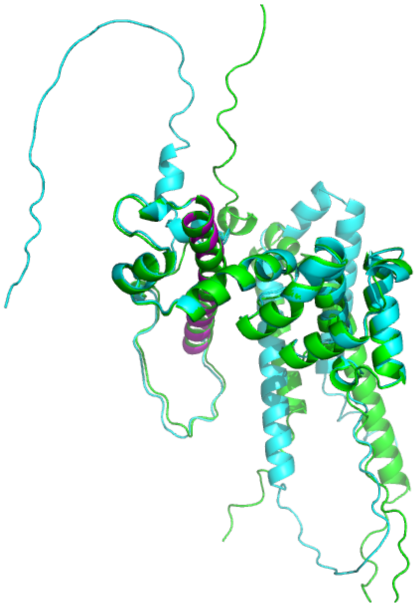

MFJ6076391_aligned_scene

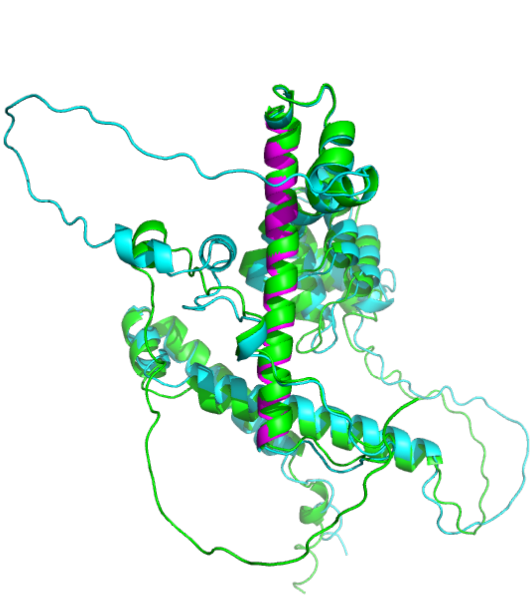

MFJ7497838_aligned_scene

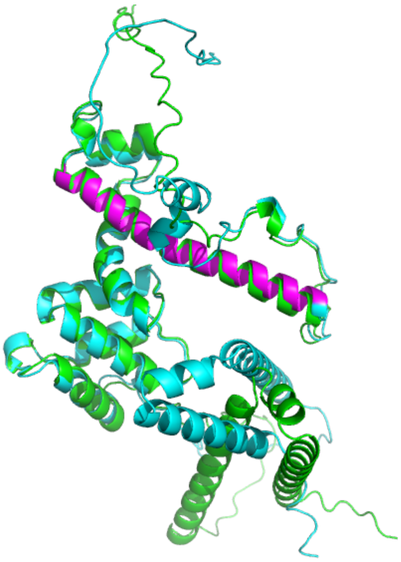

MFJ9662875_aligned_scene

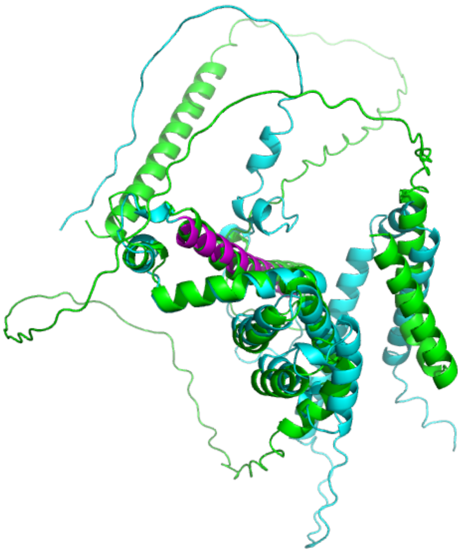

MQS17549_aligned_scene

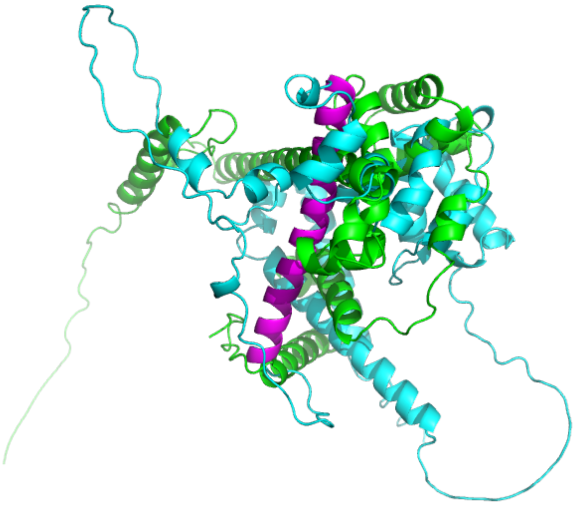

MYW04084_aligned_scene

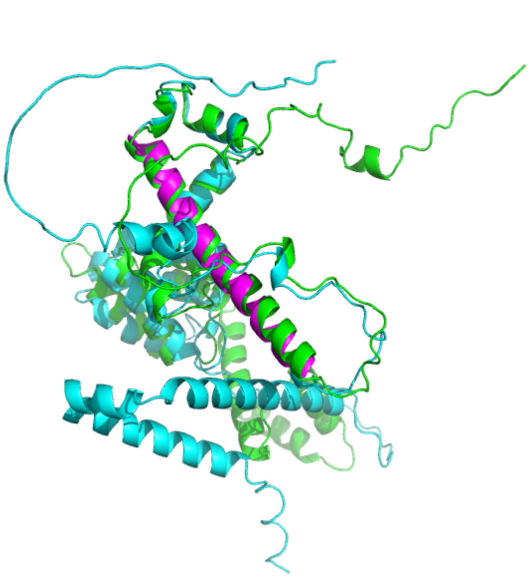

MZE50717_aligned_scene

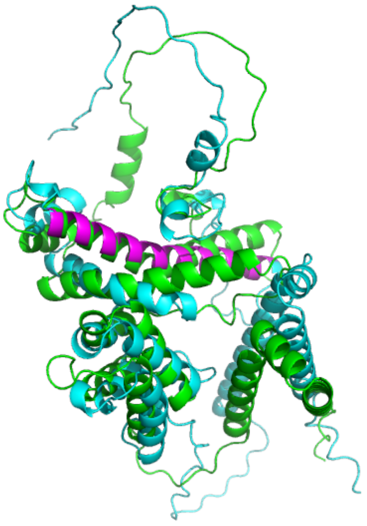

MZE72648_aligned_scene

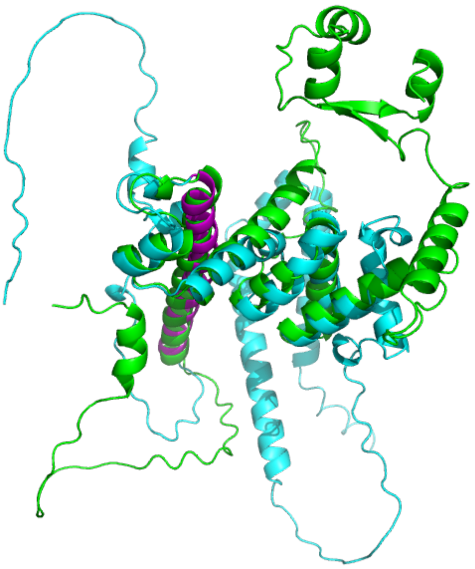

NEU70649_aligned_scene

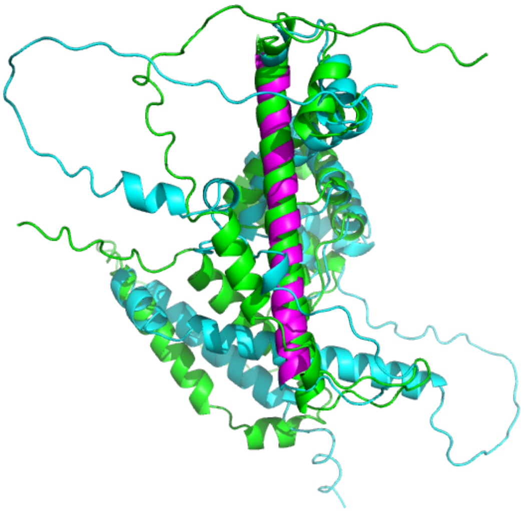

NUV64299_aligned_scene

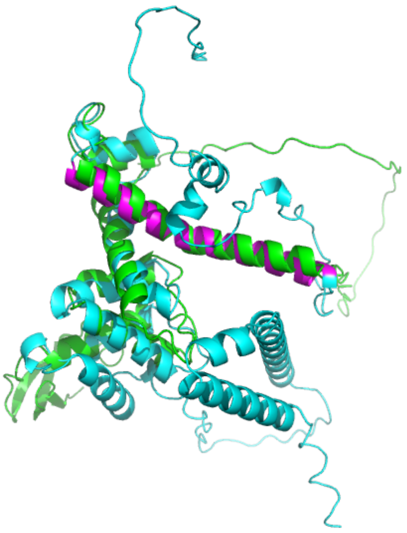

ODG99184_aligned_scene

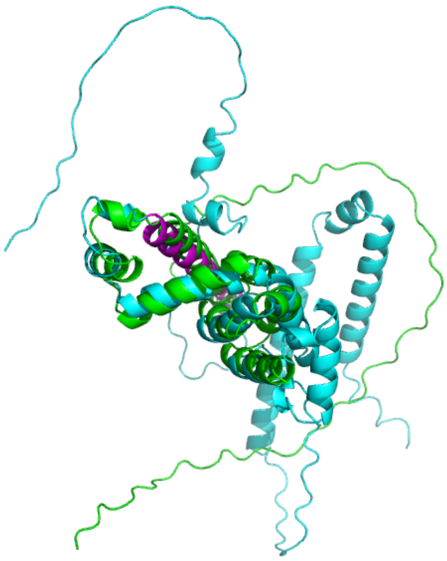

OHV62230_aligned_scene

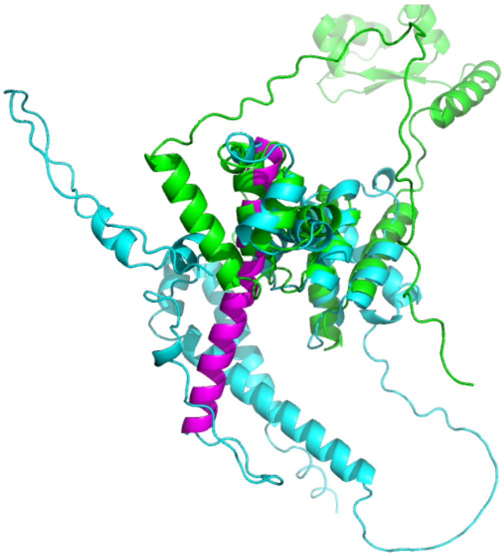

OIN57688_aligned_scene

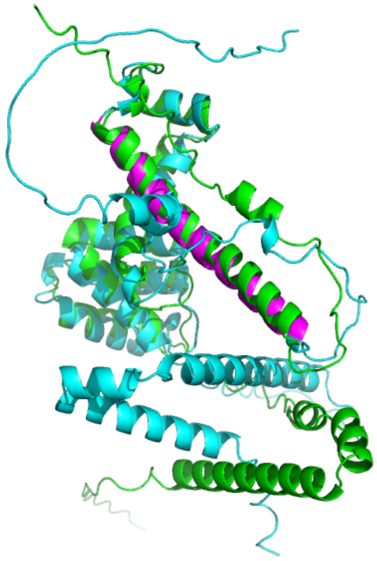

OPC78945_aligned_scene

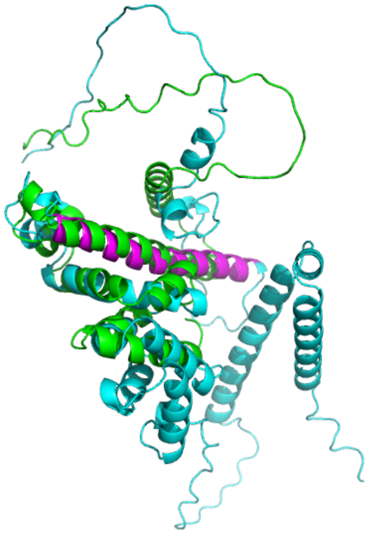

OPG01964_aligned_scene

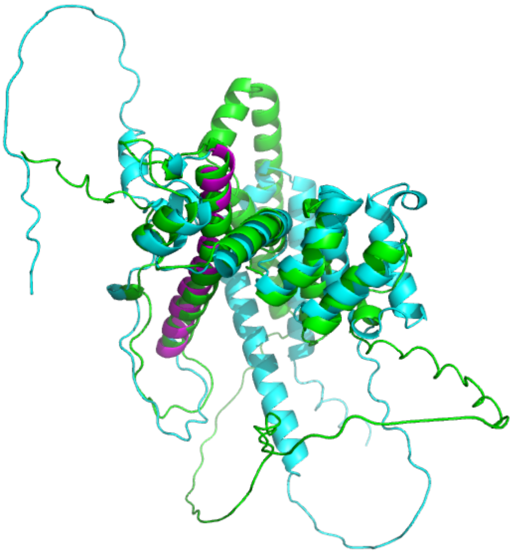

ORT54200_aligned_scene

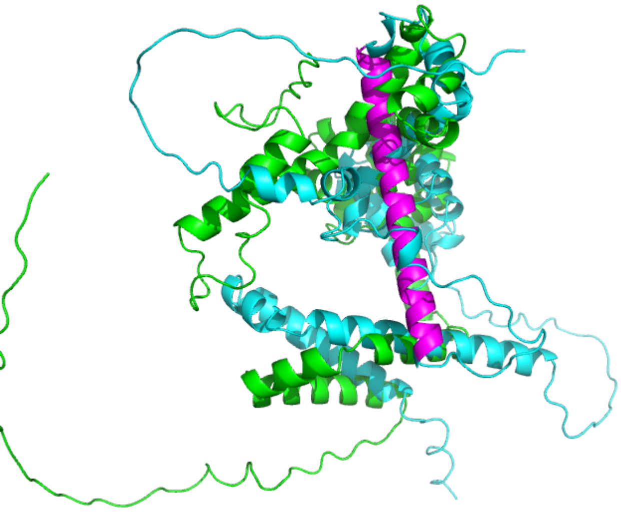

ORT54633_aligned_scene

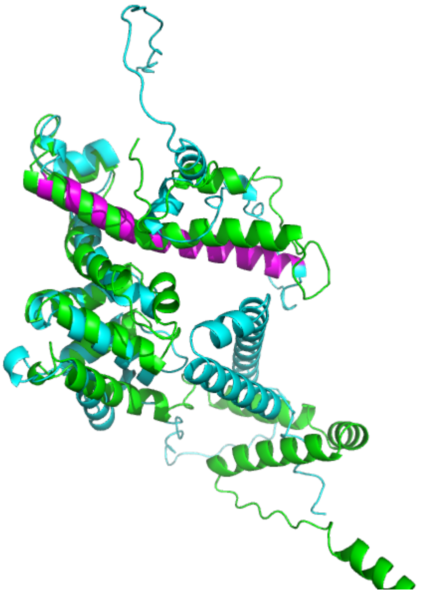

OYP10266_aligned_scene

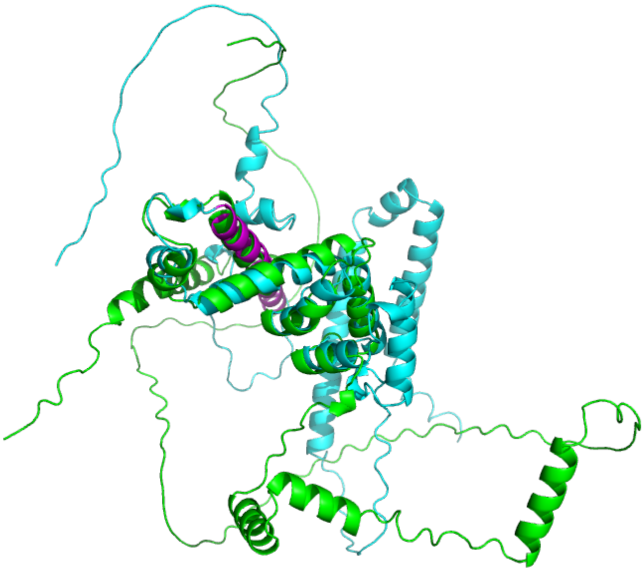

PHX57034_aligned_scene

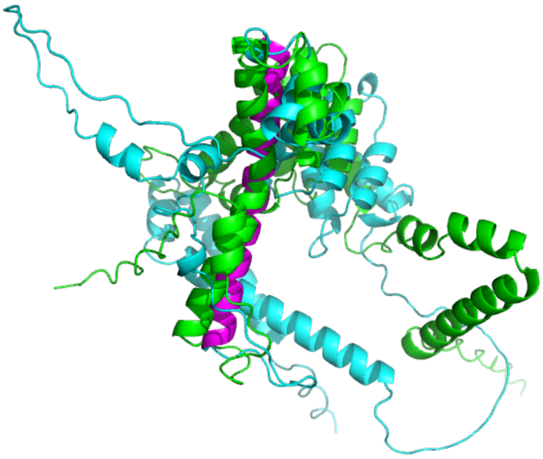

PJE97143_aligned_scene

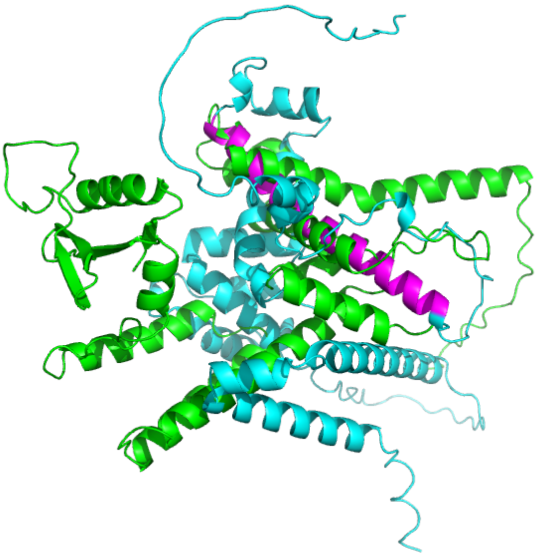

PLX49148_aligned_scene

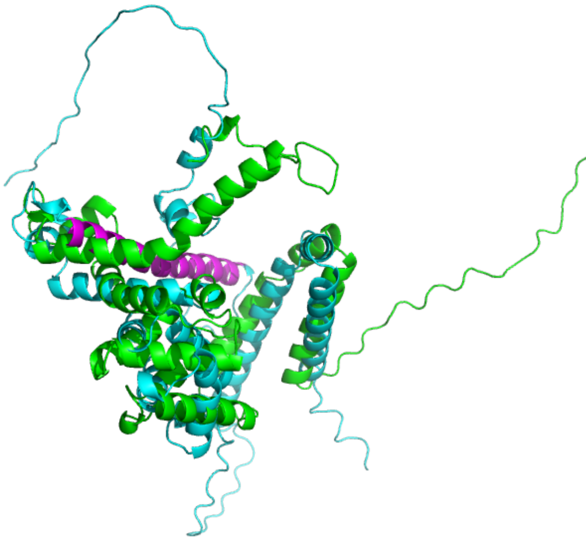

PNG21058_aligned_scene

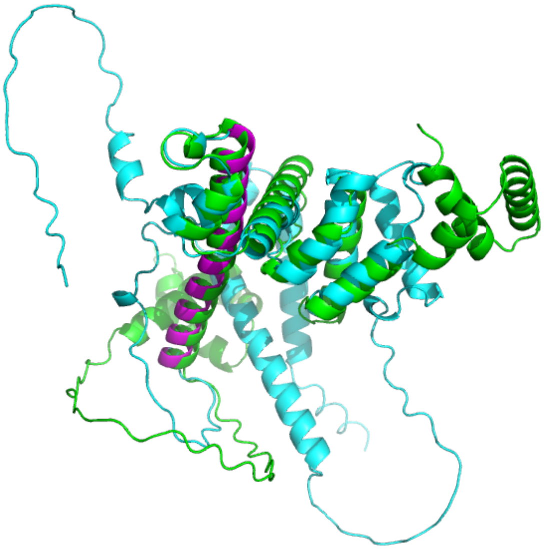

PXA05730_aligned_scene

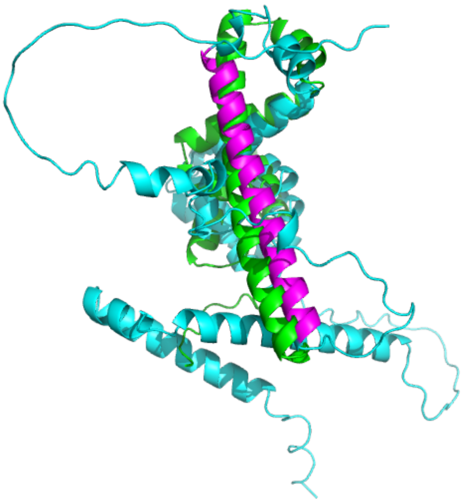

PZP82941_aligned_scene

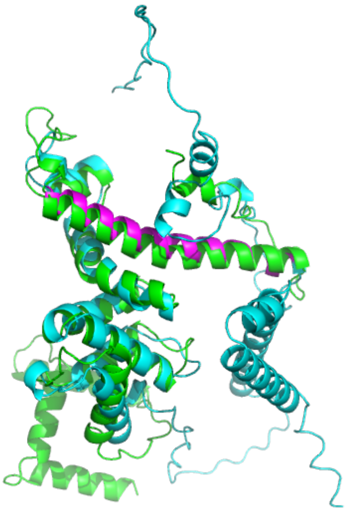

QCX82950_aligned_scene

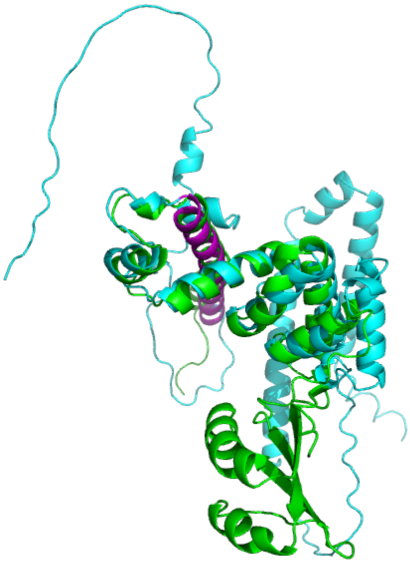

QDT24345_aligned_scene

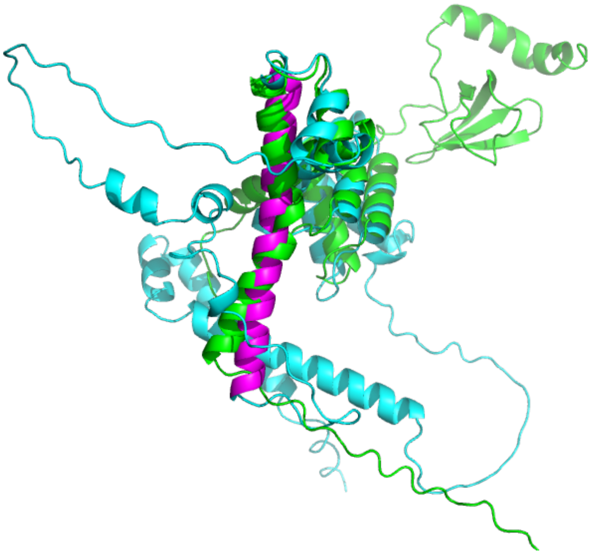

QFS50917_aligned_scene

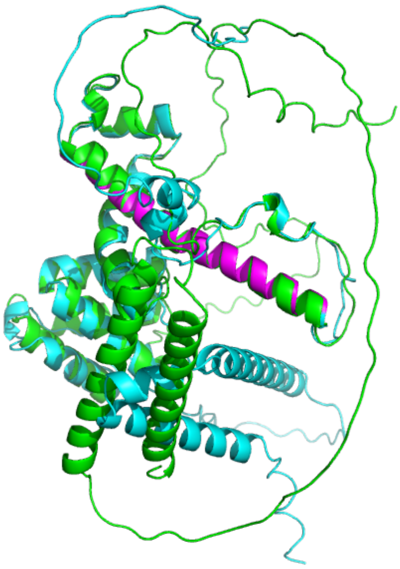

RAG82433_aligned_scene

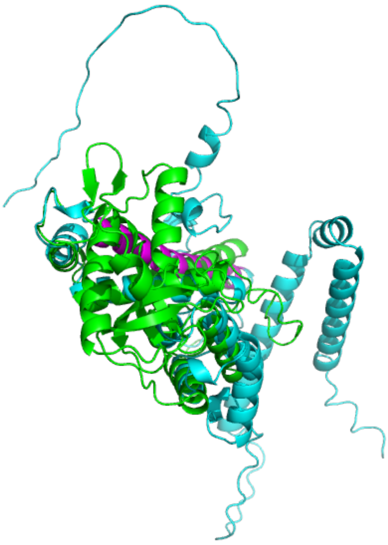

RCJ40808_aligned_scene

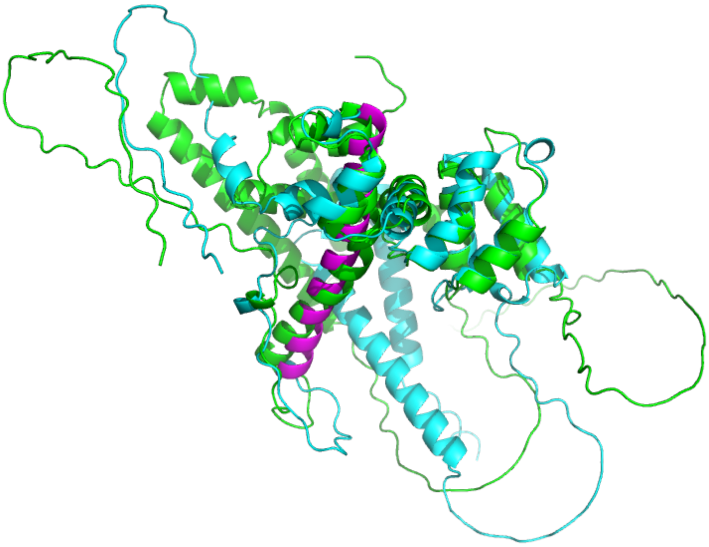

RII07948_aligned_scene

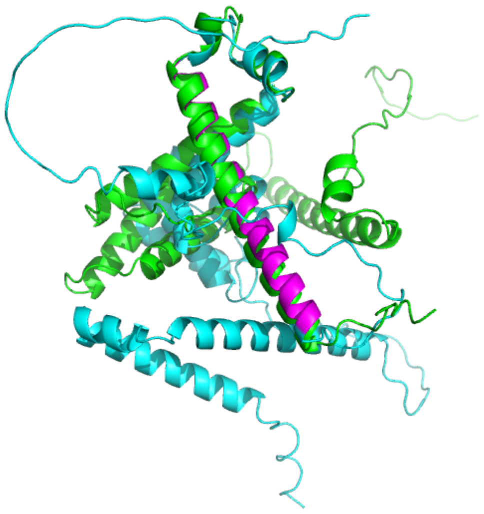

RPE28009_aligned_scene

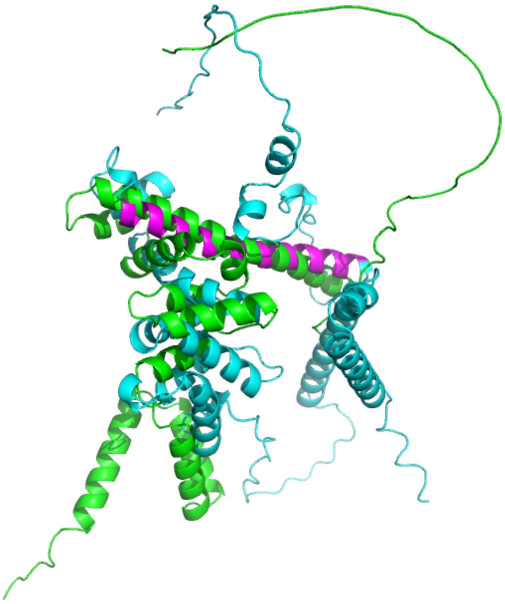

RQH13610_aligned_scene

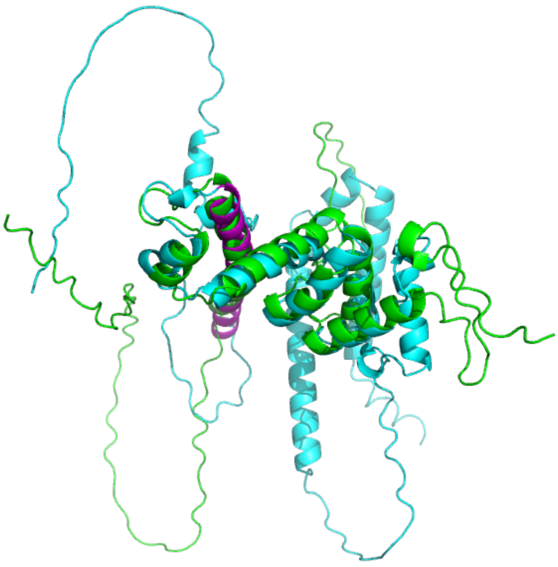

RUR75950_aligned_scene

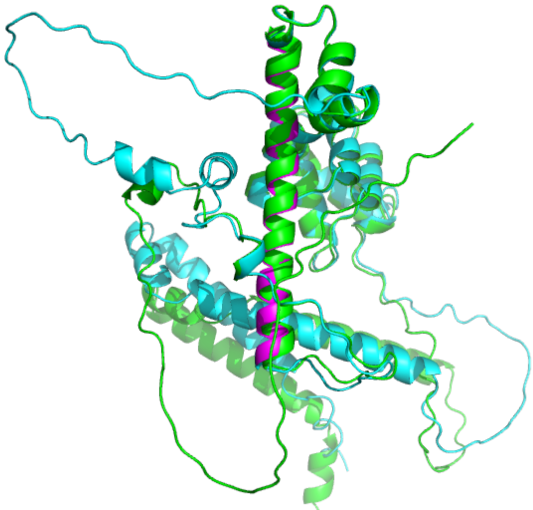

RZE14676_aligned_scene

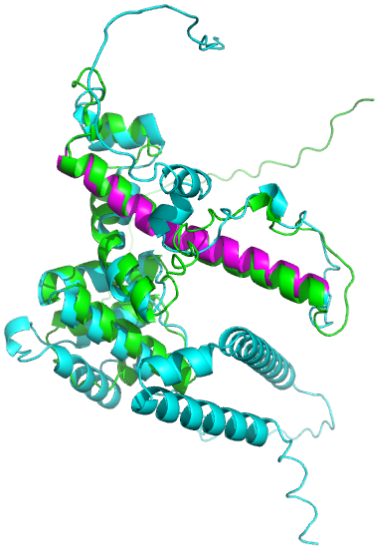

SCD62297_aligned_scene

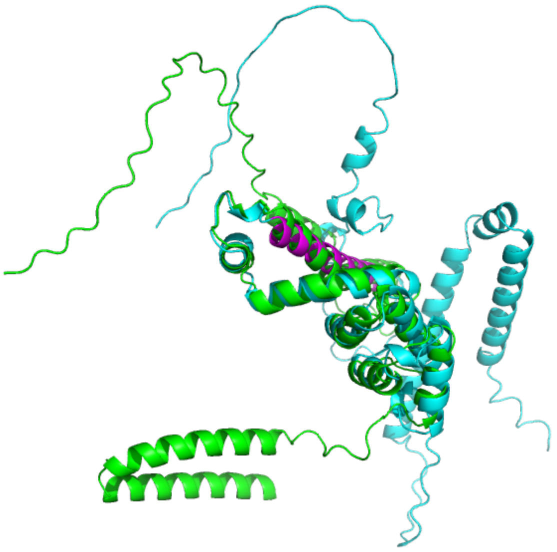

SDI51430_aligned_scene

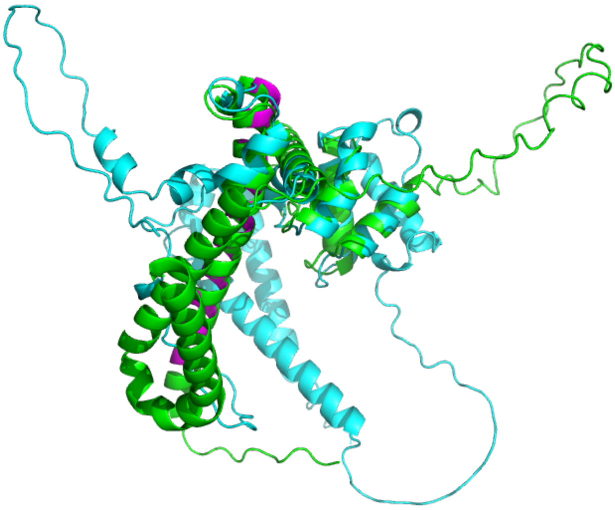

SDT83884_aligned_scene

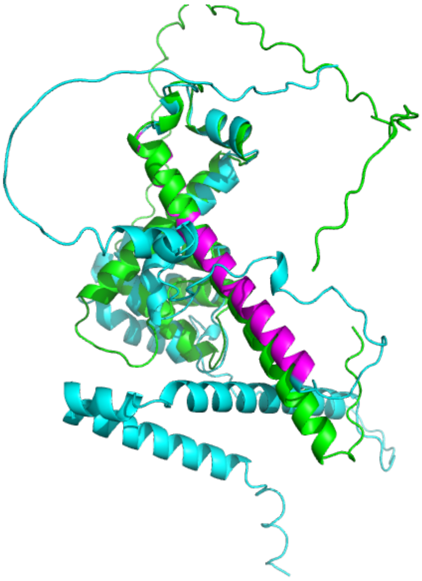

SDT87220_aligned_scene

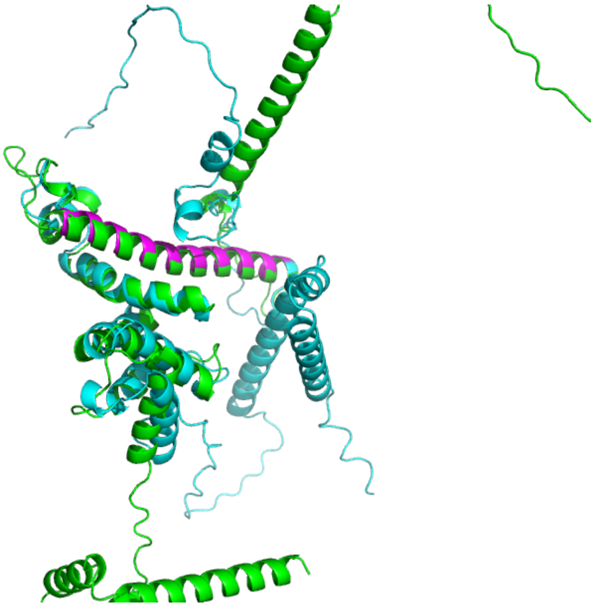

SEE83400_aligned_scene

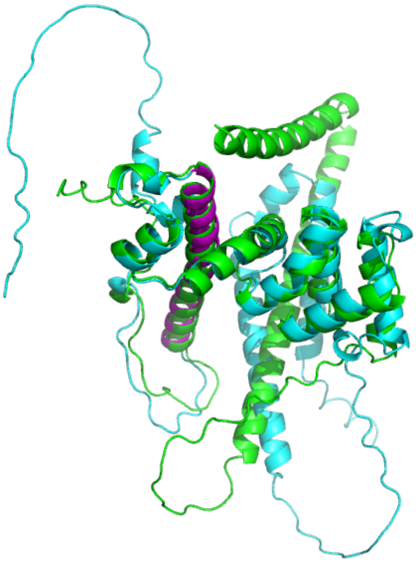

SOD68032_aligned_scene

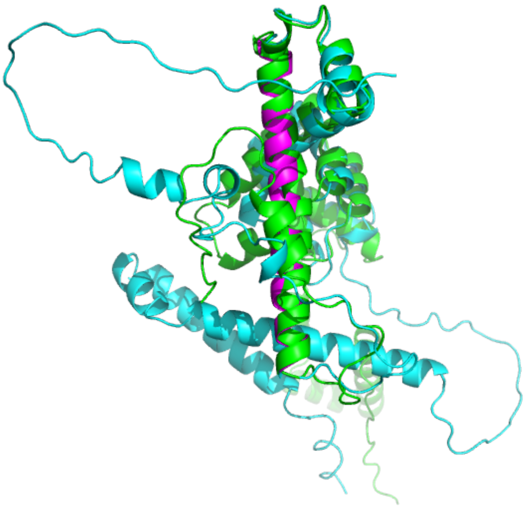

SOD68039_aligned_scene

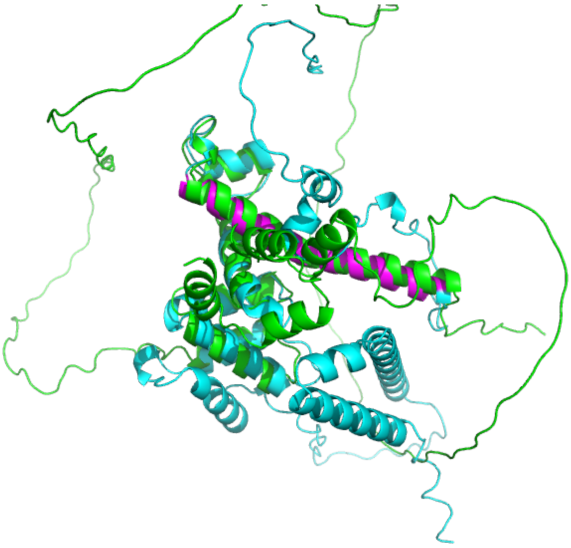

TDB86448_aligned_scene

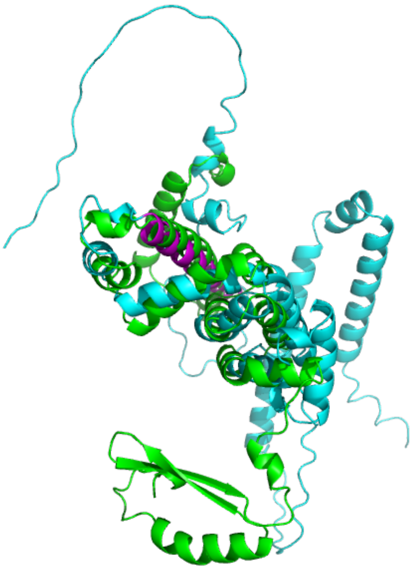

TDC61788_aligned_scene

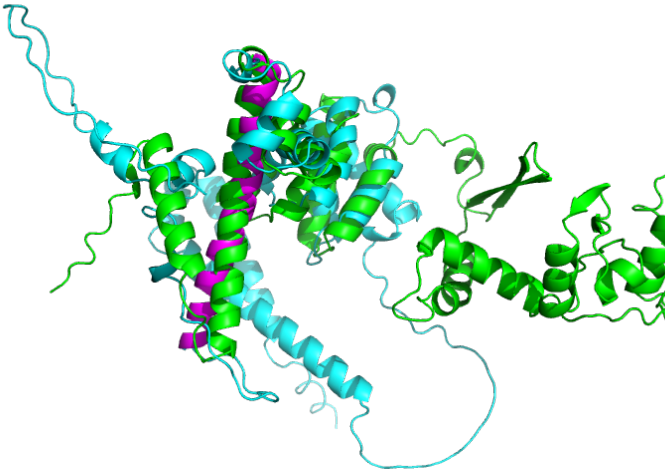

TDC90454_aligned_scene

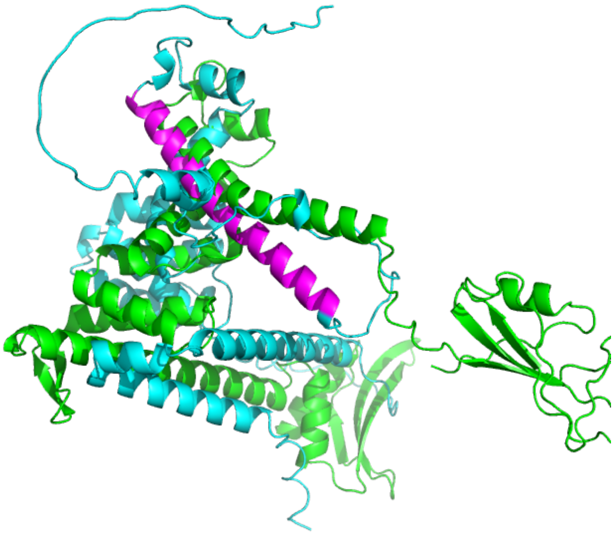

TDE85313_aligned_scene

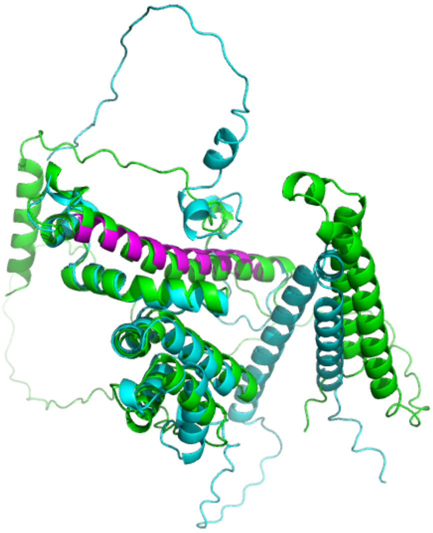

TJZ98262_aligned_scene

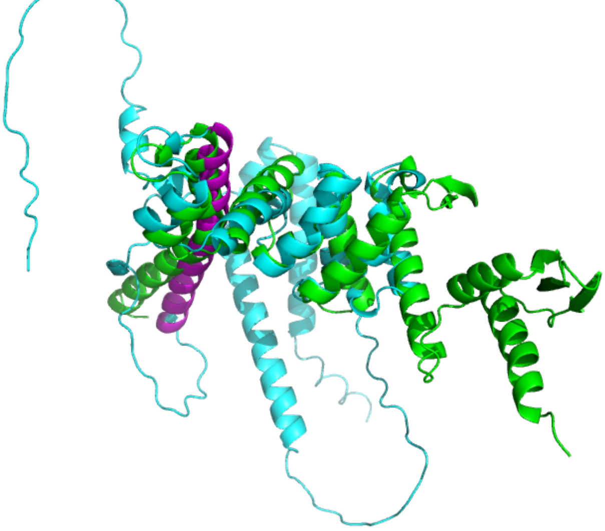

TNM71929_aligned_scene

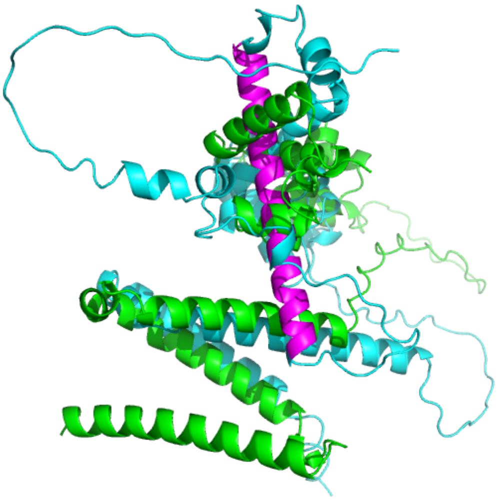

TVZ96473_aligned_scene

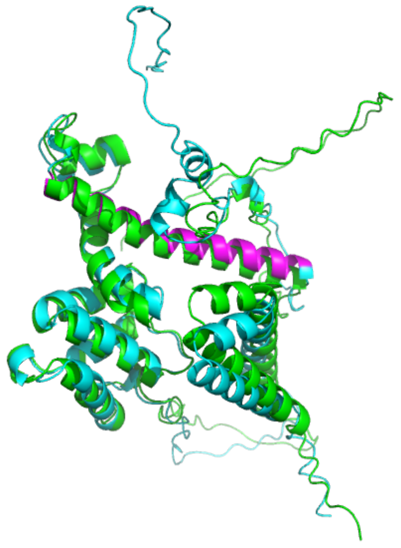

WSM19179_aligned_scene

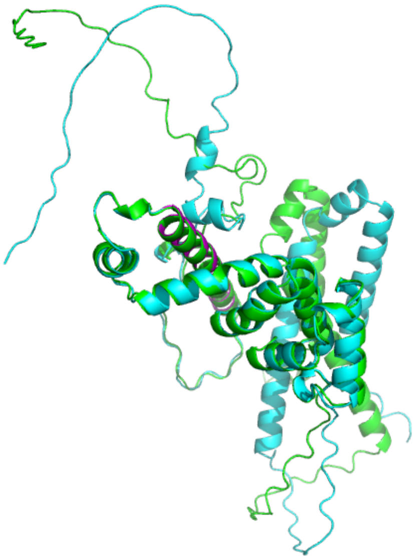

WSN23260_aligned_scene

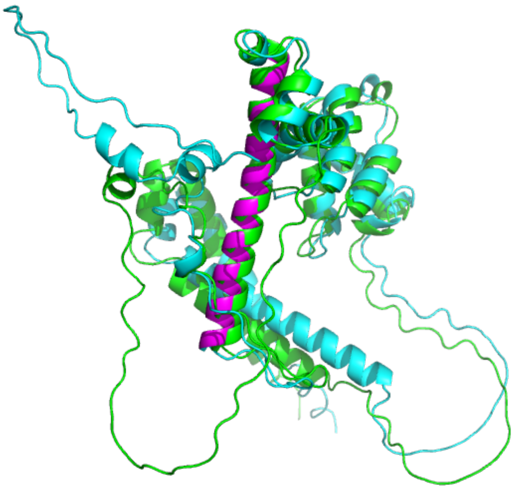

WSS73678_aligned_scene

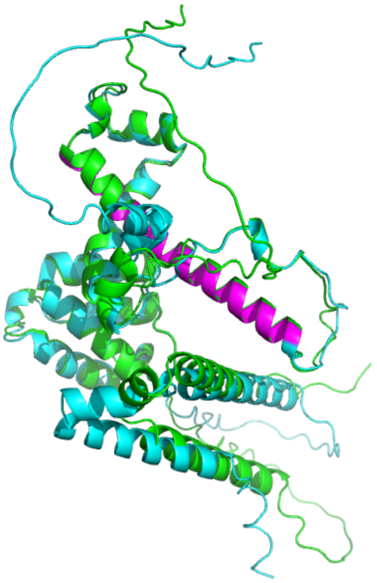

WTY47656_aligned_scene
